# A Review of Image-Based Simulation Applications in High-Value Manufacturing

**DOI:** 10.1007/s11831-022-09836-2

**Published:** 2023-01-18

**Authors:** Llion Marc Evans, Emrah Sözümert, Bethany E. Keenan, Charles E. Wood, Anton du Plessis

**Affiliations:** 1 Faculty of Science and Engineering, Swansea University, Swansea, SA1 8EN UK; 2 United Kingdom Atomic Energy Authority, Culham Science Centre, Abingdon, Oxfordshire OX14 3DB UK; 3 Cardiff School of Engineering, Cardiff University, Cardiff, CF24 3AA UK; 4 School of Mechanical & Design Engineering, University of Portsmouth, Portsmouth, PO1 3DJ UK; 5 Object Research Systems, Montreal, H3B 1A7 Canada; 6 Research Group 3DInnovation, Stellenbosch University, Stellenbosch, 7602 South Africa

## Abstract

Image-Based Simulation (IBSim) is the process by which a digital representation of a real geometry is generated from image data for the purpose of performing a simulation with greater accuracy than with idealised Computer Aided Design (CAD) based simulations. Whilst IBSim originates in the biomedical field, the wider adoption of imaging for non-destructive testing and evaluation (NDT/NDE) within the High-Value Manufacturing (HVM) sector has allowed wider use of IBSim in recent years. IBSim is invaluable in scenarios where there exists a non-negligible variation between the ‘as designed’ and ‘as manufactured’ state of parts. It has also been used for characterisation of geometries too complex to accurately draw with CAD. IBSim simulations are unique to the geometry being imaged, therefore it is possible to perform part-specific virtual testing within batches of manufactured parts. This novel review presents the applications of IBSim within HVM, whereby HVM is the value provided by a manufactured part (or conversely the potential cost should the part fail) rather than the actual cost of manufacturing the part itself. Examples include fibre and aggregate composite materials, additive manufacturing, foams, and interface bonding such as welding. This review is divided into the following sections: Material Characterisation; Characterisation of Manufacturing Techniques; Impact of Deviations from Idealised Design Geometry on Product Design and Performance; Customisation and Personalisation of Products; IBSim in Biomimicry. Finally, conclusions are drawn, and observations made on future trends based on the current state of the literature.

## Introduction

Image-Based Simulation (IBSim) or modelling can have differing meanings depending on the context. In the case of this review, we define IBSim as “engineering simulations based on 3D geometry captured by some form of imaging technique”.

This review focuses on applications of IBSim within high-value manufacturing (HVM), where the simulation techniques typically used are Finite Element Analysis (FEA)^1^ or Computational Fluid Dynamics (CFD), but IBSim can include use of any geometrically based numerical method. That is, improving the accuracy of engineering simulations with the use of ultra-high resolution non-idealised model geometries which estimate the performance of components ‘as manufactured’ rather than ‘as designed’. In this context, IBSim does not mean 1D modelling (or systems modelling) based on measurements obtained by imaging as input parameters, e.g., performing image analysis of a video monitoring the flow of raw material stock to provide measurement data for use in an algorithm which estimates product yield during processing. IBSim is considered an aspect of ‘digital twin’ technology being developed for the smart manufacturing methods of Industry 4.0.

**Fig. 1:**
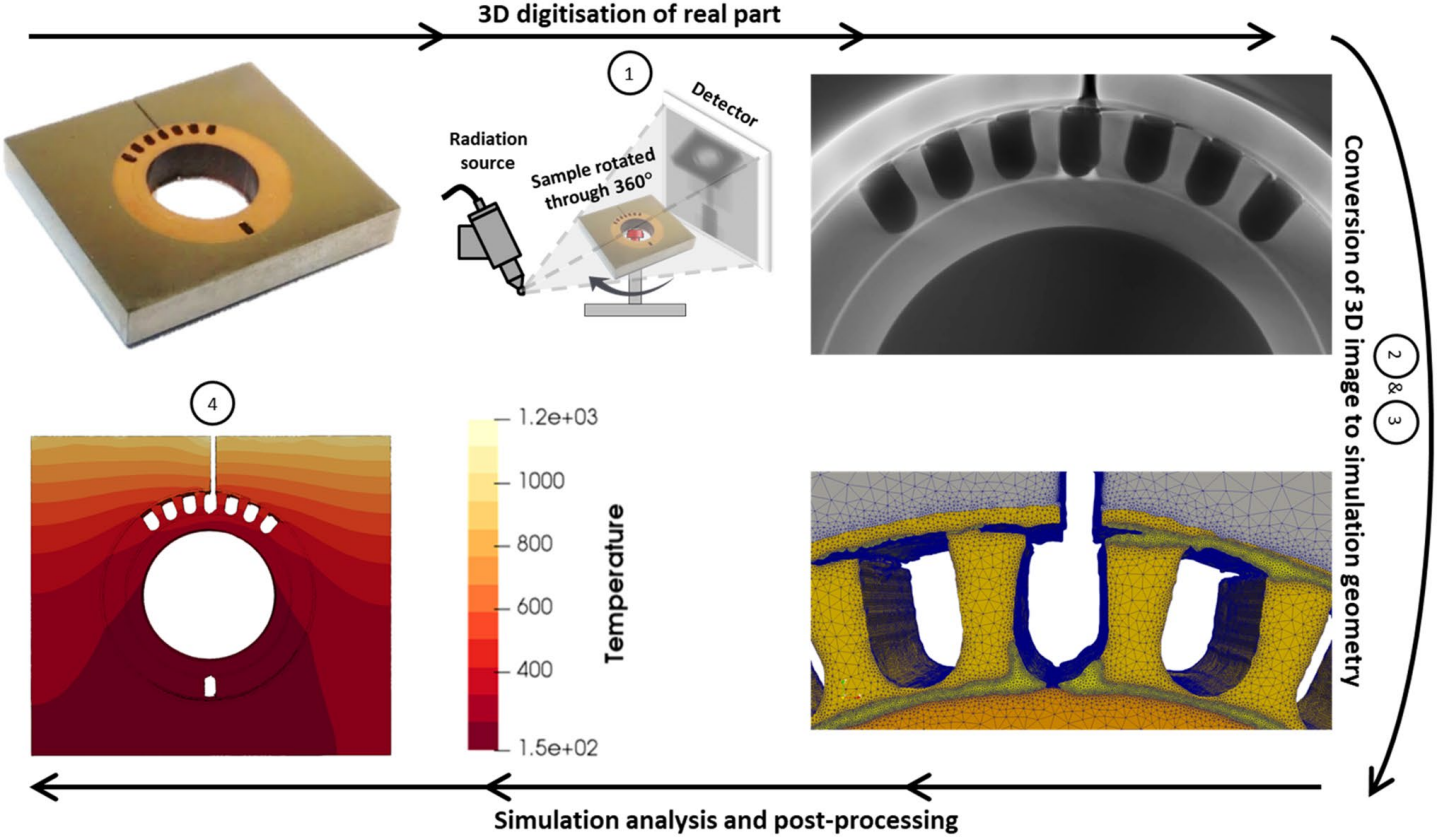
Schematic showing the broad stages for an IBSim workflow which, in this instance, converts X-ray Computed Tomography data into an FEA simulation. This example is a metallic component from a heat exchanger, where the geometry and quality of bonding at the interfaces are integral to the part’s thermal performance

The IBSim workflow can be broadly divided into four main stages as shown in Fig. 1:

1. Digitisation of parts through a volumetric or surface imaging technique.
2. Conversion of the image into virtual geometry.
3. Preparation of the geometry into a simulation ready format.
4. Image-Based Simulation, visualisation, and post-processing.

Due to these different stages, it is a highly multidisciplinary process involving the fields of microscopic imaging, image analysis, high performance computing and data science, engineering simulations, material science and increasingly machine learning. The combination of such a broad field of disciplines can in itself be a challenge and barrier to adopting IBSim. The initial stage, i.e., 3D scanning techniques for producing a volumetric or surface image, can range from a topological scan using methods such as:

- Laser scanning
- Structured light scanning
- Ultrasound
- Photogrammetry
- CMM

To full 3D mapping with techniques like:

- Computed Tomography (CT)
- Magnetic Resonance Imaging (MRI)
- Confocal Microscopy
- Optical Serial Sectioning Microscopy (OSSM)
- Focussed Ion Beam-Scanning Electron Microscopy (FIB-SEM)
- Serial Block-Face Scanning Electron Microscopy (SBF-SEM)
- Transmission Electron Microscopy (TEM)

Each technique has its own strengths and limitations and usually the size and material of the item being imaged, and the context will dictate which method is most appropriate. The resolution of the IBSim geometry will inevitably depend upon the resolvability of features within the image on which it is based. It is, therefore, essential to select the most appropriate technique and acquire the best resolution feasible for the given circumstances. Although some corrections may be applied with image-processing methods, there are no replacements for following best practices for the imaging technique of choice. The most widely used acquisition method is CT, due to its non-destructive nature, combined with high resolution capabilities matching well the typical requirements for IBSim investigations.

Once the 3D data has been acquired it must be converted into a virtual representation of the geometry to allow simulations to be run. Topological scans, e.g., from laser scanning, are the simplest forms of imaging data to create IBSim models. Since they only capture the external geometry with no internal features (e.g., micro-pores or inclusions), they are relatively small datasets, but still significantly larger than Computer Aided Design (CAD)-based geometries.

These will often be formed of point clouds, which are a collection of cartesian coordinates representing the sample surface. Techniques like photogrammetry can provide additional information such as colour, which facilitates distinguishing between materials in multi-phase samples. Post-processing methods are used to interpolate between the points and define surfaces. Smoothing algorithms are often used to ‘clean’ data and remove spurious points or fill in voids in the data.

**Fig. 2:**
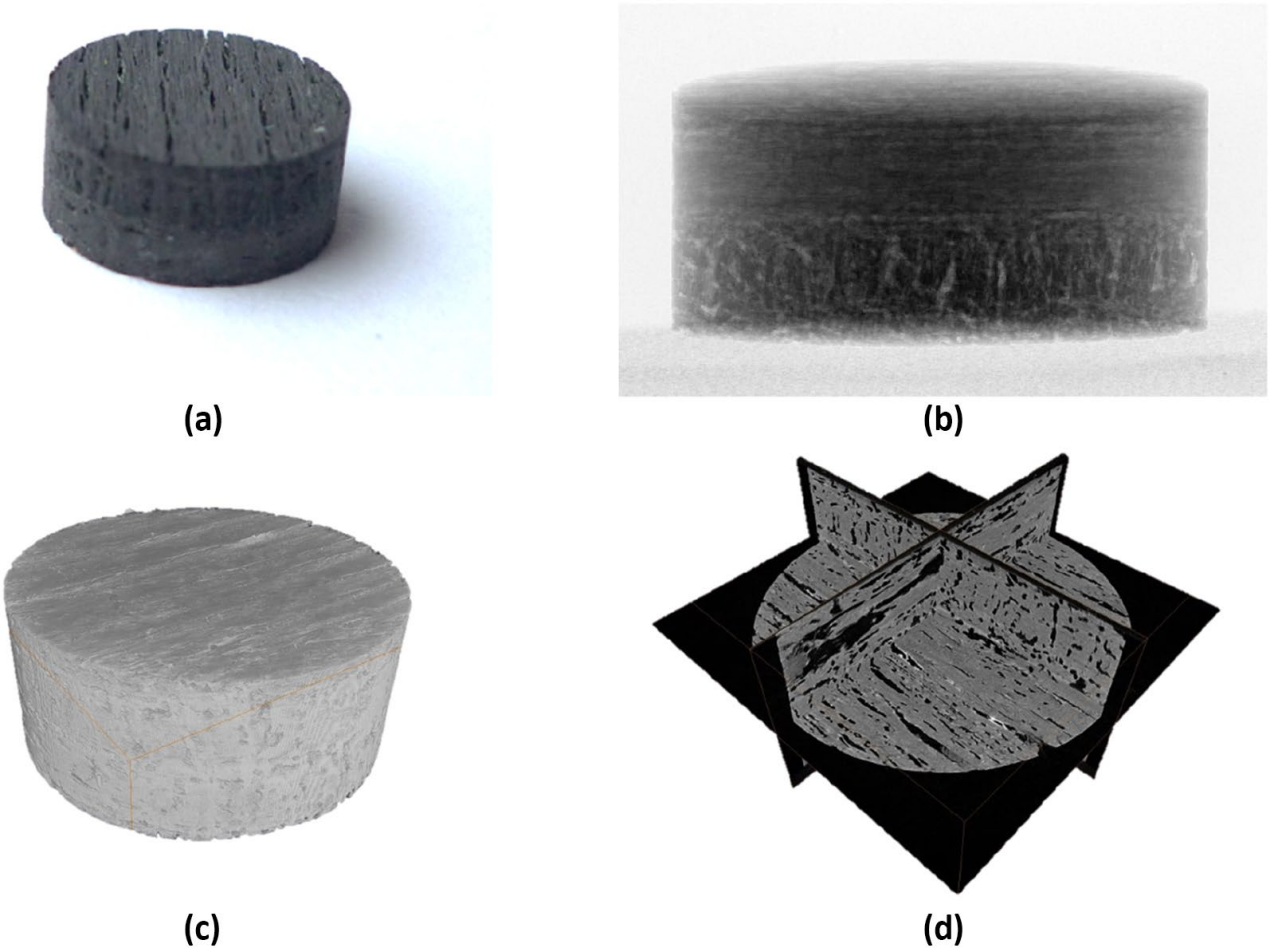
Visualisations of a carbon fibre-carbon matrix composite: **a** photograph, **b** X-ray radiograph, **c** volume rendering of XCT data, **d** 3 orthoslices in the xy, xz and yz planes from XCT data

Full 3D volumetric images are data-rich and, depending on the imaging method, can include features of interest that are less than 1/1000th the size of the parent sample. The images typically consist of a discretised voxel domain, with each voxel (3D pixel) providing some information about that location in space. For example, in conventional X-ray CT (XCT), a voxel provides information about signal attenuation at that location that can be used to infer material density [1]. When the data is rendered as an image, rather than a three-dimensional matrix, the attenuation is visualised by being assigned a given colour or grey scale. This can be visualised with volume rendering or with 2D images as cross-sectional slices through the part, as shown in Fig. 2.

**Fig. 3:**
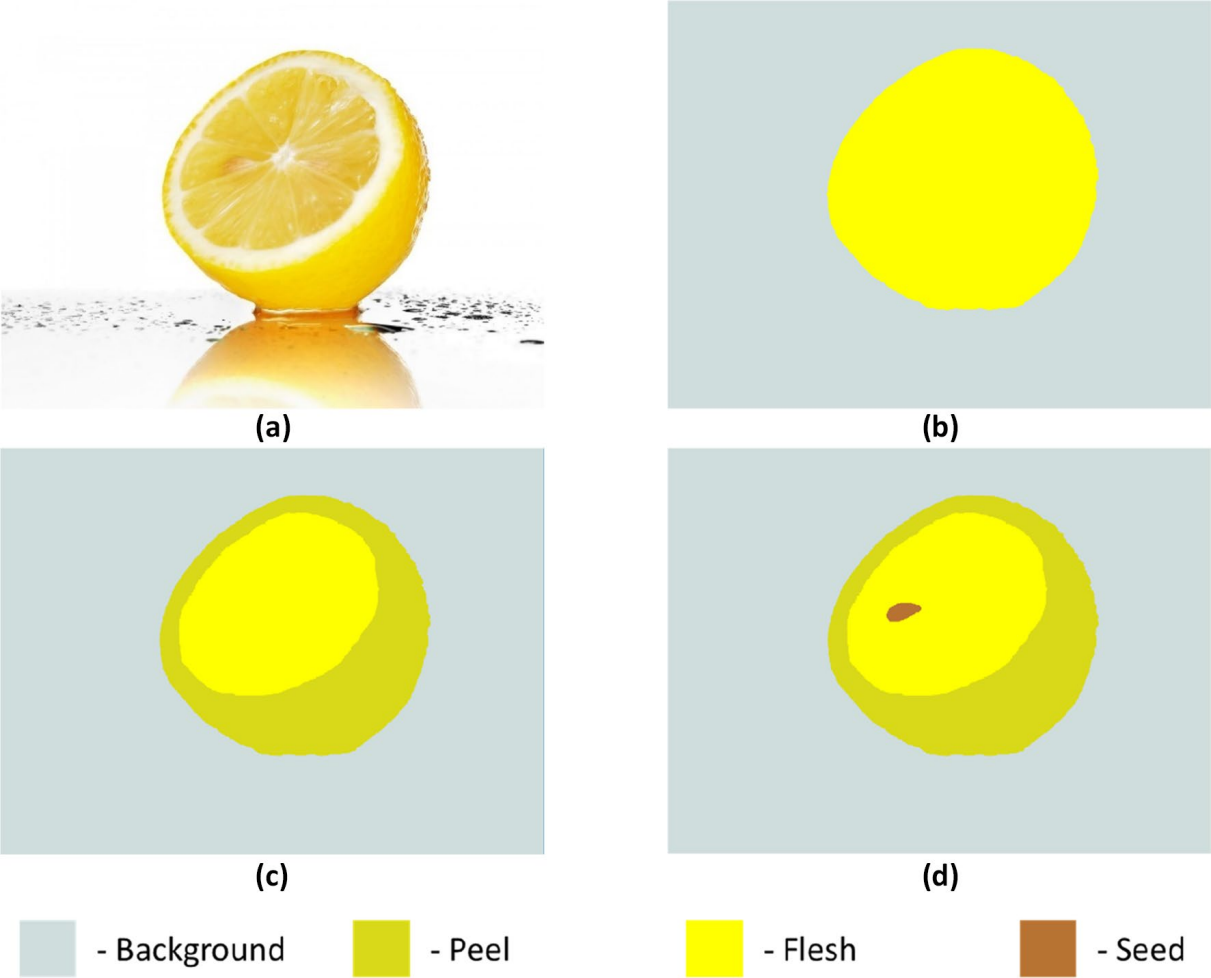
Schematic demonstrating various levels of detail possible when segmenting a complex object. **a** Photograph of a lemon cut in half and **b**–**d** image segmented into increasing number of phases: **b** background, fruit; **c** background, peel, interior; **d** background, peel, flesh, seed

A segmented volumetric image will still consist of a voxelised domain, but with each voxel having a phase number rather than greyscale value. Fig. 3 shows examples for an image of a lemon fruit, segmented to increasing level of detail. In addition to the examples shown in Fig. 3b−d it may be possible to segment many more phases up to what is resolvable with the available image resolution (e.g., separating the albedo and flavedo).

Many segmentation approaches and software solutions exist, from fully manual voxel ‘painting’ on a slice-by-slice basis to semi and fully automated methods assisted by image processing algorithms [2, 3]. In practice, more complex images (i.e., sample geometry, number of phases, level of noise, and artefacts) tend to require more manual interaction.

Once a voxel geometry has been defined, it is possible to perform simulation analysis directly on this data. This is often the approach of ‘first pass’ or ‘rapid turnaround’ mesh-based methods by using the voxels as hexahedral elements, for example with the Finite Difference Method. For more in-depth analysis with mesh-based methods, such as FEA and CFD, it is usually desirable to perform some preparatory steps such as smoothing, mesh validity and quality checks, and mesh refinement and/or partitioning.

If the preparation of the IBSim mesh has been carried out effectively, the running of the actual FEA/CFD analysis should not differ significantly from a CAD-based simulation. There are still some considerations worth noting. The main additional consideration should be how to work with data volumes which are orders of magnitude greater than conventional CAD-based models. This includes use of computing hardware of sufficiently high enough specifications (CPU cores, RAM, GPU) and software workflows (usually parallelised) that make efficient use of this hardware for simulation, data analysis and visualisation.

As previously stated, the focus of this review is IBSim applications within HVM, and at this juncture it is worth providing a disambiguation for the commonly misused term ‘HVM’. A report by the Institute for Manufacturing, Cambridge [4] states that a simple definition of HVM is not possible and, rather, sets out a framework by which to contextualise it. Their framework broadly defines four types of manufacturing which can be considered ‘high-value’: service led producers; product manufacturers; service manufacturers; and system integrators. They explicitly make a distinction between manufacturing and production “A key point in defining HVM is that manufacturing is not production and vice versa.”. That is, if used for its true meaning, HVM also includes stages from research and development (R&D) to ongoing post-production services (in both the physical and digital realms).

For that reason, HVM is predominantly used in this review to mean the value provided by a manufactured part (or conversely the potential cost should the part fail) rather than the actual cost of manufacturing the part itself. For example, a critical part in a satellite, whose mission value is estimated at hundreds of millions of dollars, might only cost a few hundreds of dollars to manufacture, but the impact of failure could lead to a catastrophic loss. In such circumstances, it is prudent to spend substantial effort in performing non-destructive testing and evaluation (NDT/NDE) to build confidence that the particular part in question will perform as expected. The cost of this effort might be greater than the cost of manufacture. No manufactured parts are ever defect free, if investigated at sufficiently small scales defects are always found to be present. The important outcome of NDE, therefore, is to quantify the ‘effect of the defect’ to build a better understanding of what limits a given part should be operated under. IBSim models are data rich, giving unprecedented insight into localised fluctuation in behaviour due to micro-features as well as their global impact. High resolution visualisation allows researchers to investigate these in detail.

It is worth noting that IBSim’s roots lie in the biomedical field, which can primarily be attributed to the fact that this is also the field that has been a substantial driver for the development of volumetric imaging, such as XCT and MRI. It is difficult to identify the first instances of IBSim, however, early work used external measurements of patients to amend CAD-based models and thus make them patient-specific [5]. This progressed to using internal measurements from volumetric images [6] as XCT and MRI became more prevalent, it subsequently led to full conversions of volumetric images directly into simulation geometries [7]. As could be expected, the use of IBSim within the industrial sector coincided with the increased usage of imaging methods, such as micro computed tomography (μCT), which has seen a growth of greater than 10% year-on-year over the past decade [8]. IBSim is also used in other fields of research, such as geology (largely in relation to the oil & gas industries) [9], archaeology [10], and palaeontology [11]. This review will restrict itself to applications within HVM other than select examples from biomechanics, where a manufactured part is used in the medical field. However, the review should also be useful to readers interested in the other aforementioned fields because there is much in common between the methodologies. This review is the first of its kind for HVM and aims to give a thorough overview of the literature to date rather than an update on recent publications alone.

## Review of HVM Applications of IBSim

IBSim is already being used within research and development (R&D) cycles to accelerate development by providing additional insight at various stages [12–14]. The progress along the R&D cycle of producing a new concept is described by its ‘technology readiness level’ (TRL), which is a method of categorising its maturity stage. These levels range from the conceptual stage (TRL 1) to full production with a proven in-service track record (TRL 9).

To increase efficiency in R&D cycles it is desirable to accelerate progress through the TRLs. Within manufacturing, identifying optimal products (their design, material selection, usage parameters etc.) is achieved by iteratively down-selecting candidates through testing. Much of this development process is constrained by available resources. That is, the number of candidates which may be considered are limited by costs and time. Virtual testing through computational simulation techniques have increasingly been facilitating the R&D process [15]. With simulations it is possible to iterate through many more designs quickly and cheaply without needing to prototype concepts.

**Fig. 4:**
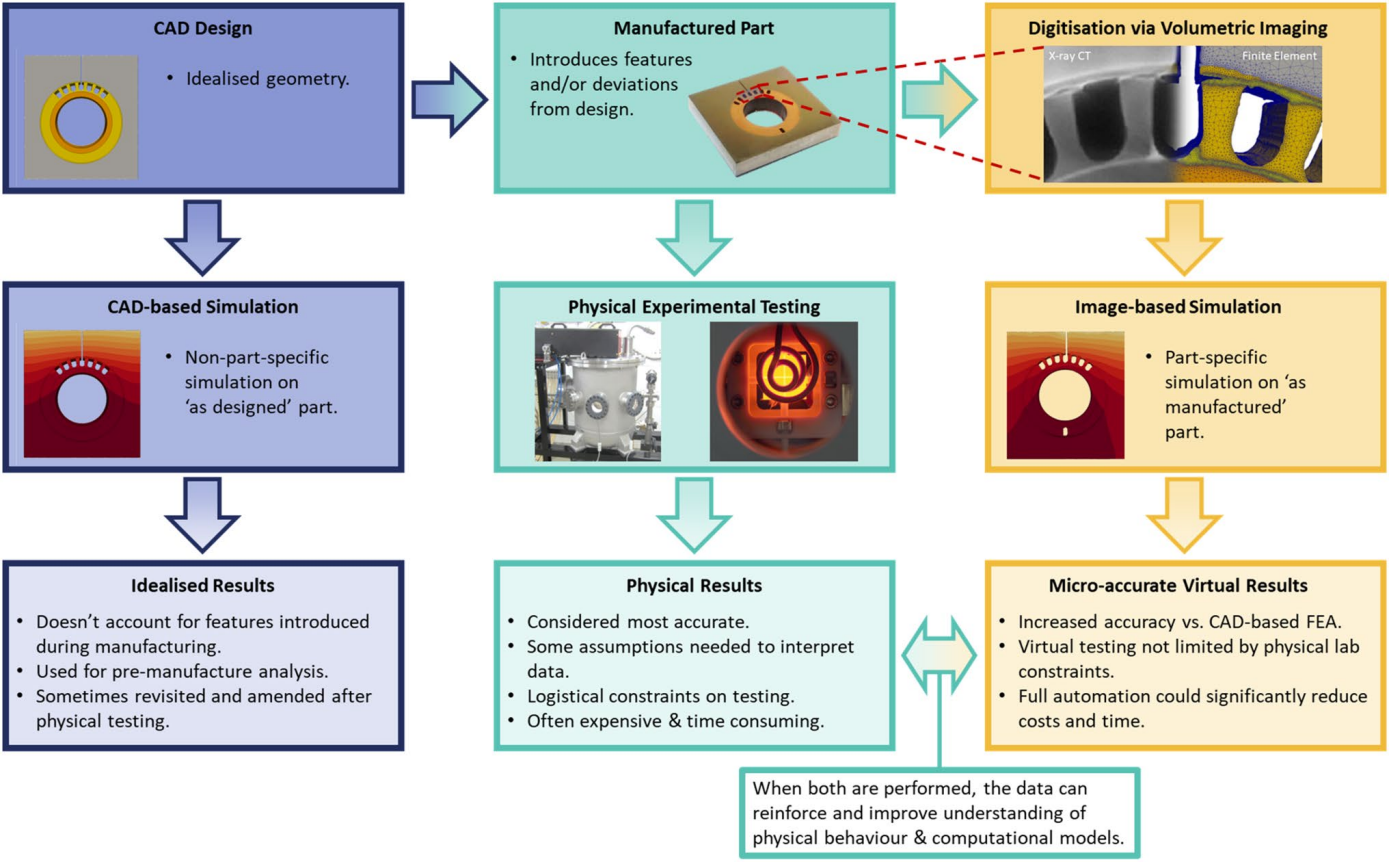
Flowchart demonstrating the relationship between ‘as designed’ simulations and physical testing conventionally used with more novel ‘as manufactured’ virtual testing with IBSim

However, there exists a gap between observations during simulation and experiments [16]. As such, simulations are used as first stage guidance but there is still a heavy reliance on experimental testing during R&D. Improved accuracy in simulations could lead to more rapid R&D development. IBSim is one approach that can close the gap between simulation and experiment [16]. By digitising a real prototype, microscale accurate simulations can be carried out on the part ‘as manufactured’ rather than ‘as designed’. This means its geometry is no longer idealised and simulations account for impact on performance due to manufacturing processes by inherently including features such as deviations from tolerance and micro-porosity. A flowchart is shown in Figure 4.

Within R&D, IBSim allows one manufactured prototype to be tested to destruction multiple times by using a virtual representation of the manufactured part which is faithful on the microscale [17]. In addition to replicating laboratory testing through simulation, it is possible to extrapolate to scenarios more representative of real-world conditions e.g., increased number of cycles, real loading rates and values, complex loading with multiple mechanisms. This is because IBSim testing is not constrained by the limitations of the laboratory. That means much more valuable data can be obtained from a single prototype, significantly reducing costs.

Through being able to directly compare results from experimental and simulation results, IBSim benefits from verified results with increased confidence values compared with simulations using idealised geometries. This is invaluable within the industrial sector when simulating conditions outside what can be tested in the lab. IBSim is also used in materials development to perform virtual characterisation to reduce the number of physical tests and thus the volume of material required.

The process of manufacturing novel materials can often be a rapid process which only requires the variation of some parameters during fabrication. This can create different microstructures which leads to different material properties. However, the process for characterising the new properties of the material can involve a significant effort and thus cost. If it is desired to measure a range of properties, this can require fabrication of many samples for a series of experimental tests.

By using IBSim, it is possible to perform virtual testing with a suite of simulations that emulate laboratory material characterisation from one manufactured block of material that is digitally ‘cut’ to the required dimensions. This way, new materials can be rapidly characterised to identify the strongest candidates. Furthermore, for method and model validation, actual test samples can be replicated digitally for a direct comparison of physical and virtual test results leading to an improved level of confidence.

Because of the digitisation process from a real material, IBSim can be used for simulations of extremely complex geometries such as fibre composites or foams with no need for idealisation. Additionally, it is possible to digitally alter the structure to see what impact this has on the properties. For example, the volume of pores or the thickness of foam ligaments could be increased/decreased to investigate the benefits of imposing additional control on the material processing.

To reflect those categories of IBSim usage within HVM, this review has been structured accordingly. Firstly, IBSim’s use for material characterisation is presented followed by how IBSim has been applied to optimise manufacturing processes. Next, case studies using IBSim to investigate how deviations from idealised design on both the micro and macro scales impact product design and finalised part performance. Also included are two final sections on how IBSim is used in a broader sense to improve the customisation and personalisation of products and how it is contributing to the field of biomimicry in manufacturing. The review is concluded by looking at growing trends in IBSim for HVM that are showing significant promise.

### Material Characterisation

Image-based models and their simulations have been demonstrated to be highly useful in determining morphological features of materials and, consequently, effective material properties at various length scales ranging through macroscale (> 10 mm), mesoscale (0.1–10 mm), microscale (0.1–100 µm), and nanoscale (< 100 nm). The application of IBSim for material characterisation is used in three main areas: macroscale topology; homogenisation; and the impact of microscale features. Macroscale investigations provide researchers with bulk material properties (e.g., stress–strain relationships, effective Young’s modulus, Poisson’s ratio, plastic strength). In cellular materials, such as foams, the cell morphology (e.g., cell size & shape) and topology (e.g., type of cell such as open/closed wall cells or cell connectivity) can be characterised at the mesoscale [18]. Image-based numerical models of heterogenous materials (cellular materials, multiphase rocks, asphalt, fibre reinforced composites etc.) at the mesoscale can be utilised for homogenisation of material properties by using unit cells or representative volume elements (RVE). The resultant material properties are subsequently used as input for macroscale numerical simulations of larger parts or components. Through analysis of μCT images of porous media, such as rocks, it is possible to measure microstructural features (e.g., pore-size distributions, network connectivity, micro-cracking).

One image-based approach is to gather this type of statistical data about a material’s microstructure which is then used in an analytical method to predict its macroscopic response. For this to be a robust approach, it requires collecting a statistically significant amount of data and thus provides the response which can be expected on average. The direct conversion of microscale images into simulations makes predictions with improved accuracy about the specific part which has been imaged [19]. The limitation in the direct conversion approach is that, due to results being part-specific, a new model is required for each part.

#### Deformation, Damage, and Fracture Performance of Materials

##### Heterogenous Composite Materials

**Fig. 5:**
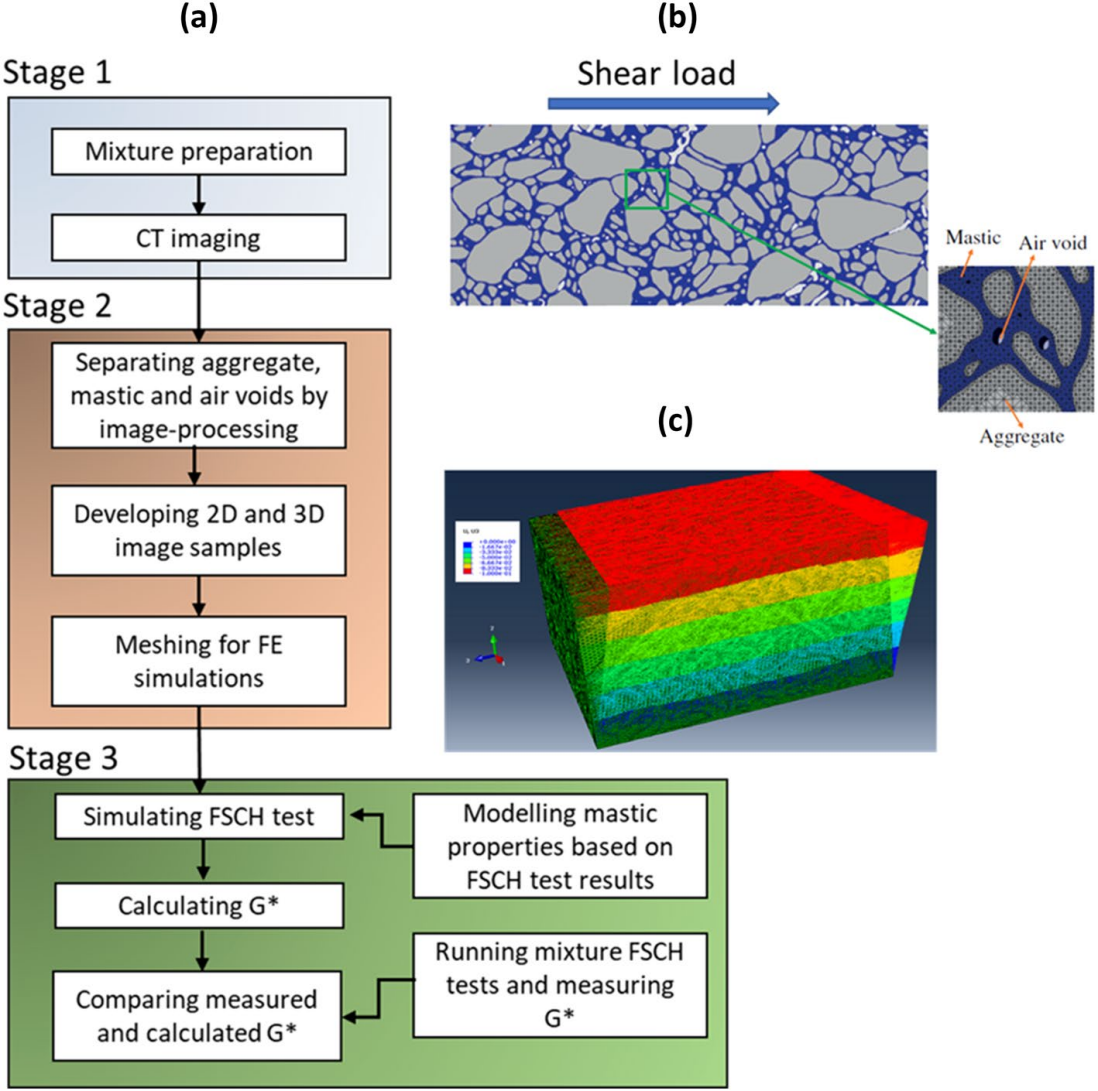
**a** A methodology for the development of micromechanical model of asphalt mixtures and simulations (redrawn from [20]); **b** 2D image of asphalt mixture before FE-meshing [20]; **c** FEA simulation of image-based heterogenous asphalt mixture under shear load [20]

IBSim can be used for material characterisation of heterogeneous materials such as asphalt mixtures [20] or concrete [21]. For example, Fig. 5a illustrates a methodology presenting the stages in order to compute shear modulus using image-based 2D and 3D micromechanical FEA models of asphalt mixtures at high operating temperatures. Where the models were compared with experimental results the 3D models were found to be more accurate than their 2D counterparts. Fig. 5b demonstrates a 2D section of the material, where different material phases (aggregate, mastic, and air void) were differentiated by an image-processing method. In Fig. 5c the undeformed and deformed micromechanical model of the asphalt mixture, which was subjected to a horizontal surface shear load, is displayed in 3D. Another example of heterogenous materials is concrete composed of aggregate, cement mortar and pores. A micromechanical FEA model based on CT images of concrete using MATLAB® was presented to account for micro-damage mechanisms [21] and, the same model was used to improve on the limitations of approaches using statistical random aggregate models. Due to the high computational cost associated with a full IBSim model at the smaller scales, a multiscale approach was followed. In another study, the model included a discrete image-based mesoscale region, where the main concentrations of stress were found, and a homogenised macroscopic lattice region for prediction of mesoscopic crack growth in three-point bending of concrete [22]. In another, a different image-based approach was used via a two scale homogenisation method aimed to predict effective elastic properties of high-performance fibre reinforced concrete, where the elastic moduli of each of the constituents was measured by physical micro-indentation tests [23]. The microscopic level homogenisation focussed on the mortar of sand, cement paste and small pores in a range of 10–600 µm, whereas the mesoscopic level homogenisation focussed on a RVE (a 20 mm cube) of fibres and pores in a range larger than 600 µm. In a further IBSim application example in this field [24], mesoscale damage and fracture behaviour of concrete based on in-situ CT images was simulated in tension and compression with continuum damage plasticity, which elucidated crack initiation and propagation in a complex microstructure of aggregate, mortar and initial voids and cracks. Readers interested in further examples of IBSim applications with cement and concrete are directed towards research by Wang et al. [25] and a review on the subject [26].

##### Orthotropic Materials

In addition to directly converting volumetric images into simulation geometries the information about the morphology of materials produced by 2D or 3D image-processing can be used as input data to feed stochastic models. For orthotropic materials, such as fibre reinforced composites or wovens, examples of statistical and mean morphological characteristics are: orientation distribution of fibres; density of compound materials; pore size and its density in foams [27]. Three dimensional images of ceramic matrix textile composites were obtained by synchrotron X-ray µCT to perform statistical analysis of geometrical and spatial features of fibre tows in 3D woven architectures [28].

**Fig. 6:**
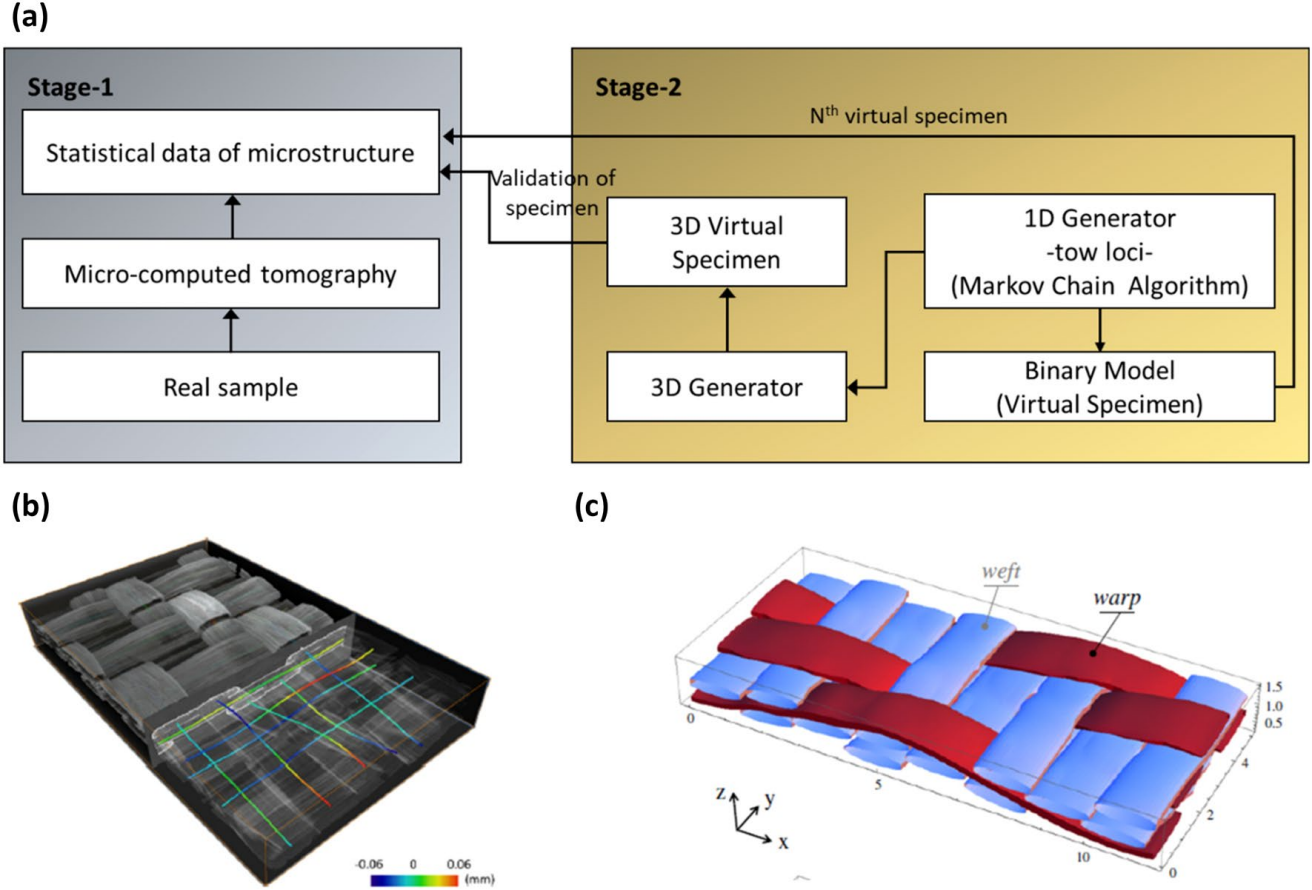
**a** A schematic for generating virtual specimens based on statistical data of real samples (redrawn from [29]) and **b** μCT image of a C/SiC woven composite and the centres-of-mass of tow sections [30]; **c** a 3D rendered virtual specimen [29]

Realistic-virtual textile composite specimens with 3D tows were generated by using the experimental statistical data with deviations [29] and a Monte Carlo based algorithm [30], where textile reinforcements are represented as 1D loci in 3D space. Fig. 6a shows a schematic for generating virtual C/SiC woven composite specimens using statistical description reported in the same source (stage-1) and virtual specimen generator (stage-2). The method on how to compute 1D tow loci based on Markov Chain Algorithm [30] is briefly as follows. First, the extracted 1D tow loci were shown partially embedded in a CT volume in Fig. 6b, then the composites were swept along the tow loci paths to generate the 3D virtual specimens (Fig. 6c). Such realisations based on statistical data of actual samples enable to create as many different FEA models, which fulfil the statistical description, as possible. As noted in Fig. 6c, the computational model demonstrates a homogenised microstructure at fibre scale; however, the fibre tows are represented in a realistic way.

Deformation and damage responses of materials can be simulated at macroscale or mesoscale with Continuum Damage Models (CDM). For instance, Badel et al. analysed woven textile reinforced composites at the mesoscale, where bundles of fibres in yarns were homogenised with an assumption that yarns are transversely isotropic in the direction perpendicular to their fibres [31]. Similarly, fibre reinforced polymer composites were modelled by generating a mesoscale model from XCT data which was coupled to a macroscale model [32].

**Fig. 7:**
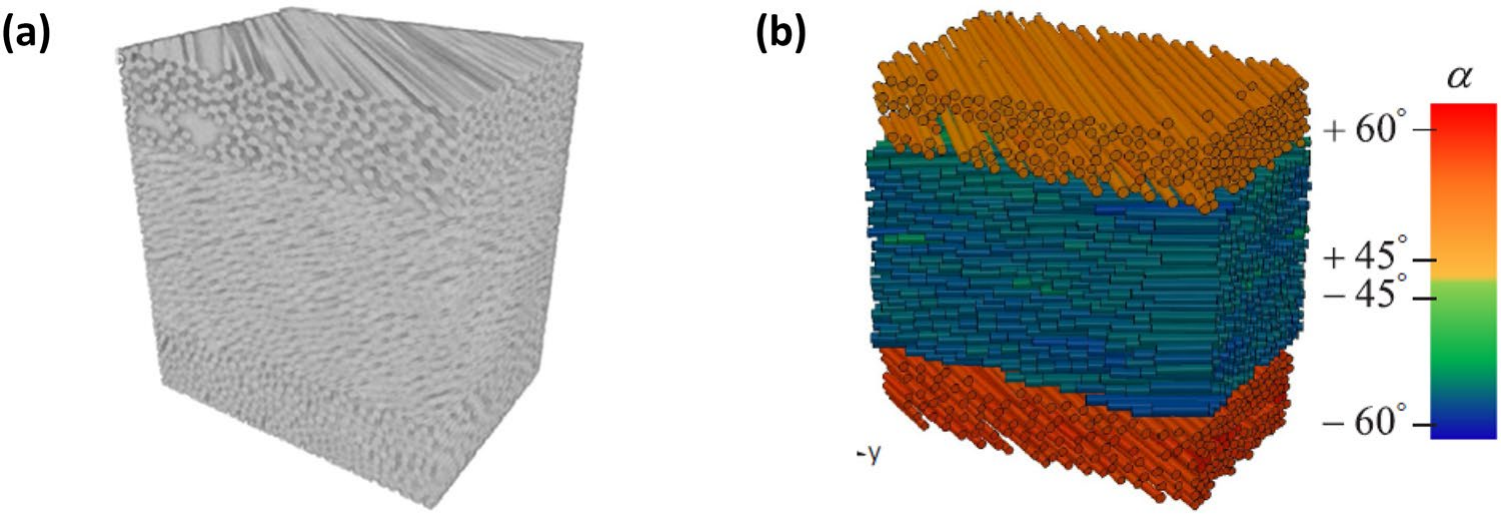
** a** Volumetric rendering from CT data of fibre composites and **b** CAD rendition of their fibres [33]

Constituent fibres can be detected in composite materials by fibre-segmentation algorithms [33], where fibres can be tracked in 3D using a Kalman-filter estimator, for further numerical investigations. Fig. 7a shows the reconstructed volume of a fibre composite and Fig. 7b shows its CAD rendition with orientation distributions generated from the image data. More recently, deep-learning procedures have been used to automate segmentation of 3D CT images from fibre reinforced ceramic composites composed of fibres and matrix in the same material (SiC) [30]. This same study managed to segment matrix cracks in in-situ tensile loading tests with influence of nonuniform spatial fibre distribution.

Ali et al. proposed a methodology to create IBSim FEA models from µCT images of two 2D woven carbon–carbon composites for nuclear applications [34]. These composites consist of multiple phases of the same material. The material properties of the separate phases are required as input data for FEA simulations, in this case the mechanical properties were determined experimentally by physical nano-indentation material characterisation tests. Kishimoto et al. used IBSim to study inhomogeneous local deformation of rubber matrices, where uniformly and non-uniformly distributed silica particles were embedded [35]. By using IBSim models, more accurate results were obtained showing that the inhomogeneous local stress fields strengthened the mechanical properties, such as the ultimate strength, of the material.

##### Additive Manufacturing

Additive manufacturing (AM) is one area that may particularly benefit from IBSim due to the significance of variation between the ‘as designed’ and ‘as manufactured’ states. AM allows highly complex parts to be manufactured, which is one of its main benefits compared to traditional manufacturing approaches, allowing such designs as biomimetic brackets or cellular structures for light weighting advantages. This complex design makes prediction of properties challenging for traditional FEM, especially when manufacturing deviations and flaws can occur.

One of the most important features is porosity within the manufactured material, whether intentional or not, and gaining an understanding of the influence of such pores on the mechanical properties of the material. As an example of a study using IBSim in this context, the tensile deformation mechanisms of porous sintered 316 L steel were investigated with three image-processing approaches [36]. The first two approaches were based on artificially-underestimating the material properties of the material [37] and altering the porosity of IBSim models by changing the greyscale thresholds of shapes to meet experimental stress measurements [38], and the third was a novel approach compensating the effect of CT inaccuracy in porous materials on numerical analysis by modifying μCT images and separating shapes of fissures and small pores. The third approach yielded the most realistic porous microstructures and consequently values in stress distributions when compared with experiments. With this increased level of detail, it was also possible to estimate the critical stress locations where fracture on macroscopic scale was most likely to occur. This method was found to be computationally expensive as well as having issues with convergence. Effective material properties (e.g., Young’s modulus, yield strength, shear modulus and Poisson’s ratio) at the macroscopic scale and local stress & strain distributions were found to be strongly influenced by the image-processing approach applied, with the third approach providing the most accurate results.

Porosity-induced stress concentration on fatigue scatter due to remnant porosity within components manufactured by laser powder bed fusion (L-PBF), an AM method, was analysed with IBSim [39]. The CT scans of the AM components, with a range of pore sizes, were post-processed in a workflow which included VG Studio Max by Volume Graphics®, Simpleware®, and a + CAD® subroutine to generate image-based meshes. Then Abaqus® was used for FEA to compute stress concentration factors using an elasto-plastic material model. The analyses around the pores showed that small pores near to the surface were more detrimental to the material than the pores deeper within the components. In a further example, deformation and damage behaviour of tin (Sn) solder alloys were simulated with image-based FEA models reflecting the exact geometry of pores in solder joints, and the ductile damage mechanisms (crack nucleation and propagation) were described with a damage model to a degree of accuracy not previously possible [40]. The use of ‘stitching tomography’ enabled Amani et al. to increase their detector’s field of view and thus image greater volumes whilst retaining resolution [41]. When coupled with IBSim, this allowed them to analyse the compressive response of AM 3D lattice structures on both the macroscale, i.e., global lattice structure, and microscale, in which micropores and imperfections in struts were captured. Damage and fracture behaviours of the ductile struts were homogenised by implementing Gurson-Tvergaard-Needleman (GTN) damage-coupled plasticity, informing the accumulative porosity computed from high-resolution CT. This presents a very accurate solution to this highly nonlinear multiscale problem and the predicted fracture locations were in a good agreement with experimental investigations. In a similar study, the same two-scale modelling approach and microstructure-informed GTN plasticity model was also practised for open-cell aluminium foams subjected to tensile loading [42].

For full size components the length scale of interest is usually the macroscale: it has been demonstrated that effective material properties such as time-dependent or independent elastoplastic parameters (stress–strain relations), plastic strength (collapse stress) on this scale can be obtained through IBSim. For instance, damage evolution of L-PBF -Printed AlSi10Mg alloys was simulated with CAD-based (as-designed) and image-based (as-manufactured) FEA models of tensile specimens, directly extracted from µCT images by using Avizo®, in order to assess the role of imperfections on mechanical properties [43]. The as-manufactured FEA model met the expectations better in comparison to as-designed model by predicting the higher failure strain due to the geometrical defects present in the parts.

A strong growth area in AM, especially in L-PBF, is the use of in situ monitoring. This refers to imaging of the melt pool and/or the entire build area with optical and infrared (IR) cameras. The presence of defects is highlighted in this way directly when they occur in the layer-by-layer process. It is possible to generate full 3D model data from this in-situ generated imaging data, that could be used in the same way as XCT data for further simulation [44, 45].

##### Foams

μCT-based FEA models of zirconia foams were developed to correlate its macroscopic mechanical response to microscopic features such as thickness of cell struts (i.e., walls), strut waviness and material properties of struts [46]. To do this, the local elastoplastic properties were obtained at strut level with physical micro-indentation tests and plastic deformation was implemented with an isotropic plastic model (Von Mises yield criteria). With a similar motivation, physical nano-indentation tests were carried out on stainless steel walls of cellular materials to obtain material properties as inputs to IBSim models [47]. How to collect material property data appropriate to the scale in question is a major outstanding question in the field of IBSim. This is because of the transition from a homogenised continuum approach at the macroscale to a more granular one on the meso to nano scales to a fully discretised one at the atomistic scale.

**Fig. 8:**
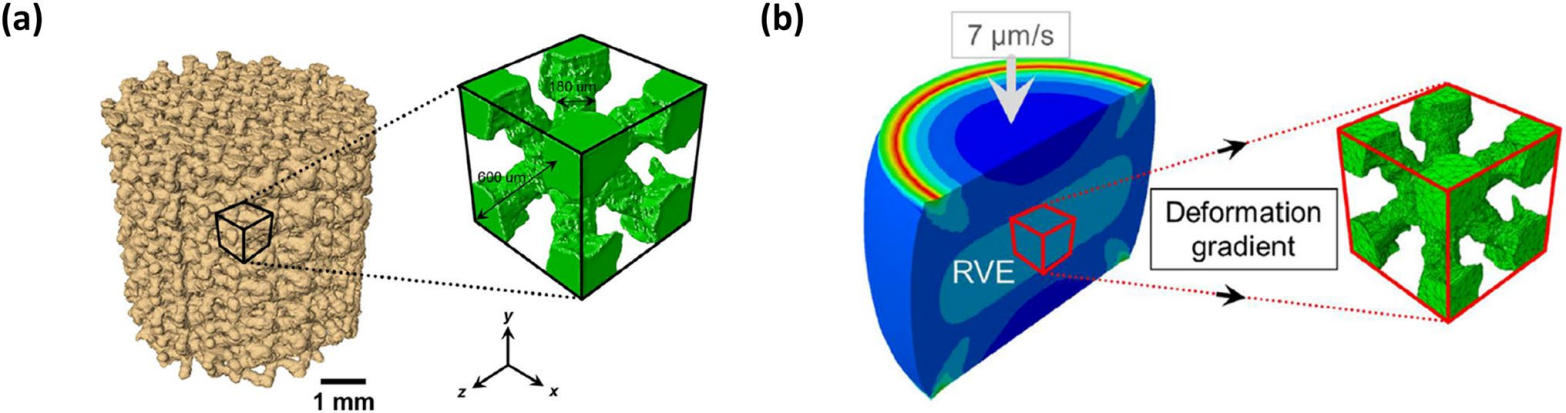
** a** 3D CT reconstruction of Ti foam with a unit cell and **b** its homogenised FEA model under compression applied to the unit cell [48]

Cho et al. conducted a multiscale FEA analyses on titanium (Ti) foams with periodic architecture, the details of which were obtained with µCT images [48] (see Fig. 8a). Virtual uniaxial compression tests on foam specimens (macroscale investigation) were simulated with FEA and the computed local deformation gradients were imposed on the boundaries of periodic unit cells of Ti foam (microscale investigation), illustrated in Fig. 8b. The computational expense of this complex mechanical problem decreased significantly by using this multiscale approach, despite that the microstructural inhomogeneities were included in the FEA model which made improved its accuracy over CAD-based models.

**Fig. 9:**
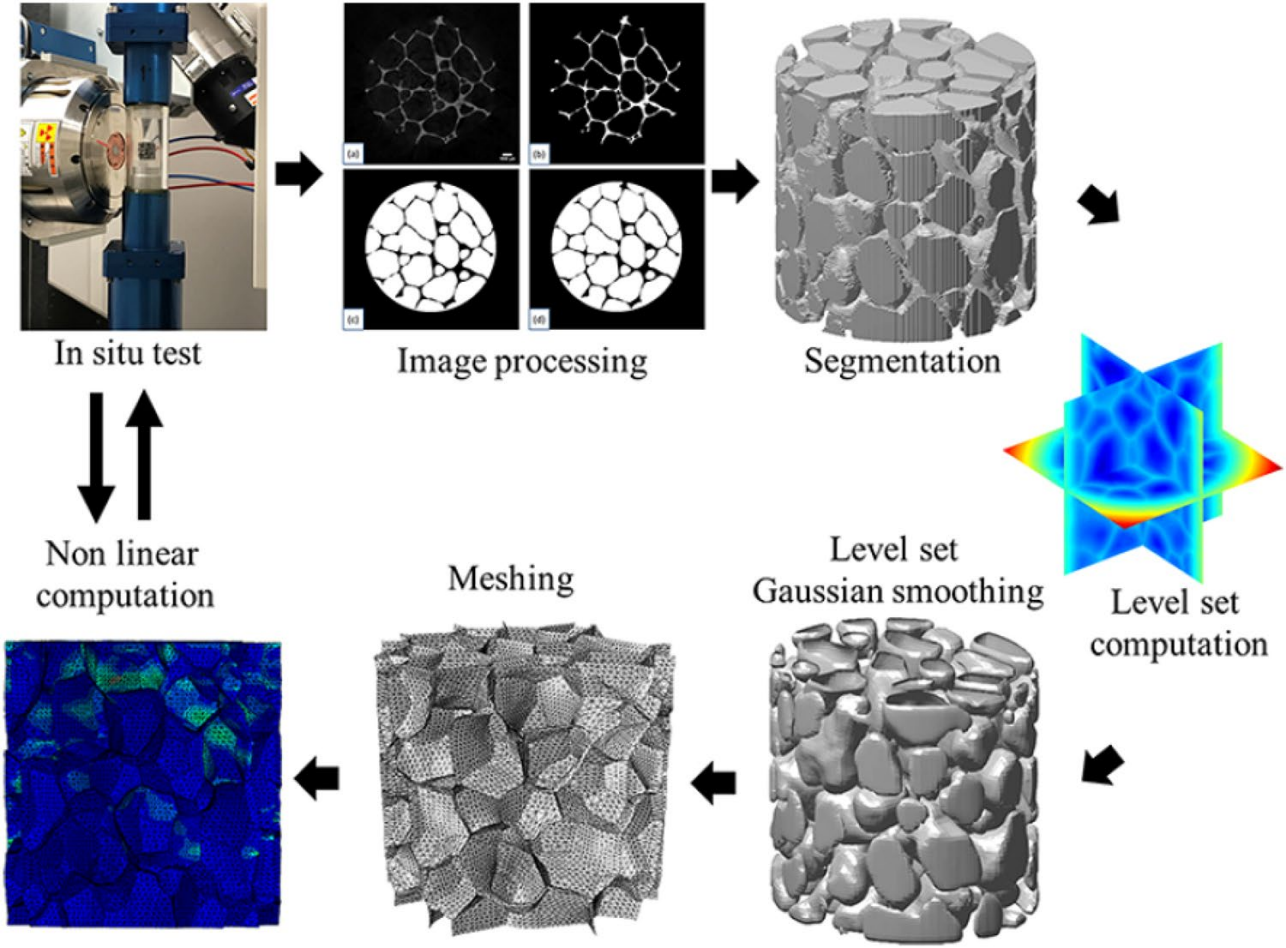
A schematic of CT-based experimental and numerical investigation of closed-cell metallic foams [49]

X-ray-based FEA models of closed-cell metallic foams were compressed under large deformation with nonlinear elastoplastic material behaviour of foam walls, where a watershed method and geodesic reconstruction were used for isolation of cells and identification of missing walls [49]. The steps of this investigation are presented in Fig. 9, where the microstructural deformation and damage patterns of IBSim models were compared to CT images collected in-situ during physical experiments. The collapsing cell-zones in the IBSim models accurately matched that of the physical experiments. The numerical model reproduced the experimental plastic band well in addition to capturing the buckling, bending and fracture behaviour of the cell walls. Comparable research reported the simulation of deformation and plastic collapse mechanisms in closed-cell aluminium foams with contact interaction [50]. Whereas, damage and fracture behaviour of Cordierite-mullite-alumina ceramic foams were simulated with CT-based FEA models and used to compute Young’s modulus and plastic collapse stress [51]. Once more, it was found that the accuracy of the IBSim FEA modelling approach is related to the resolution of the X-ray images used.

Veyhl et al. computed the effective mechanical properties (elasticity moduli and yield strength) of an open-cell porous sponge with porosity of 91–93% and closed-cell foam with porosity of 80–86% by using µCT-based FEA models in the commercial software MSC Marc® (MSC Software Corporation USA) [52]. The elastoplastic behaviour of wall material of cells was modelled by von Mises yield criterion, the anisotropic material behaviour in orthogonal directions was captured by simulations of uniaxial compression tests. Effective strains and stresses were computed from total forces and geometric stretches over the loading planes, which is known as RVE-based homogenisation of material properties.

##### Random Fibre Networks

**Fig. 10:**
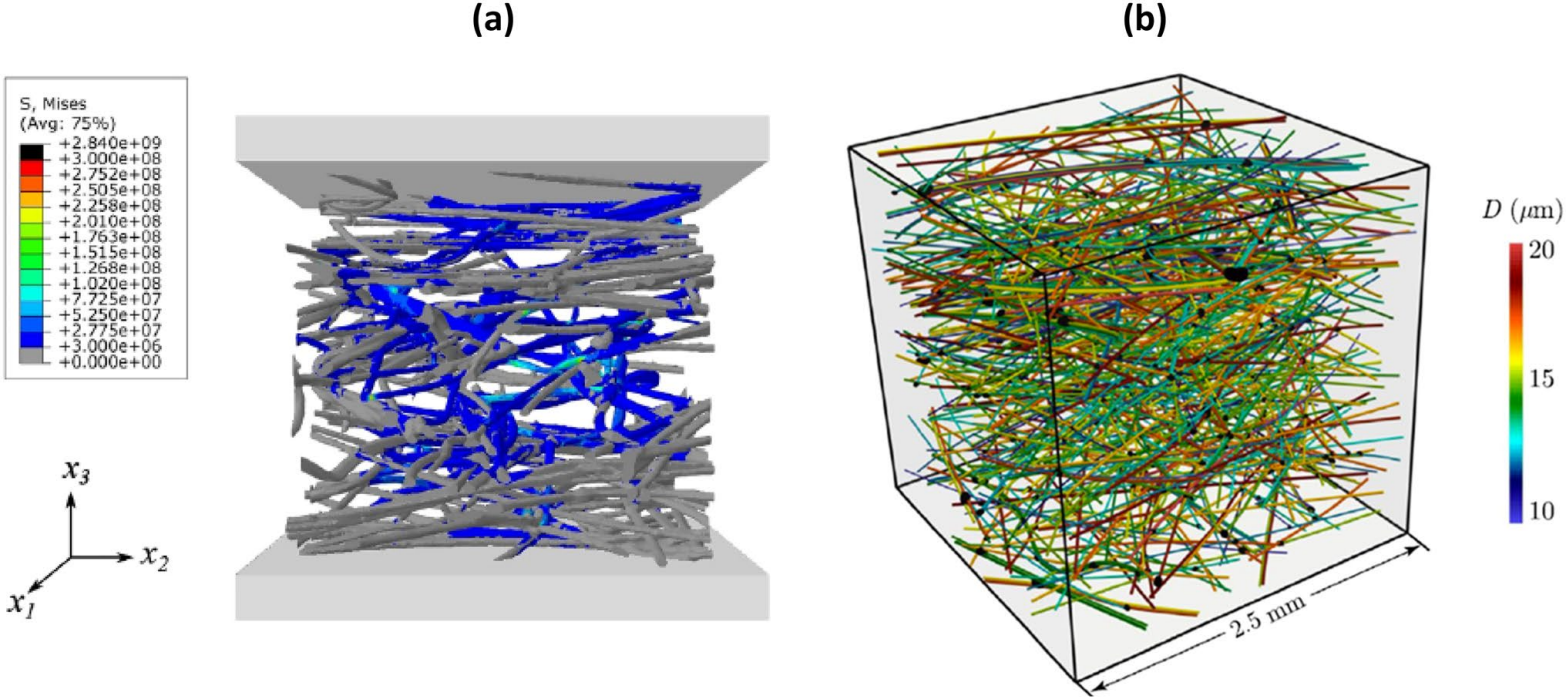
**a** CT-based FEA model of nonwoven solid under compression [55] and **b** a statistical realisation (FEA) model of fibre-glass pack [59]

One of the well-known heterogeneous porous materials, to which IBSim is well suited, is nonwovens. They are composed of randomly distributed fibres, where fibres form contacts between each other. Understanding of their mechanical behaviours and predicting their effective properties are cumbersome because of their complex microstructures and randomness. Therefore, non-destructive characterisation techniques are used to determine their microstructural features such as orientation and length distributions of constituent fibres from 2D or 3D images. For instance, 2D orientation distribution of the fibres was computed from Scanning Electron Microscopy (SEM) or CT images through Hough-transform based algorithms, where fibre edges are detected [53] and this data was later used in another study [54] to simulate tensile and damage behaviour of planar random fibre networks. Some researchers directly processed μCT images of these materials for generation of their computational models [55, 56], one of which is shown in Fig. 10a, and some used such models in their inverse parameter identification studies to find bond strength of fibres [57]. What is more, fibre length and diameter distributions of short fibres were computed from μCT images of wood fibre networks and 3D discrete FEA models were generated by implementing these obtained distributions [58] and similarly this is demonstrated with a 3D stochastic model [59] (see Fig. 10b).

#### Flow and Thermal Performance of Materials

In addition to characterisation of mechanical properties, IBSim is also used to characterise other physical mechanisms. The other main use observed in literature is to study the impact of imaged features on flow and thermal performance. In addition to direct conversion of images into meshes, there are simpler examples in this field which use measurements from volumetric images as input to models which are computationally less expensive. For example, investigating permeability with the resultant pore network extracted from an image can allow the consideration of a larger domain than feasible with full-scale IBSim models if limited by computational expense.

**Fig. 11:**
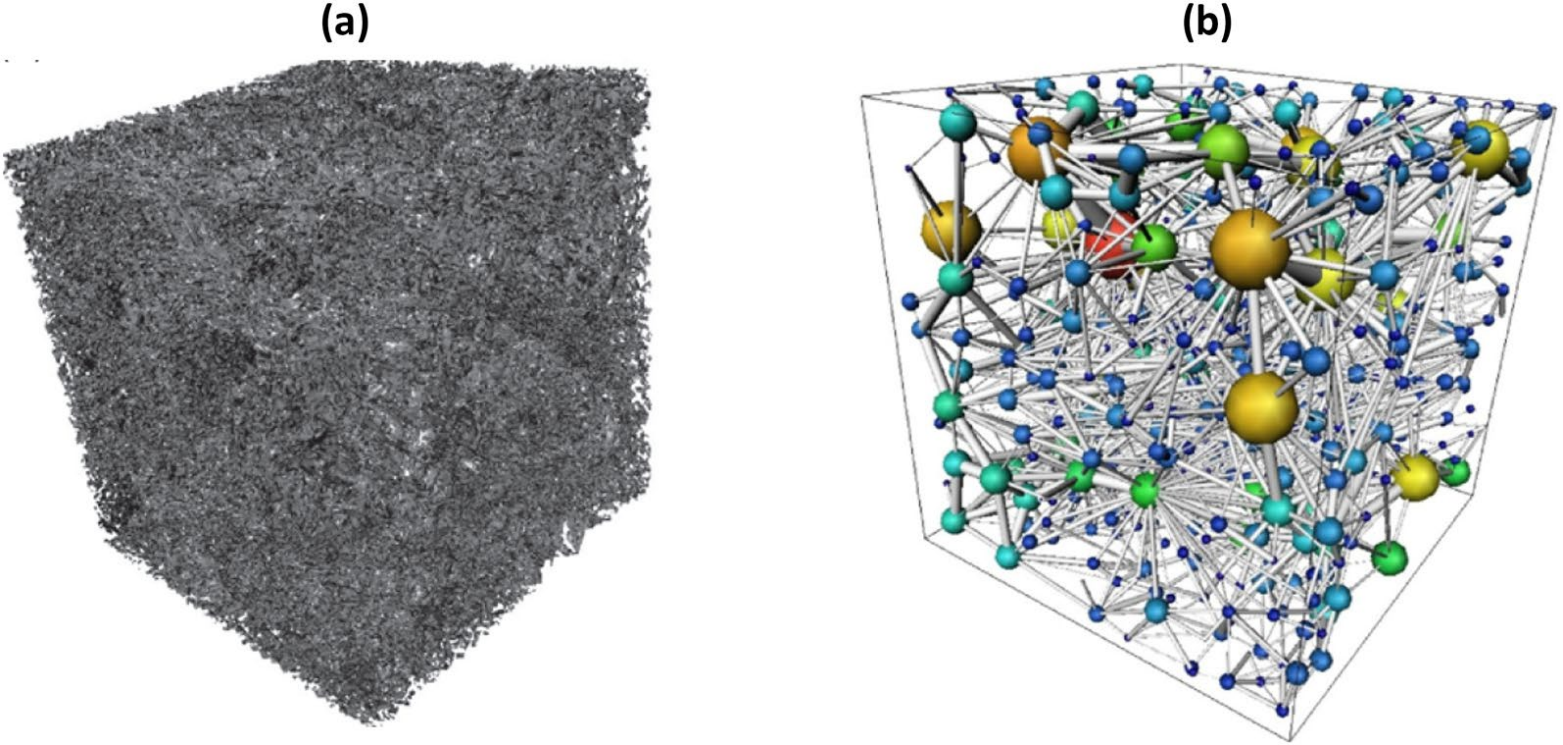
**a** Pore-space image of Mount Gambier; **b** its pore-network model extracted/computed from CT images [60]

Blunt et al. analysed three different porous materials (sand pack, sandstone, and carbonate—Portland limestone) by using X-ray images to extract their pore-scale models and solved those with the Stoke equations governing flow behaviour [60]. An example of (a) pore-space and (b) pore network models of one of the porous materials from that study is shown in Fig. 11. A pore-scale network model was used in order to determine macroscopic transport properties and porosity–permeability evolution during reactive transport processes in a sample reservoir [61]. Bultreys et al. discussed well-known methods to extract pore-scale networks and numerical methods (e.g., traditional CFD, Lattice Boltzmann Methods (LBM), Smoothed Particle Hydrodynamics) to solve Navier-Stoke’s equations [19]. A review of pore network modelling for porous media [62] explored pore network construction approaches and their applications (e.g., adsorption, dissolution and precipitation). Single and two-phase flow behaviour of rock samples were simulated with their pore-network and unstructured meshed models for prediction of permeability under different wetting conditions [63].

**Fig. 12:**
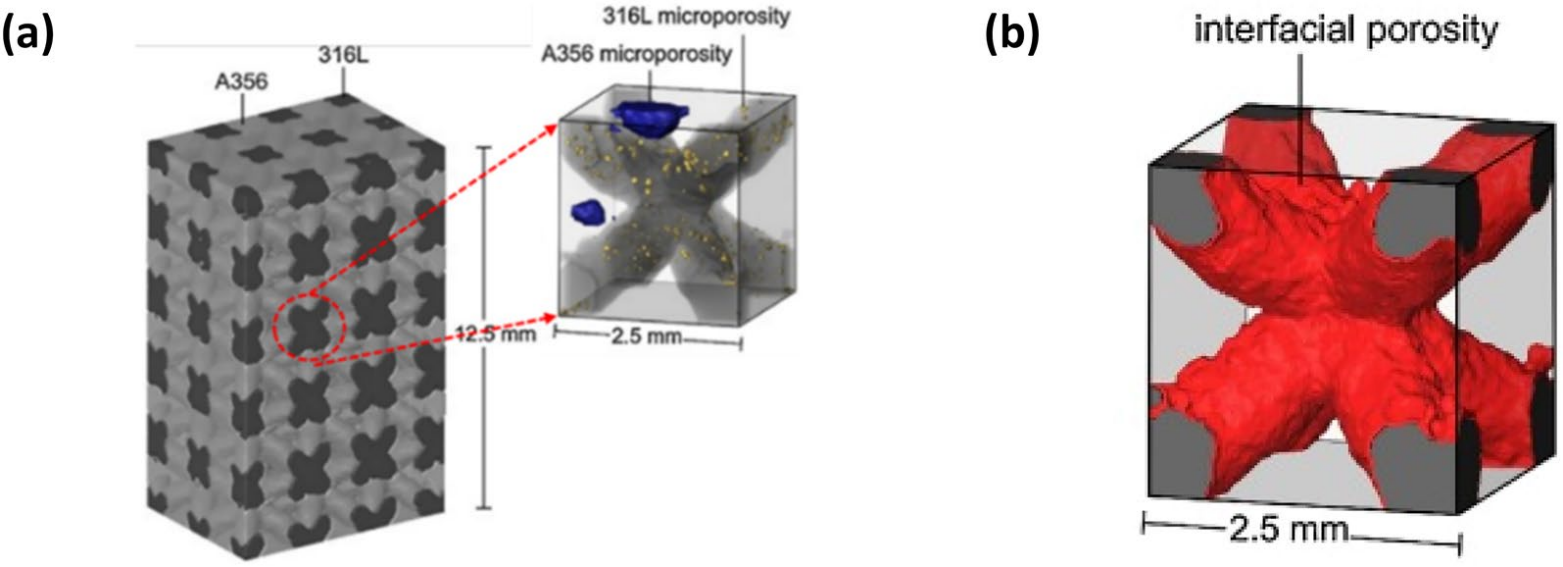
** a** AM A356/316L composite in low resolution and its unit cell in high resolution with microporosities (316L in dark and A356 in bright contrast) and **b** the unit cell with interfacial porosity in high resolution [65]

These void statistics also help to improve the accuracy of micro-mechanics-based constitutive models predicting deformation, damage, and fracture behaviour of materials. For instance, Lu and Chan quantified the three dimensional micro-voids in warm-forging of biocompatible alloys (stainless steel 316 L (SS316L) and a titanium alloy Ti6Al4V) by analysing reconstructed volumes from µCT images [64]. The spatial distribution and number of micro-voids, porosity was obtained through an advanced segmentation algorithm in a commercial software VGStudio MAX 2.2®. AM interpenetrating phase composites were characterised by μCT to detect pores in constituent materials (see Fig. 12a) and their interfacial porosity (Fig. 12b) for the prediction of thermal conductivity [65]. Periodic homogenisation theory was implemented to compute the effects of porosity and unit cell structure on the effective thermal conductivity with the COMSOL® Multiphysics software package.

Geometry and connectivity of pores are a dominant feature of what controls transport properties of porous mediums. Due to this, pore and throat size distributions of Fontainebleau sandstones were measured using synchrotron XCT images in an earlier work by Lindquist and Venkatarangan [66]. Silin and Patzek introduced an algorithm to analyse the geometry and connectivity of the pore space morphology of sedimentary rock, where pore space and throats are distinguished by describing them as inscribed spheres [67]. This work was extended by Dong and Blunt to extract pore-network connectivity out of voxel-based models, constructed from 3D X-ray images, for predicting permeability of porous medium that depends on pore geometries and wettability [68]. In similar investigations for significantly different materials and applications, pore characteristics were computed from the X-ray-based computational models of: bone substitute materials [69], because bone formation over a scaffold strongly depends on pore configurations; proton exchange membrane fuel cells [70] to understand and model two phase flows in a gas diffusion layer; microporous soils, sand-bentonite mixtures, and precision glass beads for testing different segmentation methods [71]; various soil samples for quantification of pore size distribution [72]; soils in the Antaibao Opencast Coal-mine for distribution characteristics of the reconstructed soil [73].

Houston et al. reviewed existing methods in literature estimating pore size distribution and analysed artificial 3D images and actual CT images of various selections of soils in order to make a comparison of their performance [72]. In general, according to Xiong et al., the methods aiming to extract pores and their connecting throats in the reconstructed geometries were listed as (i) statistical reconstructions; (ii) grain-based models; (iii) direct mapping models; (iv) regular network models; (v) two-scale pore network models [62]. Elsewhere, the IBSim approach made it possible to assess pore characteristics (e.g., porosity, pore size distribution, throat size distribution) before/after deformation and damage, and the effect of microstructural changes in porous medium on flow characteristics such as pressure-drop and permeability [74].

**Fig. 13:**
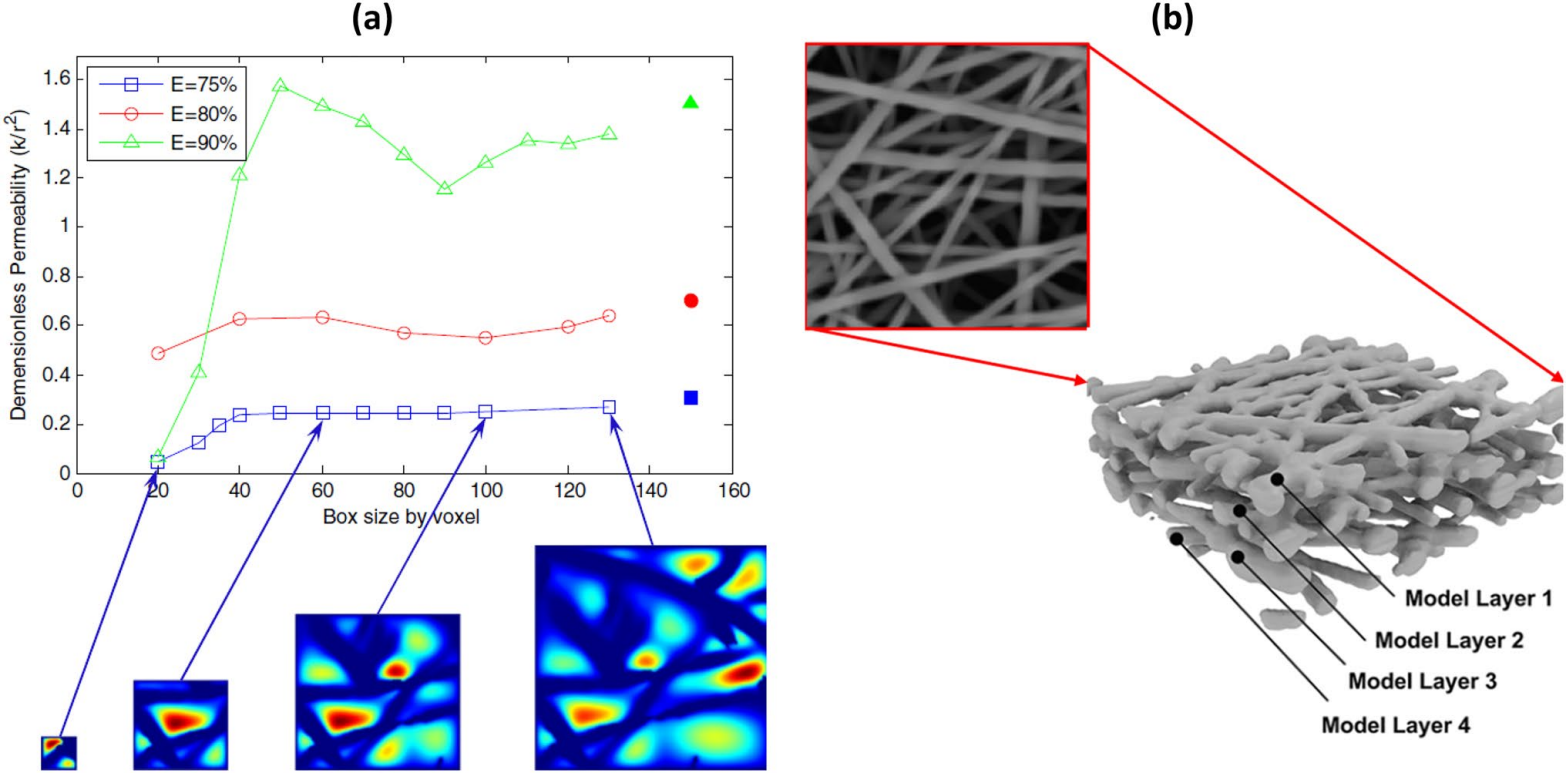
**a** Effect of RVE size on flow permeability for three different porosity levels (E) [75]; **b** SEM image of nanofibre network and its 3D model [78]

As flow permeability, which depends on porous microstructure, is of great interest in industrial applications, morphological statistics of porosity, tortuosity and pore diameters of fibrous media were obtained from high-resolution XCT for use with LBM simulations [75]. These were conducted over various sizes of RVE’s to compute macroscopic transport properties. It is known that the combination of LBM and XCT have previously been used for simulating water flow and chemical transport of porous materials at the pore-scale [76]. Fig. 13a demonstrates the effect of RVE size (or window size) on permeability. Likewise, Kok solved mass transfer equations at low Reynolds numbers for image-based flow models of various fibrous media with a variety of anisotropic fibre distributions (namely, carbon felts and two different electrospun carbon networks used in flow electrodes) by using LBM [77]. Some researchers directly processed SEM images to obtain their 3D computational models, where the filtration performance of polyurethane nanofibre filters was investigated (see Fig. 13b for an example SEM image and inferred 3D layered model) [78]. Saturated fluid flow in packed particle beds [79] was simulated by implementation of LBM in order to calculate permeability from μCT images. Porous gas diffusion layers (GDLs) are key parts of hydrogen fuel cells and, in order to mimic water flow behaviour of the GDLs a pressure drop was applied to one surface of a µCT-based LBM model to simulate the formation of water droplets in the porous microstructure to represent water–gas surface tension [80]. In an alternative example, Navier–Stokes and convection–diffusion equations were solved with the Modified-moving particle semi-implicit (MMPS) method for unsteady and steady-state flow in a disordered porous media [81]. More recently, the effectivity of face masks to filter airborne viruses such as COVID-19 has been of great interest and has also been investigated with IBSim [82, 83].

Water distribution in the hydrophobic microporous layer (MPL) of polymer electrolyte membrane (PEM) fuel cells was computed from image-based pore geometries and oxygen transport mechanisms was simulated through pore-scale modelling, where the simulated oxygen concentration and flux values were averaged to the effective diffusion coefficients of RVEs [84]. Convective drying process, a form of moisture removal mechanism in porous materials, of porous asphalt was investigated by CFD simulations of 3D IBSim models with different airflow speeds and Steady Reynolds-Averaged Navier–Stokes (RANS) k-ε model accounting for turbulent flow behaviour [85]. Flow behaviour in highly porous monolithic alumina columns [13] was simulated by direct CFD models whose porous structure was obtained from 3D CT and the governing flow equations were solved with an open-source CFD tool (OpenFoam) in order to enhance monolith performance.

**Fig. 14:**
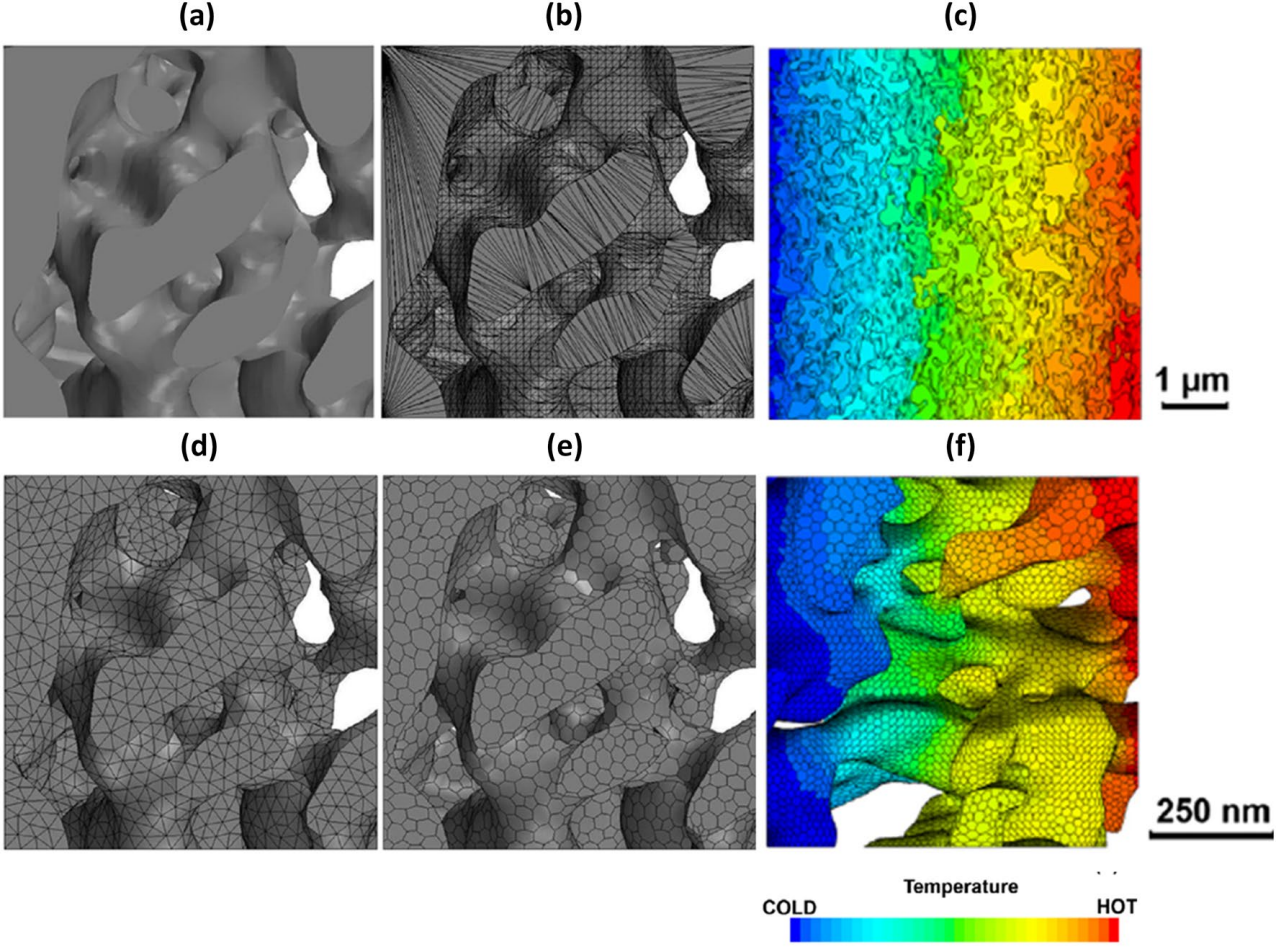
** a** initial surface; **b** surface after Boolean subtraction operation; **d** re-meshed surface with triangular elements; **e** polyhedral volume mesh of porous structure of electrodes; **c**, **f** heat transfer analyses over different domain volume size of LiFePO4 electrodes [86]

Cooper et al. performed heat transfer analysis with IBSim of LiFePO4 electrodes using the finite-volume method in Star CCM + ® [86]. After reconstructing the 3D volume from CT data, they converted the heterogenous microstructure into surfaces (Standard Tessellation Language (STL) format). The file was imported into a CFD pre-processing module in order to volume-mesh the electrode material (first re-meshing the surface and then volume meshing). The workflow is shown pictorially in Fig. 14a, b and d, e with Fig. 14c, f showing the temperature distribution in relatively large and small domain models.

Anisotropic thermal conductivity of a sintered metallic fibre structure with varying porosity was investigated using µCT-based FEA models [87]. It was shown that the thermal conductivity is a function of porosity and fibre orientations. Carbon fibre networks are effective insulators for applications, where the materials are exposed to high temperatures [88]. The geometrically accurate flow models of these networks, digitised from μCT images, were used to calculate temperature-dependent permeabilities. Similarly, room temperature conductivity of carbon fibre networks was computed with voxel-based IBSim models, where oxidation behaviour and surface reactions were incorporated into microscale simulations [89].

Effective anisotropic thermal conductivity of a glasswool insulation material composed of randomly distributed fibres, the main source of anisotropy, was characterised by FEA simulations generated from X-ray images by solving 3D heat equations and applying different temperature distributions over surface boundaries in order to create and measure temperature drops [90]. The thermal conductivity of highly porous metal foams was analysed with IBSim FEA models and it was numerically proven that the RVE and FEA element size are two parameters which have a non-negligible impact on the virtually measured thermal conductivity, thus highlighting the importance of performing sensitivity analyses as part of the methodology [91].

Electrochemical performance of carbon felt electrodes of redox flow batteries is influenced by the microstructure of carbon felt. CFD simulations were used to investigate compression of the felts and thus predict the increase in pressure drop due to microstructural changes [92]. High-resolution µCT CFD simulations of open-cell aluminium foams with different pore densities, i.e., number of pores per unit volume, were conducted to predict permeability and effective thermal conductivity under incompressible flow and steady state flow conditions [93].

**Fig. 15:**
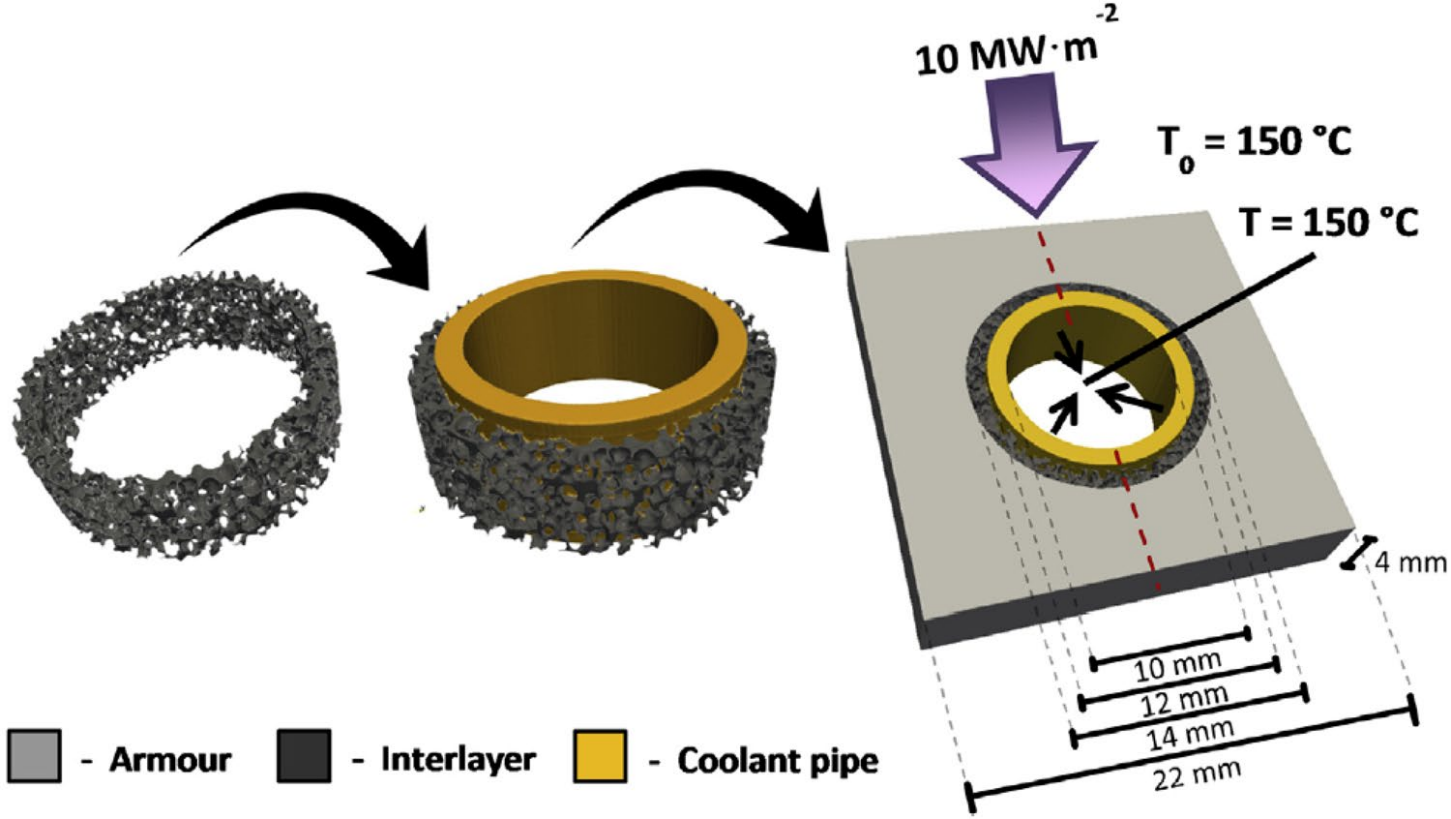
Virtual manufacturing workflow from graphite foam interlayer to CAD pipe and CAD armour with thermal boundary conditions [94]

Evans et al. carried out a thermal analysis of a heat exchanger component (Fusion Energy Monoblock) by using a hybrid FEA model containing: a graphite foam interlayer with microscale accuracy directly derived from CT images; a CAD-based armour and coolant pipe [94] (see Fig. 15). The graphite foam ring layer was digitally ‘cut’ from a larger block of imaged material, thus being able to rapidly assess the design without the need for physical manufacturing. In another study, the thermal response of a carbon fibre composite-copper monoblock was simulated with IBSim FEA [16]. The model included a debonding region at the carbon fibre composite-copper interface. By capturing the debonding at this interface, this ‘as-manufactured’ simulation predicted the loss in thermal conductivity at the interface, which would not have been included in an ‘as-designed’ model. This led to a rise of over 20% in the peak temperatures which consequently would have increased the thermally induced stresses.

#### Multiphysics Performance of Materials

A review of analytical models to predict electrical conductivity in porous media was published by Cai et al. [95]. These analytical modelling approaches such as pore network and percolation modelling rely strongly on the processing of detailed microstructural images.

Commercial use of solid oxide fuel cells is limited by technical issues such as thermal gradients across the cell developed during operation leading to deteriorating the battery performance [96]. Electrode polarisation losses of solid oxide fuel cells, associated with the composition of constituent materials and their microstructure, reduces performance. The underlying electrochemical processes (e.g., oxygen diffusion in gas phase and charge transfer at the interface electrolyte–electrode material) were investigated for a porous mixed ionic-electronic conducting cathode by using 3D FEA models based on reconstruction of focused-ion beam (FIB) serial sectioning and SEM imaging to produce tomographic images (i.e., FIB-SEM) [97]. Furthermore, effective electrical conductivity of composite asphalts with randomly distributed steels in an epoxy was numerically analysed [98]. Also, multiphysics simulations of solid oxide fuel cells based on 3D micro/nano reconstructions were performed by taking conformal boundaries between different phases into account [99].

**Fig. 16:**
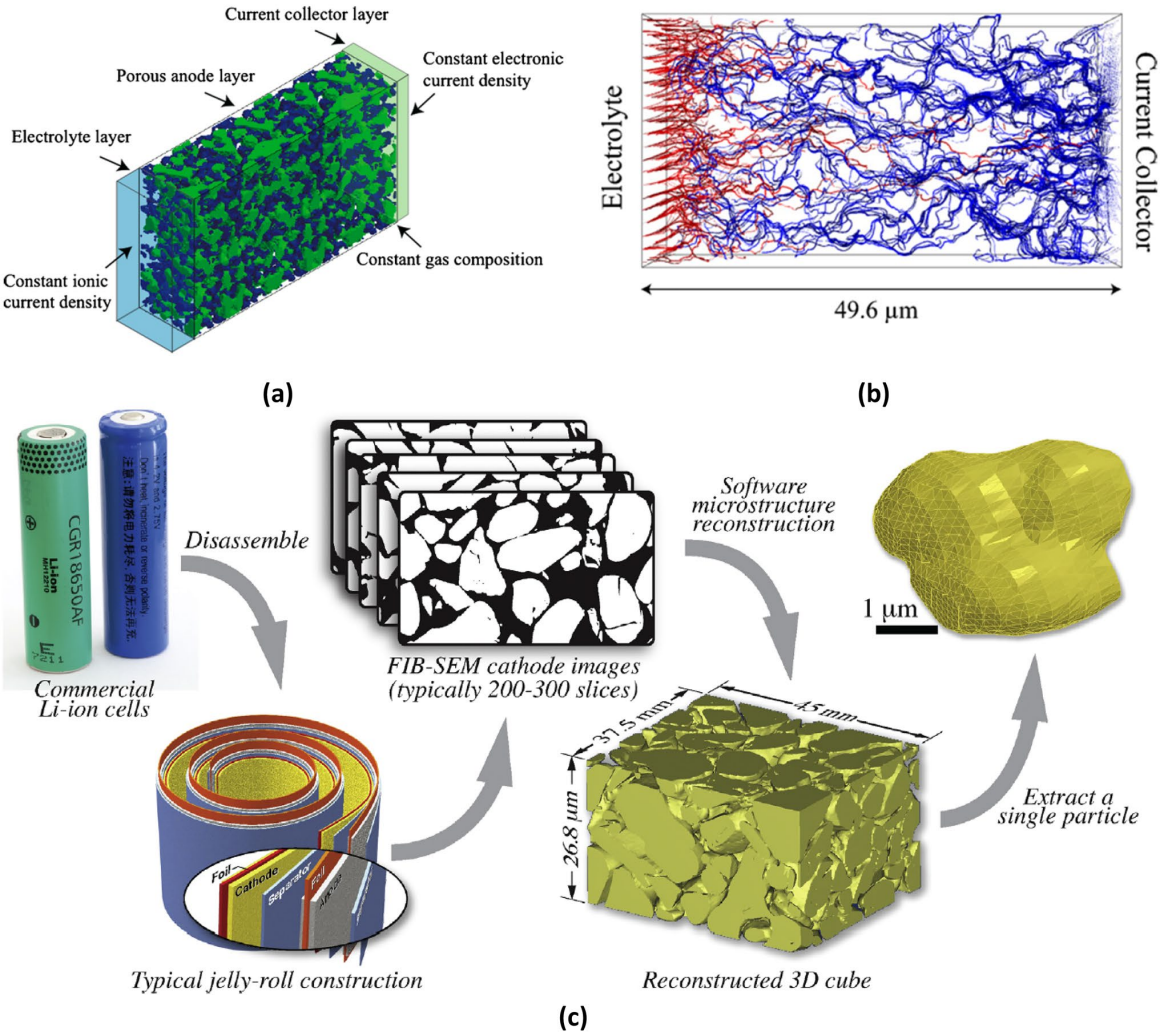
**a** A computational model of anode microstructure with boundary conditions [104]; **b** 3D current stream line distribution (red and blue colours are ionic and electronic currents, respectively) [104]; **c** individual complex particles extracted from reconstructed volume of commercial lithium-ion batteries for stress analysis [105]

Zhao et al. performed a comprehensive review on modelling approaches for the coupled chemo-mechanical behaviour of Lithium-ion batteries at particle, electrode and cell levels [100]. In this, the capacity loss in these batteries during charging/discharging cycles were associated with some phenomena such as nonlinear elasticity, plasticity, anisotropic mechanical behaviour and phase separation. Hein’s electrochemical simulations of Lithium-ion batteries relied on CT-based parametrised stochastic models and non-parametric realisations extracted from reconstructions of CT volumes [101]. Numerical methods such as LBM have been used to compute electrical and species transport properties of lithium-ion batteries in order to develop new products or optimise their performance [102]. Since lithium-ion batteries experience electrode failures due to diffusion-induced stresses occurring in charge and discharge, these processes were simulated by Lim et al. with micro and nano CT-based FEA models of active particles for different discharge rates (C rates) and, the non-uniform/complex shape of the particles increased in induced von Mises and Tresca stresses leading to failure [103]. Fig. 16a shows a microstructural model of lithium-ion battery anode with boundary conditions and Fig. 16b, the 3D distribution of electrical current throughout pore space [104]. The workflow starting from a commercial lithium-ion cells (batteries) down to single particles extracted from reconstructed CT volumes are presented in Fig. 16c. A similar methodology was implemented to predict the transient stress-fields over the cathode particles of commercial lithium-ion batteries by coupling electrochemical processes with mechanical ones [105]. The swelling in LiCoO2 cathodes was studied with coupled electrochemical-mechanical simulations to unfold the mechanisms of stress generation and the effect of process parameters along with microstructure on these stresses [106]. Galvanostatic discharge processes of LiCoO2 cathodes at various C rates were simulated with 3D IBSim models by Yan et al. [107]. A comparison between macro and microscale IBSim models of lithium-ion porous battery electrodes was made in terms of their electrical conductivity and diffusion [108]. Elsewhere, mesoscale multiphysics simulations bringing electrochemistry, mechanical deformation and transport processes together in lithium-ion batteries incorporating conductive binder particles were presented [109–111]. An X-ray-based realistic 3D microstructure numerical model enabled the authors to obtain the stress accumulation in nickel-manganese-cobalt (NMC) half-cell, resulting from the phase transitions and lithium intercalation [112]. Multiscale investigations on Lithium-ion batteries revealed porosity from X-ray microscopy and effective diffusivity as well as tortuosity from computer simulations in GeoDict® [113].

Fluid and electrical flows through reservoir rock samples accommodating highly complex pores were simulated with the COMSOL® multiphysics simulation tool [114]. In this work they conducted a downsampling study, where the sizes of volume elements were controlled. This indicated that, as some pores disappear, and the remaining ones alters geometry, fluid and electrical flow patterns were affected significantly. Together with this, IBSim had been used to model water and oil distribution formations in the microstructure of a porous rock and to investigate the effect of rock wettability on electrical properties [115]. An IBSim approach enabled the authors to examine the effect of calcite precipitation on the permeability of a porous media with a Stokes solver (an inhouse solver implemented in Avizo® under XLabHydro®), where the precipitated particles and porous media microstructure were captured by µCT and the pore network was converted into a flow model [116]. Another open-source software package for porous materials is PuMA (Porous Microstructure Analysis), computing effective material properties such as thermal and electrical conductivities by using finite difference Laplace solvers [117]. An effective thermal conductivity of a composite material with anisotropic constitutive phases was predicted with PuMA [118]. The software package can be used for virtually generating a computational domain of arbitrary porous structures and the tortuosity of these artificial models or their 3D IBSim models can be computed with a random walk algorithm [119]. The package was integrated into an image analysis software Dragonfly® (Object Research Systems, Canada).

### Characterisation of Manufacturing Processes

#### Defects and Manufacturing Process Errors

**Fig. 17:**
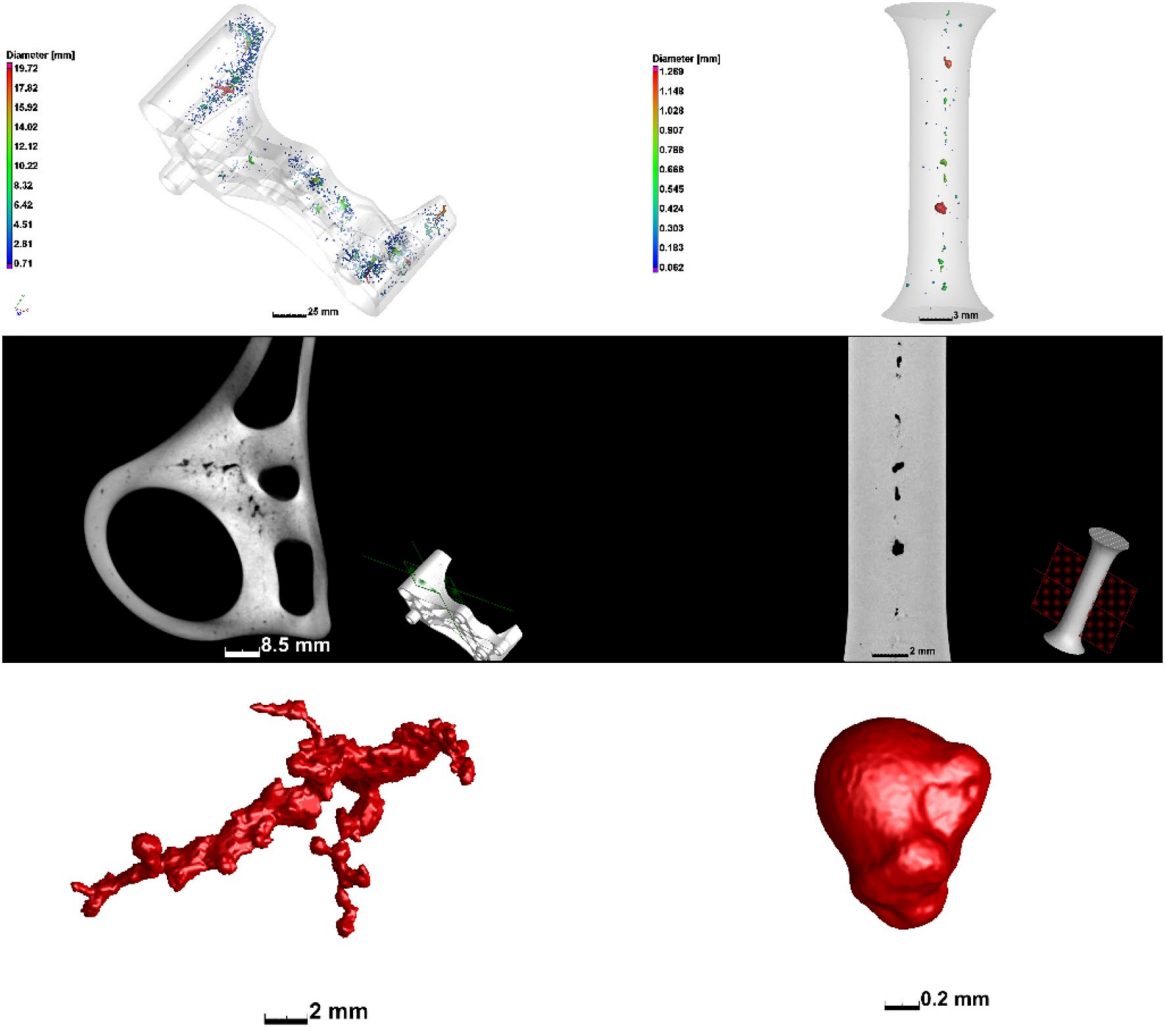
Examples of casting porosity including (left) shrinkage porosity and (right) gas porosity, image from [120]. The samples are a commercial sand-cast aluminium alloy automotive part (left) and investment cast titanium alloy machined to a tensile dogbone geometry [145]

Different manufacturing processes have different unique defect types inherent to the process which may occur, and which require attention to minimise their extent in manufactured products—i.e., optimisation of the processes is often needed. For example, metal casting processes are prone to shrinkage porosity and gas porosity (shown in Fig. 17) which are formed due to the entrapped gas during the casting process (gas porosity) and due to inadequate filling of the casting mould, with subsequent cooling and shrinkage of the molten material (shrinkage porosity) [120, 145]. These can be minimised by varying the casting infill velocity, ingate geometry and location(s) and cooling of the mould. Formation of microporosity in the solidification process of Sn-Bi alloys in a copper mould was investigated with X-ray and FE modelling and the porosity strongly depends on alloy composition [121]. Similar porosity formation occurs in plastic injection moulding processes. These defect types are conventionally detected by NDT methods such as X-ray radiography (2D) or CT (3D), and may be used to improve the manufacturing process or may be used for pass/fail decisions for individual parts [27, 122, 123].

**Fig. 18:**
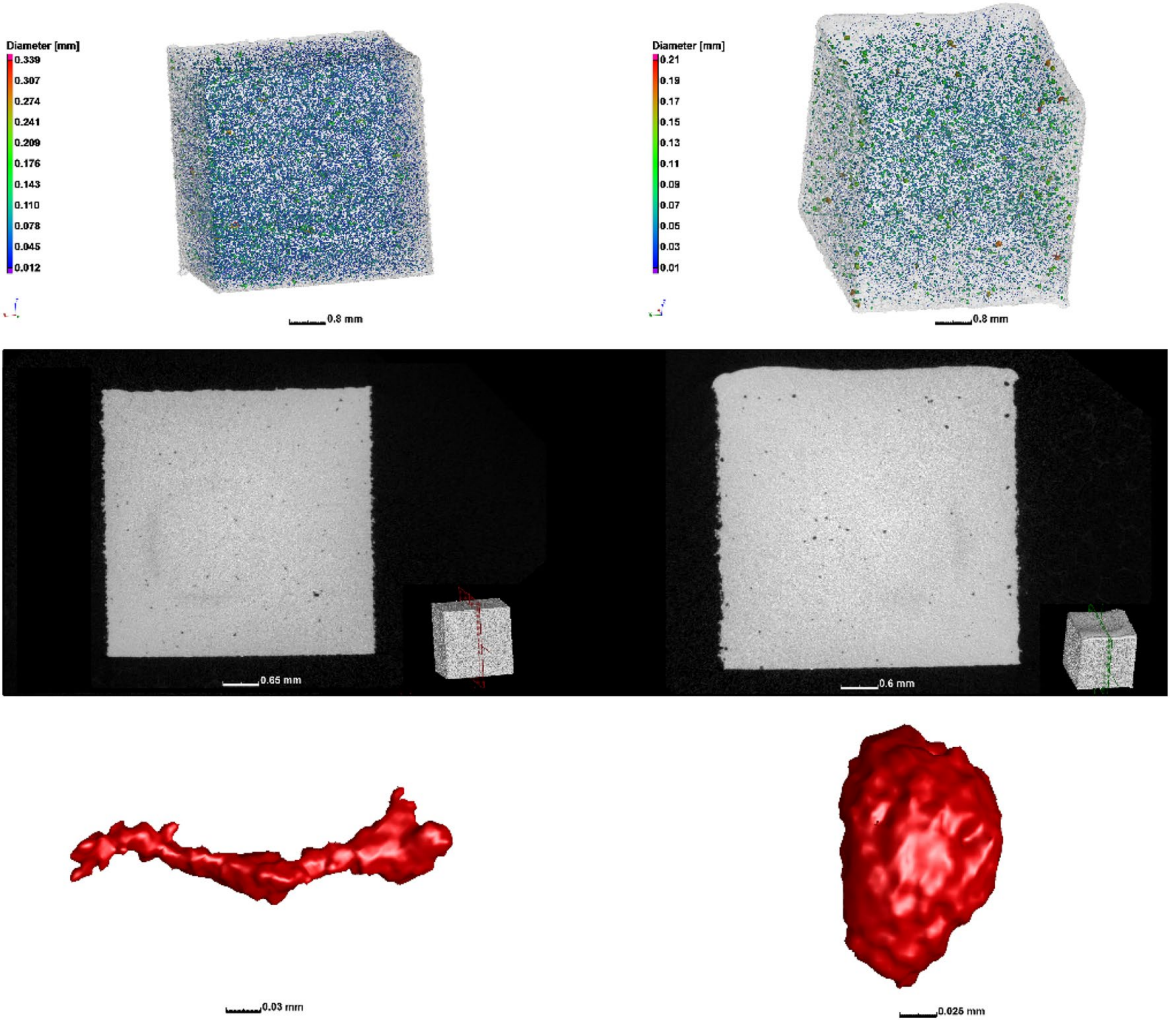
Examples of porosity in metal AM including (left) lack of fusion porosity and (right) keyhole porosity, image from [120]. The samples are small cubes of titanium alloy, manufactured using different process parameters—such cubes are often used to optimise the process allowing up to 99.99% dense parts under optimal conditions [125﻿]

In AM, different types of pores are formed with very small size in comparison to castings and injection mouldings, but with a wider distribution in the part (see Fig. 18) [120]. This is due to the track-by-track and layer-by-layer manufacturing process which creates possibilities for pore formation in smaller regions but more widespread in all regions of the part. A recent review explains defects and anomalies in metal PBF in detail [124]. Another paper focuses on characteristics and variability of defects occurring in metal laser PBF [125].

The above-mentioned examples are illustrative of the types of porosities of different sizes, morphologies and distributions that may occur in various manufacturing processes, and which can be improved by optimising the manufacturing process. Further examples are found in different studies showing the presence of pores in different materials and due to different parameters [126–129]. The intentional variation of manufacturing process parameters shows clearly the influence of each parameter on pore formation [130], and in a recent round robin test, different porosity distributions were found in samples produced in different laboratories [131].

Other defect types that occur in manufacturing in general, besides porosity, are inclusions, cracks, geometrical inaccuracy, surface roughness, residual stress, and microstructural anisotropy or inhomogeneities. All of these defect types are known to influence mechanical performance, either by reduction of yield strength, reduction in ductility, or in lower fatigue strength [132, 133]. They are discussed below briefly in the context of IBSim and the possibility for process optimisation.

Surface roughness, indentations, scratches or surface damage of any kind can influence the mechanical properties especially acting as fatigue crack initiation sites [134]. In this work, FEA simulations of the ideal geometry were used in combination with local surface depression depth, to create a modified stress intensity factor which correlated well with fatigue crack initiation site, despite differences in residual stress and microstructure between samples (due to different build orientations). Similar work was reported for lattice structures manufactured by AM [135, 136]. Further work is needed to make direct simulations utilising the actual surface morphology, as there may be shielding effects where adjacent depressions or pores create stress shielding or may enhance the local stress in some places. This was preliminarily investigated already in 2D [137]. Because achievable CT resolution is limited to sample size, the incorporation of relatively small features in images and subsequent models is challenging. A good example is the surface roughness for a macroscale component—by scanning the whole part the surface roughness details are not included. Small coupon samples may be used in addition to the full-scale part, to provide some inputs, despite its limits (possible variations from larger part and no direct correlation).

Residual stress is another strong influencer of mechanical performance and is difficult to characterise and incorporate into simulation models, because of the challenges in its measurement [138, 139]. Since the characterisation of residual stress is either destructive (by hole drilling or similar methods), or in laboratory instruments only providing limited depth information, no work so far has incorporated the influence of residual stress into simulation models, to the knowledge of the authors. What is often done, however, is to compare predictions of stress based on manufacturing process simulations with stress maps obtained from X-ray diffraction imaging at synchrotron sources [140]. In AM, much effort is made in process optimisation by thermal simulations to predict residual stress and minimise this by simulation and variation of scan strategies [141].

Microstructure of metals (grain sizes, orientations, granular structure, etc.) is similarly challenging to characterise non-destructively and hence difficult to incorporate in IBSim models. Technically this is possible using destructive imaging and correlating microstructural mechanical properties in averaged volumetric regions, or non-destructively using diffraction contrast imaging. However, to the authors’ knowledge, there are no examples where direct incorporation of these approaches with IBSim has been reported yet.

#### Effects of Defects

The influence of porosity on fatigue performance has been reviewed comprehensively [133, 142, 143] and more specifically for metal AM by Sanaei et al. [144]. Irregular-shaped pores (as in Fig. 17a or 18a) are more detrimental to mechanical performance, as are larger pores and those closer to the surface of the part. Cracks (e.g., from manufacturing induced stresses) are similarly detrimental and more so when they are closer to the surface or larger in size (or both).

**Fig. 19:**
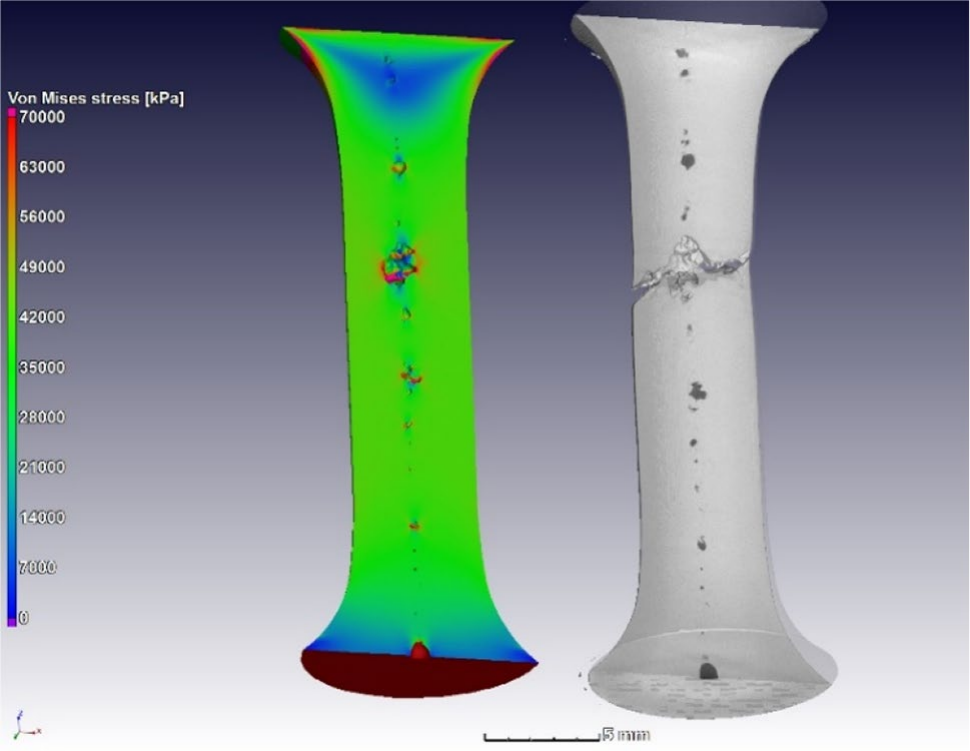
IBSim model before (left) and after (right) tensile testing to failure [145]

The effects of pores on mechanical performance may be investigated by IBSim. When compared directly with a physical test (i.e., performing a simulation of the test on the digital representation of the sample), the physical results may be used to verify the IBSim model which can then be interrogated in greater detail than the results from the physical counterpart, allowing localised measurements through the sample’s full 3D volume with microscale accuracy. For example, the stress distribution around casting pores were evaluated as shown in Fig. 19, before and after tensile testing [145–148]. Similar work was reported for brackets fabricated by AM with pores [149], and for pores in high pressure die castings in recent work [14], and for prediction of mechanical properties in aluminium castings [150, 151], and for mechanical characterisation of AM nickel–chromium alloy samples [152]. A Bayesian-based statistical analysis was conducted for uncertainty quantification of pore distributions in AM components [153], which was later used for developing a probabilistic constitutive damage model. A recent study made use of AM to artificially create defects in tensile samples, and made use of XCT and IBSim to investigate the effects of the defects on tensile behaviour [128]. Similar investigations were reported using artificially induced pores and XCT [154, 155], though these do not include simulation. The influence of pores on fatigue performance is also widely acknowledged, as the pores act as stress concentration locations for crack initiation. This was studied using in-situ synchrotron imaging in castings with pores, finding the exact crack initiation location at pore boundaries and applying IBSim to complement the study [147].

The effect of defects in metal AM was reviewed recently in the context of XCT imaging insights [132], where it is evident that most of the small porosity in these materials influence the ductility of the parts but not the strength, unless present in excessive amounts (> 1%). It has also been found that lack of fusion pores with irregular shapes are especially detrimental to fatigue properties, as are all large pores near the surface [144]. As one can expect, these influences become difficult to predict when the part geometry is complex, and/or when loading scenarios are not simple (e.g., multiaxial loading).

In cases where the geometry or loading scenario is complex, simulation is highly valuable for the performance and property prediction. Examples of highly complex geometries are cellular porous “lattice structures” manufactured by AM [156–158]. IBSim models have been used to compare different ideal geometries of such lattices of different designs, showing differences in permeability and stiffness, factors important for medical implant applications [159]. This allows an improved design choice to be made. Engineering simulation is widely used already to check performance of designs prior to manufacturing, this is even more important with complex geometries becoming possible through AM [160, 161]. It is also possible to incorporate expected defects into such ideal models to predict the effect of manufacturing defects and predict a critical size of such defects, as was done for a pore in the middle of a single strut of a lattice structure [162].

**Fig. 20:**
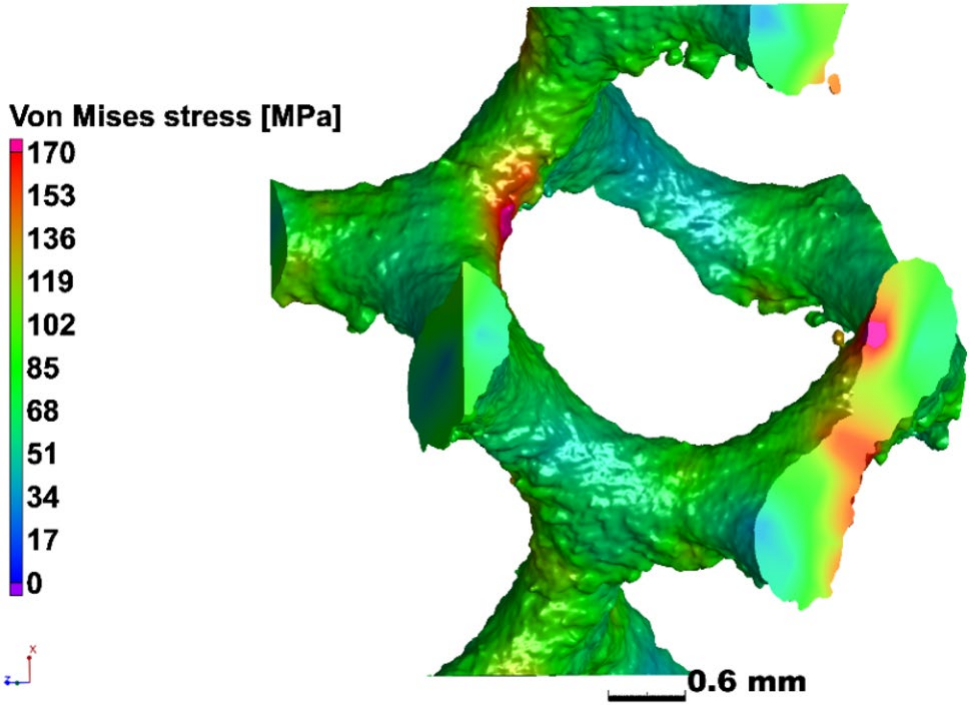
IBSim model of loading applied to a gyroid lattice structure of titanium alloy produced by L-PBF. The small section viewed here is cropped from the larger structure showing the location of high stresses, and the rough surface exacerbates this [163]. Compressive loading is applied in the vertical direction in the image

Despite the capabilities of simulations of idealised “design” models, all manufactured parts inevitably have some geometrical inaccuracies, defects, and deviation from ideal design geometry. Here IBSim of realistic models from μCT or other 3D image data is particularly useful, as the simulation of the actual geometry including its defects and inaccuracies provides insight into the influence of these defects on the performance. One example is shown in Fig. 20 where a load simulation was applied to a gyroid lattice structure manufactured by L-PBF [163], similar to that reported by Plessis et al. [164]. This highlights the locations of highest stress in relation to the local rough surface. AM single lattice struts with process-induced geometrical imperfections were analysed with IBSim and multiscale modelling [165]. The use of IBSim in order to incorporate the influences of porosity and surface roughness into the predicted performance was previously suggested [166], and was used to correlate stress concentrations to failure locations in compression tests [167]. Amani et al. used a similar approach for lattice structures incorporating defects, surface roughness and using a GTN model to include void nucleation and growth into the simulation model [41]. A simplified approach was also used for modelling irregularity in strut diameter to model the realistic manufacturing quality of struts [168] and nodes [169] on mechanical properties of lattice structures. Numerical studies of cellular structures incorporating defects have also been reported [170–172]. Foams and stochastic porous materials have also been the subject of IBSim studies in the past [173].

### Impact of Deviations from Idealised Design Geometry on Product Design and Performance

The simulation of full-scale components in the HVM sector is not new. As already noted, real parts often deviate from idealised design, adding uncertainty to the results obtained from conventional simulations. Consequently, large safety margins are often imposed upon in-service components. Here, IBSim comes to the fore, incorporating various object-specific deviations and unique aspects into the simulation, for better prediction and characterisation. This increases confidence in performance prediction (or reducing uncertainty) and thus allows smaller safety margins to be imposed. When considering issues relating to full-scale components, this refers to deviations in actual part size, warping, surface roughness, micro-cracking, or bonding interfaces. Especially for complex shaped parts, the influence of such defects or deviations might be unpredictable, and hence the need for further quantification by IBSim.

At the macroscopic level a material or group of materials might be chosen for a task based upon idealised macroscopic properties. Important macroscopic metrics may include overall volume and weight, with further considerations of geometric tolerances, dimensions, and surface areas. One can also look at the microscopic aspects of materials, particularly their structural arrangement, and subsequently attempt to design an idealised microstructure, with key metrics such as the arrangement of pores and struts, or the size, shape, and orientation of grains and fibres. For an engineering product, as already discussed, these microscopic metrics play an important role in determining macroscopic behaviour.

Assessing the impact of deviations from idealised design geometry is a multiscale problem, and the methods by which geometries are digitally acquired (e.g., surface, or volumetric scanning) and transferred to the computational domain must, therefore, accurately capture deviations across multiple length scales for IBSim to be representative. This is a complex task as no single method covers all scales of interest.

**Fig. 21:**
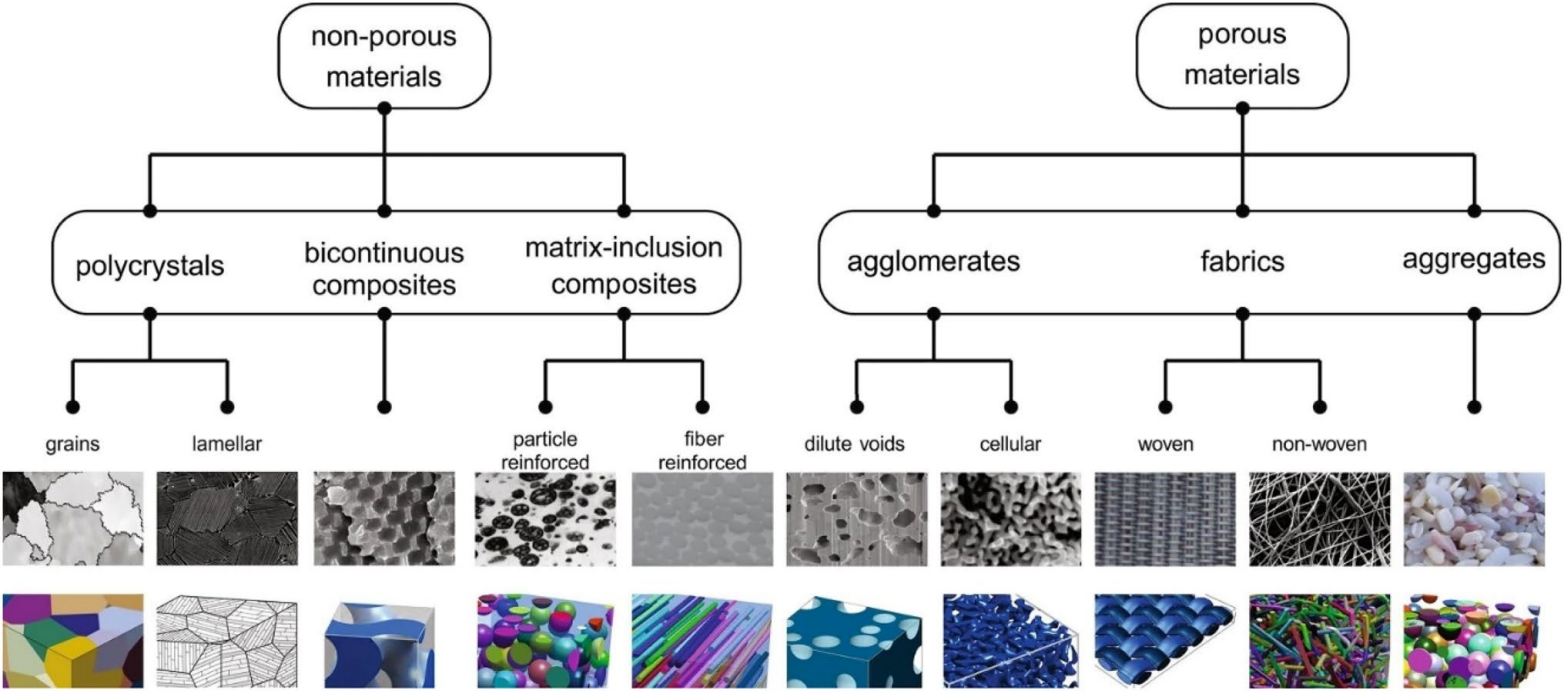
Reproduced from [174] where heterogeneous solids are grouped and further subdivided by microstructure classes

One conceptual approach is to use the materials classification shown in Fig. 21 (reproduced from [174]). This allows HVM to be viewed not only in terms of macroscopic material type: non-porous solid or porous solid, but also in terms of microscopic (microstructural) type. This simplification enables a unifying link between the macroscopic and the microscopic, making it easier to perceive common design elements across the various materials used within different HVM sectors. Since this is a geometry-based approach for the assessment of deviations from idealised design, it links well with IBSim.

Today, NDT of parts is widely used for evaluating porosity, deviations from design, cracks, or other flaw types. The pass/fail decision is made, however, based upon pre-determined design rules depending on the material type, the intended application, and the industry concerned. Although this approach can provide a qualitative ‘rule of thumb’, it does not take into consideration the full multi-physics combination of the detected features, e.g., the combination of a small pore that passes the design rule near an allowable deviation from tolerance, which may lead to a combined impact on performance that is greater than would otherwise be allowable. Furthermore, the use of pass/fail testing potentially leads to many parts being scrapped which could still be serviceable if used under slightly different loading conditions (e.g., in a different location within the assembly or in an assembly not expected to undergo the same extremes). The use of part-specific simulation to provide a more quantitative evaluation is the next generation for this type of testing. It is important to realise that despite the inclusion of macroscale deviations (porosity, etc.) into the simulation model, which is a significant improvement over the simulation of idealised design geometries, some flaw types may still go undetected. For instance, residual stress is not visible to XCT, and some microcracks may go unnoticed below the scan resolution. This section presents published applications of IBSim in HVM according to the industrial sector to which they’re most relevant.

#### Examples in the Energy Sector

Electrochemical energy devices are complex multi-phase, multiscale systems, consisting overall of solids (both porous and non-porous), liquids, and gases. Whilst the gases are not ‘designed’, the solid structures that contain them are. Liquid electrolytes are designed, but since IBSim is not used explicitly for this process, they will not be discussed further. Interested readers are directed to further reading [175, 176].

Electrodes found in lithium-ion batteries are porous solids, typically consisting of electrochemically active material and conductive additives, held together with a binder (often polymeric) [177, 178]. The pore network within these agglomerated structures contains mostly electrolyte, but depending upon the battery chemistry, gases evolve at the electrodes during operation that may also inhabit the pore space. Electrodes can also swell during operation as ions are intercalated. Thus, the battery is a pressure vessel that requires careful thermomechanical management. From a macroscopic perspective, then, deviations from idealised designs of certain safety features could be catastrophic, and IBSim has been performed alongside physical testing to validate safety models and to update standards [179]. From a microscopic perspective, no two electrodes are manufactured identical, but their microstructural metrics can be made similar with existing products conforming to a predefined range to guarantee performance. This range is the culmination of extensive previous research, and efforts continue to optimise existing solutions and search for alternatives. Thus, when we think of the deviations from the ideal, and how this impacts products and their performance, we need to investigate how IBSim is used for characterising microstructural metrics for electrochemical energy devices.

**Fig. 22:**
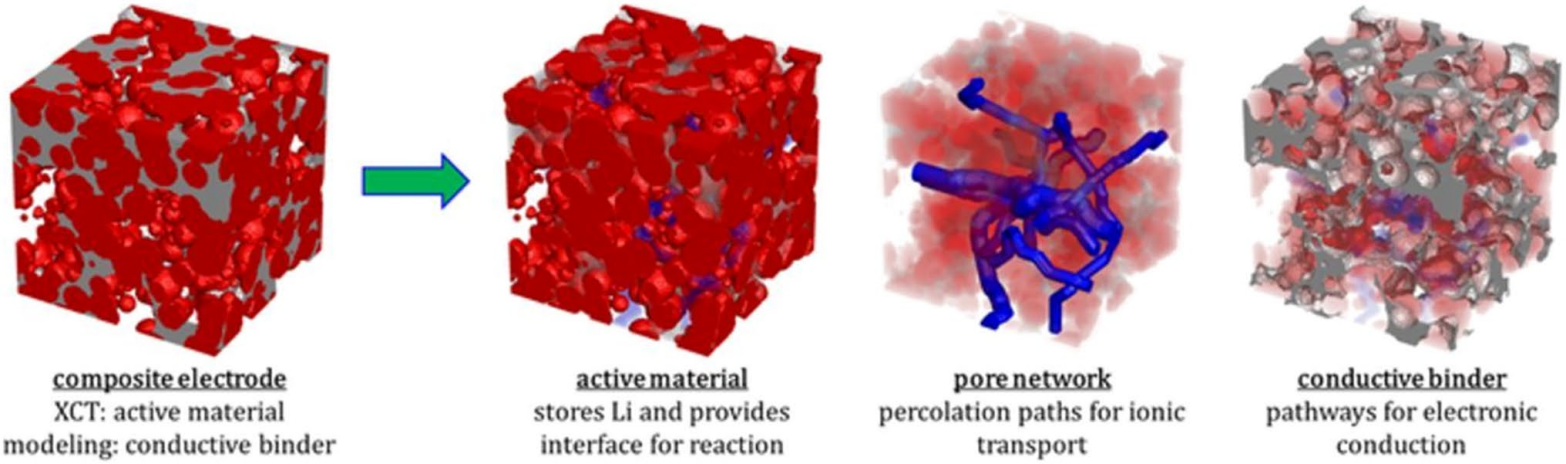
Showing the specific role of each microstructural phase (reproduced from [110])

A limiting factor of note within battery electrodes is mass transport restriction, where constrictions within the three-dimensional pore network can cause flow paths to be highly tortuous [86]. In one study the 3D microstructure of a single LiFePO_4_ electrode was acquired using XCT [110]. The effects of tortuosity within the pore network were examined using IBSim to assess the impact on ionic diffusion, a key performance parameter, showing the specific role of each microstructural phase (see Fig. 22, reproduced from [110]).

In work by Trembacki et al., an XCT dataset from [180] was used to simulate binder-phase morphology in nickel-manganese-cobalt cathodes [110]. The amount of binder can be varied within an electrode to alter electronic conduction. The calendering pressure can also affect porosity and contact between particles within the active material. These manufacturing parameters may be chosen to enhance energy or power density. Thus, deviations from these will impact intended performance. Interestingly, this study compares the finite volume method (FVM) and FEA on the same mesh, uncovering discrepancies when simulating electronic conductions at material interfaces where singularities can arise. This points to the possible use of adaptive meshes to improve simulation fidelity. Further examples of IBSim applied to research of batteries have also been published [181–185].

An area of interest within the energy sector, for macroscale simulation, is that of turbine blades. In a study of composite turbine blades, containing imperfections, wires and sensors, IBSim models from μCT data were performed with and without the inclusion of these features providing information on the effective properties and influence of these features on the performance [186]. At the microscale, IBSim has been used to assess cast materials, such as ductile cast iron (a non-porous crystalline solid), where it is normally assumed that the crack initiation stage has a negligible effect upon fatigue life, since early fatigue cracks are often observed in these materials. However, gas bubbles can be trapped inside casts, and shrinkage can lead to the formation of cavities, both of which serve to act as fatigue crack initiation sites. These defects are precursors to pre-existing cracks, acting like localised porous materials within the global non-porous solid. Simulating this pore space, its tortuosity and evolution, is therefore important for predicting fatigue life scatter. In one study a comparison between experimental fractography data and simulated fatigue life scatter was made [187]. X-ray μCT was used to obtain defect distributions within a range of specimens taken from rejected wind turbine hub castings, allowing a random defect analysis to be performed to predict fatigue life scatter.

The nuclear industry, another highly regulated sector, has stringent requirements on the quality and qualification of manufactured parts. In this context, IBSim models have been demonstrated to be useful to predict the performance of AM parts for nuclear applications [188]. IBSim was used to characterise a component manufactured with a bonding procedure for dissimilar materials used in water-cooled heat exchange components, identifying a defective joining process within ‘digital twins’ that would otherwise have comprised the component and surrounding substructure [189]. IBSim was used with a digital twin approach for detecting in-situ flaw formation in stainless steel (316L) impeller-shaped parts manufactured by L-PBF [190]. The digital twin approach was shown to be effective for detection of the three types of flaw formation causes studied in this research. Whilst in work by Evans et al. it was employed for high-heat flux components used within experimental nuclear fusion plants, where the debonding regions within carbon fibre composite-copper interfaces can be detected and quantified in silico from image data captured via high-resolution XCT [16]. At the microscale it has been used to assess the effect of microstructure and crystalline structure upon the thermal conductivity of graphite foams [94].

To round-off the breadth of applications of IBSim for the energy sector, we consider its application to semi-crystalline polymers. IBSim has been used to improve the performance of insulative porous polymeric coatings for offshore pipelines [191]. A common problem when simulating such multiscale systems is the computational complexity involved; molecular weight and distribution, size of crystallites, and microstructure all affect mechanical behaviour under various loading scenarios, and temporal effects such as work hardening can present themselves. The homogenisation of multiscale systems is an obvious approach to reducing simulation complexity, but continuum-level materials properties are not always representative at lower length scales. This is an outstanding challenge with the IBSim approach and common across application spaces.

#### Examples in the Aerospace Sector

A primary functional requirement for engineering products used in aerospace is for them to be lightweight. Thus, composite materials such as carbon fibre reinforced plastics (CFRP) have found widespread use. At the microscale, the fibre network of which they are composed is a key element in their performance, with factors such as directionality affecting damage evolution [192] and permeability [193]. X-ray μCT is invaluable for characterising these networks, but one particular challenge is to have a robust method to quantify microstructural features during postprocessing of images to obtain topologically accurate volumetric representations for IBSim models [194]. For example, loss of fibre edge definition can occur when imaging fibres made of low-atomic numbers, where X-ray phase contrast effects can cause them to appear thinner than their true diameter. Another area of significant industrial interest at this scale is the quality of welded regions within parts, with the potential for large pore spaces in these weld seams to create possible failure locations. An example of a welded light aircraft engine bracket shows the presence of a pore with diameter more than 1 mm in size [195]. Incorporating this into a simulation with ‘in-service’ loading conditions would identify the criticality of this pore for the intended application. In a study of welded seams using XCT and tensile tests, the authors compared simulations of the weld with tensile tests of the same samples, incorporating pores and surface irregularities into the simulation [14]. The stress–strain curves obtained from numerical simulations were in good agreement with experimental ones within the linear elastic regime; however, they deviated from each other when non-linear behaviour was observed experimentally since plastic behaviour was not implemented in the numerical models. The amount of pore content (high or low) and type of surface appearance (irregular or regular surface) was demonstrated to change the quantity of plastically-deformed areas and load-bearing capacity.

**Fig. 23:**
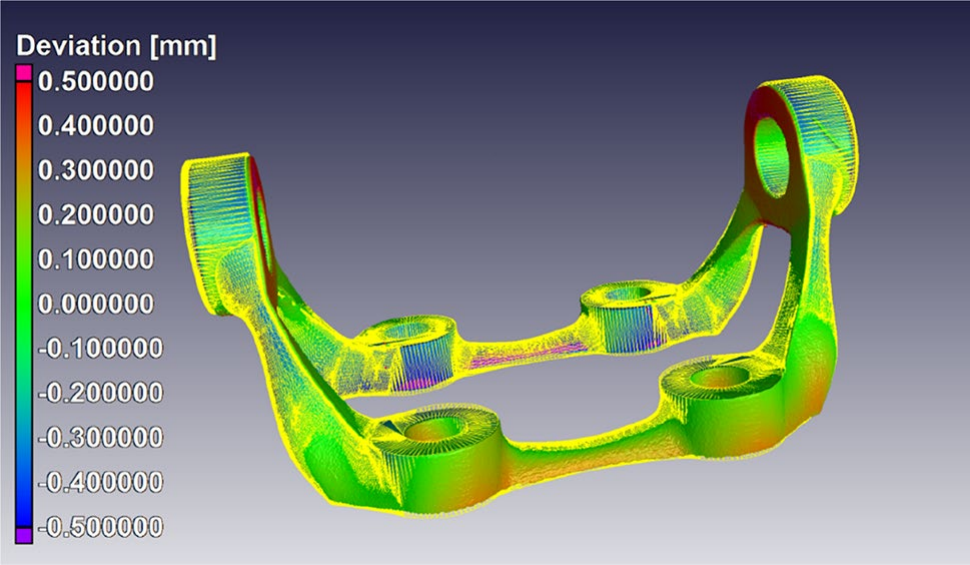
CAD variance analysis of an actual AM bracket (with colour coding showing deviation) compared to its CAD design (shown in yellow mesh). This example is from round robin tests [196] whereby AM parts were analysed by fixed XCT workflows, one of which is to evaluate differences between actual geometry and design geometry

An example of IBSim applied to the macroscale involved a study to evaluate the performance of parts which contained intentionally controlled defects [196]. The authors applied a simplified FEA method with a linear elastic assumption to μCT scans of AM aerospace brackets with varying sizes and locations of defects. The incorporation of these defects, in addition to the surface imperfections, into the simulations led to accurate predictions of failure locations using a stress-hotspot evaluation approach. The utility of XCT continues, as it also allows for the comparison of actual geometry to designed geometry, giving insight into deviations. The colormap, shown in Fig. 23, clearly identifies the largest deviations, which is especially important in critical locations of components, such as load-bearing sections with thin walls. The geometry shown is obtained by simulation-based design (also called topology optimisation or generative design) and the component is manufactured by metal AM. The arms are warped towards one another, affecting the alignment of the two holes, which is critical for its practical application. The decision, based on this analysis, was that this component required additional machining, and the ‘as manufactured’ bent arms might induce higher stresses when subjected to planned loads, which would not have been anticipated during design.

The ability to optimise the design of complex parts for specific loading regimes or functionality is particularly useful for targets such as reducing mass. This approach usually requires multiple rounds of simulation during the design process. Besides the use of simulation in the design process, the application of simulations to the final design for a quality control step is also important for highlighting possible limitations of the design and to check minimum safety factors [162, 197]. Given that the aerospace industry is yet another tightly regulated sector, it is key to the acceptance of novel techniques like AM that uncertainty in performance is limited. The advantages of AM for engineering structures with increasingly complex geometries are clear. For example, a range of designs for shape-changing thin-walled cylindrical composite structures were subjected to non-linear static FEA simulations using quadratic hexahedral elements [198]. FEA simulations were combined with experimental test results using digital image correlation (DIC), which allowed strain maps to be correlated with physical structures that had been fabricated by AM. Being able to predict failure in AM engineering products is also vital. Immersed-boundary finite elements can be used to predict the tensile strength of designs, simulating stress distributions from local stress concentrations and the location of crack initiation sites. This method does not require a conforming simulation mesh and is therefore suitable for complex porous solids where meshing may introduce singularities. This approach was taken by Fieres et al. using aeronautic parts consisting of AM AlSi10Mg aluminium alloy, with physical specimens destructively tested in tension [149]. They found there was good agreement between IBSim models and experiments for predicted and measured tensile strengths. Furthermore, they concluded that crack initiation location and onset could be forecast accurately.

Further examples of the application of IBSim within this sector include design improvements for composite Hart-II blades [186], biomimetic insect-sized micro wings [199], and nanoscale hexagonal plated wings for next-generation microflyers [200]. IBSim models of these structures require complex simulations coupling high-speed fluid flow, fluid–structure interaction, thermal flow, and mechanical vibration analysis. There exists a range of readily available software packages to explore different simulation options, but it will be important for anyone attempting to perform high-fidelity simulations on these IBSim models to balance meshing intricacy, component geometry complexity, and run time analysis.

#### Examples in the Medical Sector

Biomedical IBSim applications in general are based on patient medical imaging (e.g., CT/MRI) and are outside the scope of this review, and will, therefore, only be referred to for the purposes of context; the primary focus being manufactured parts used in the medical sector. IBSim has great potential for reducing material waste due to part rejection. A review was made of CT-based measurement techniques used for assessment of quality of bioengineering components [201]. Due to the critical nature of medical applications, FEA is often used [202, 203], especially in the design phase for new device development [204]. Originally FEA was widely used in orthopaedic studies for improved understanding of bone stress distributions, including bone-prosthesis structures and similar devices [205]. FEA has found particular use in dentistry and orthodontics [206–208] and spine research has also benefitted from FEA for improved understanding of the spine and spinal implants [209], whilst simulations involving the human skull have supported the understanding of head injuries even assisting forensic investigations [210, 211].

Due to the wide scope of applications of FEA in biomedical and biomechanical applications, some guidelines have been suggested almost a decade ago [212–217]. The medical sector is understandably highly regulated, and as such, use of novel methodology usually requires certification from a regulatory body (depending on the nation) before use is permitted with patients. Despite this, and due to the complexity in geometries and variability between cases in biomedical applications, IBSim has already found widespread use, including in the study of scaffolds for bone and tissue regeneration [218–222], implants [223–227], intracorporeal structures [228–230], biomechanics [231], and medical device design verification [232].

In a patient-based IBSim study of a medical implant, high stress locations were identified that led to eventual failure of the implant in the patient [12]. In this work, a medical CT scan of a patient was used to design a suitable mandibular implant geometry (patient-specific and porous) which unfortunately later fractured in the patient. An IBSim study was therefore conducted to correlate the actual failure location (from subsequent CT scans) with the location of high stresses by using IBSim models of the designed implant. This study highlights the potential of macroscale geometrical simulation of complex objects, informing the design, irrespective of manufacturing defects and imperfections. In this case, the manufacturing process was not checked, rather the design was flawed, though manufacturing flaws or imperfections can contribute to such failures. Similarly, fracture behaviour of human and sheep mandibular diastema fixated with titanium miniplates and screws under physiological muscular loads and variety of clenching modes (intercuspal, incisal, and unilateral) were simulated using IBSim models [233]. IBSim models of mandibular constructs made of Titanium scaffolds were simulated by using 3D FEA and multiscale modelling to pre-clinically determine the optimal mandibular geometry for a specific patient, where the influence of strut diameter and inter-strut distance in porous architecture on stress and strain distributions was quantified [234].

A particular challenge with IBSim, in comparison to conventional engineering simulations, is the computational expense involved. Reducing complexity of IBSim models is one solution and can be required if access to high-performance computing resources is a limitation. As with material characterisation, a popular method when considering microstructure in biomedical engineering, is to use RVEs with periodic boundary conditions to reduce computational expense. For example, one work used RVEs to homogenise the mechanical properties of Ti6Al4V structures for biomedical applications [223]. The IBSim models were subsequently solved using a four-node tetrahedral mesh via commercially available software. To validate their approach, models were manufactured by AM with electron beam melting (EBM), then subjected to mechanical tests. Whilst reported results appear limited, it does show that homogenisation of key design elements through RVEs can be a useful method for design improvements with reduced computational complexity. Another example of reducing computational complexity includes methods such as the extended finite element method (XFEM) [224], where the initiation and propagation of fracture paths in specimen-specific bone can be predicted. The benefit of XFEM is that it does not require a priori information about the crack path, and thus model re-meshing is not needed. For those interested, a review of FEA models and their validation for tibiofemoral joints is available [231].

### Customisation and Personalisation of Products

Nowadays, customers expect products tailored to their personal preferences, tastes, needs and lifestyle. The paradigm of mass production has therefore shifted in favour of customised production, which caters to the needs of individual clients or patients [235, 236]. The notion of personalisation is not unequivocally defined, although the common features of this approach indicated in the literature are customer preferences, customer participation in the product design process, customisation and information flow between customer and manufacturer [237, 238]. The level of customer involvement in the production cycle is thought to play a critical role in determining the degree of customisation, whereby the earlier the initial involvement of the client, the greater the customisation.

**Fig. 24:**
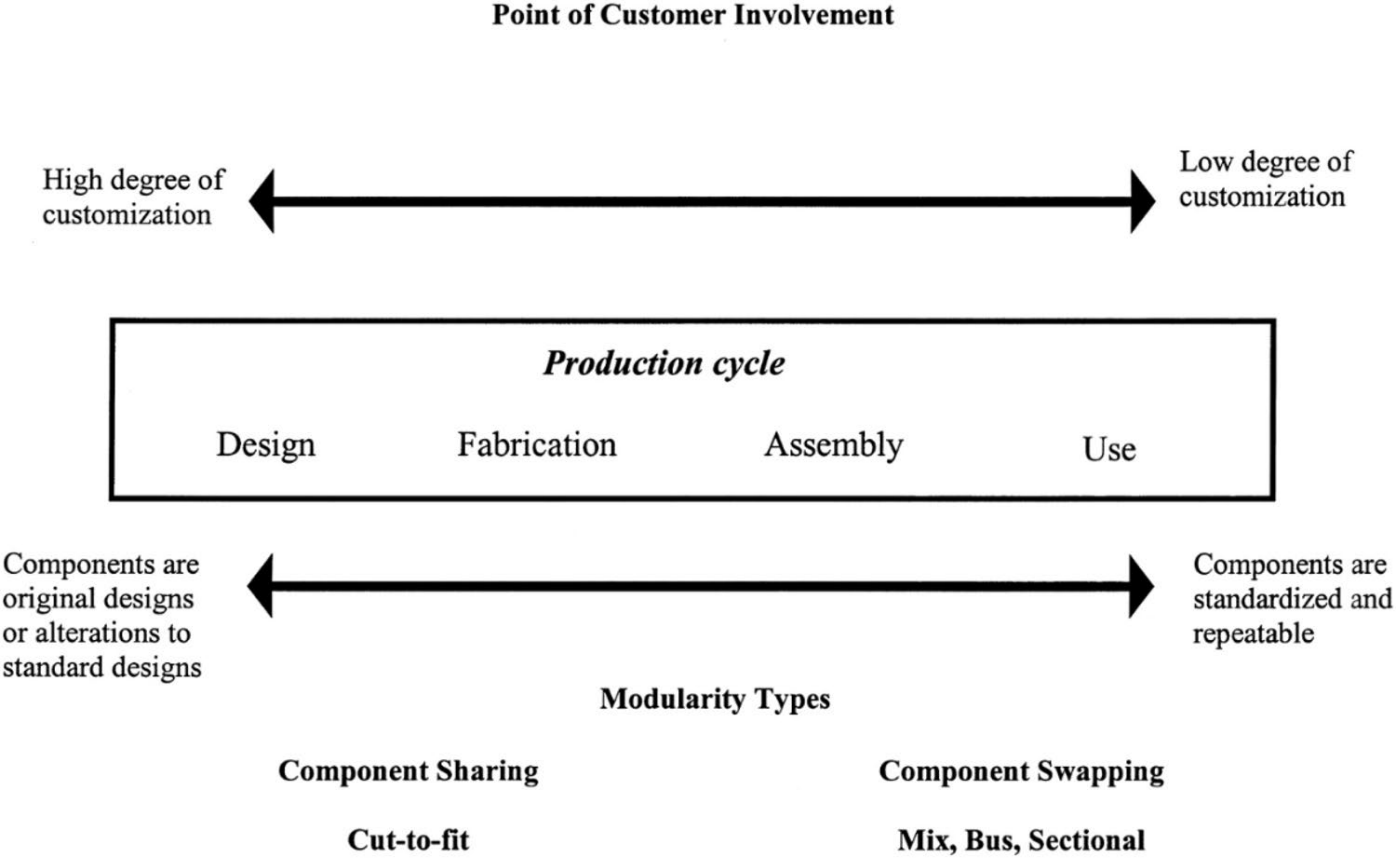
Customer involvement and modularity in the production cycle of mass customisation [239]

The term *‘mass customisation’* refers to products which are mass produced but where the consumer is offered options to customise the product. Mintzberg views customisation as taking one of three forms: pure, tailored, or standardised [236]. Pure customisation includes the consumer in the entire cycle, from design through fabrication, assembly and delivery and it provides a highly customised product (Fig. 24). Altering a basic design to meet the specific needs of a particular client is known as tailored customisation, and in standardised customisation a final product is assembled from a predetermined set of standard components [239].

To avoid confusion, and to ensure a clear distinction between customisation and personalisation, the term *‘pure customisation’* will no longer be used throughout this section. Instead, the term *‘personalisation’* will refer to bespoke/individualised products which are fulfilled at the personal level (i.e., a market of one). The automotive industry is a well-documented example of low and high degree of customisation [240]. Here, the customer can opt to customise the car from a plethora of options (e.g., colour, model, trim level etc.). The Porsche automotive company have recently ventured into custom car seating to allow the customer to choose between three firmness levels (hard, medium, soft). The 3D printed bucket seat is set to become a personalised product in the future, based on the customer’s specific body measurements.

To move from mass production to mass customisation, a company needs to invest in the right technological capabilities. Traditional manufacturing methods are restricted in their ability to create customised products as new moulds are typically required for each product. This subsequently leads to increased change-over costs associated with tooling and fixtures and extended timelines. AM has been widely acknowledged as the most appropriate manufacturing method of production of customised products, due to the lack of associated tooling required and the ability to produce highly complex geometries. AM covers a broad range of production technologies that fabricate products layer-by-layer, enabling 3D objects to be ‘printed’ on demand in a variety of materials. AM technologies such as stereolithography (SLA), 3D printing, L-PBF, selective laser sintering (SLS) and EBM lend themselves to manufacturing complex anatomic parts without any barriers of design constraints. Examples of customised and personalised products include implants [241], bone and tissue scaffolds [242] and prosthetics [243] within the medical sector, which appears to be the one currently most active in this field. Then closely related to the medical sector are customised personal protective equipment [244] and protective sportswear [245].

The most accurate analysis of processes and material behaviours comes from in-situ imaging and diffraction techniques. Combining imaging technologies such as XCT, FIB-SEM and MRI with the design freedom of AM has opened up new and exciting opportunities to customise and personalise products to many applications [246–250]. This has been particularly advantageous for industry such as healthcare to improve the effectiveness of diagnosis, planning, surgery, and clinical outcomes perfectly adapted to the patient’s specific anatomy or needs. However, a critical stage of this process is simulation modelling, e.g., using FEA. The ability to analyse and test how a product will react to certain environments (e.g., heat, force, microclimate etc.) and to predict structural strength and prevent failures is crucial where safety is paramount, e.g., both in patient-specific devices or custom wire baskets in aerospace.

Understanding the environment is important to all applications, but it is particularly complex in the case of medical applications where devices are intended to be used inside the human body. As organs and soft tissues already exist in the patient, it is imperative that the bone and tissue scaffolds integrate fully and do not cause infection or become ‘rejected’ by the body. Customised or patient-specific scaffold geometry can be gained by applying CAD software along with known individual patient anatomy parameters related to the defect site to create a 3D model. Computer modelling and FEA before 3D printing of a composite bone or tissue scaffold allows accurate identification of patient-specific anatomy and any variation in defect shape and size; subsequently ensuring the quality of the final medical model and product is not impaired [251].

The continuous demand for efficient and adaptive customised and personalised products relies heavily on IBSim techniques. In this sub-section we have therefore presented a variety of applications which have implemented IBSim in the development of customised and personalised products below. These include: Healthcare, Personalised Medicine, Prosthetics and Orthotics, Sport and Lifestyle, and Automotive sectors. Note, that this is not intended to be an exhaustive list, but instead an overview of the key applications observed as currently being of interest.

#### Healthcare

Arguably, healthcare is one of the most complex and challenging industries to produce customised and personalised products. Due to the differences that exist between humans, it is essential that all aspects of design and manufacturing are considered to ensure the device not only functions as required, but that it does not cause damage and/or harm to the body. The complex anatomy, sensitivity of the surrounding bones and soft tissues, and uniqueness of the defect or malfunction means IBSim can play a critical role.

Obtaining patient- or object- specific surface or volumetric geometry is important to assess the size, location, and overall fit of the device/product in relation to the individual. Static or dynamic simulations can then be run to investigate short- or long- term outcomes. The ability to run simulations that mimic the likely response of the device against patient-specific geometry is crucial for predicting the success of the proposed product and determining any potential flaws in the design which could be optimized. Simulations are also useful in understanding the behaviour of a material, particularly in time dependency models where wear and tear can occur over a given time period.

Applications of XCT with AM in a medical context are extensive, particularly in dentistry, where there is great demand for personalised products [207, 252–255]. Primary applications of XCT with AM in medicine include: the production of anatomical models, surgical guides, endoprosthetics and orthotics, stand-alone implants and scaffold implants [256]. These applications rely on the principles of reverse engineering, using patient XCT data to inform the design process. These advantages make AM invaluable in tissue engineering applications, where the production of micro-scale lattice structures is an intrinsic requirement.

It is also very common to see other imaging modalities such as MRI used in medicine as access to pre-operative data is readily available. In the same way as XCT, MRI data can be used to reconstruct patient-specific 3D geometry. Whilst the main focus of this review is XCT due to HVM applications, it is worth acknowledging that MRI has several benefits over XCT whereby it does not exposure the patient to ionizing radiation. It is also extremely useful in examining soft biological tissues, whereas XCT is particularly useful for examining materials with a high atomic number [27]. Acquisition methods such as cone beam computed tomography (CBCT) have considerably reduced the radiation dosage to the patient, but there are increased concerns regarding the collective radiation dose given for medical purposes [257]. XCT and CBCT have proven particularly useful for complex dental cases involving surgical planning, detection and treatment of tumours and reconstructive surgery of the mandible; where personalised geometry is acquired for point cloud data processing and analysis using FEA [247, 258]. Publicly available tools such as the MATLAB ‘Torsion Tool’ and the ‘Bone deformation Tool’ allow personalised geometry to be generated quickly in an OpenSim musculoskeletal model [259]. Such tools can estimate personalised measurements within seconds but are often limited to a single model.

Yu et al., integrated CBCT, reverse engineering, CAD, FEA and rapid prototyping to fabricate an accurate customised surgical template for orthodontic mini screws [260]. IBSim was of particular importance to this study as the use of CBCT was able to measure the interradicular spaces with greater accuracy and reproducibility than other imaging modalities (such as multidetector CT), whilst the FE models allowed biomechanical evaluation of the customised surgical template with increased clinical stability. Whilst full IBSim was utilised in this study, further simulations could have been implemented to assess the biomechanics of the surgical template post-surgery in order to reflect the effect of soft tissue inflammation and screw loosening. The authors did acknowledge, however, that there were large gap sizes between the surgical template and teeth/mucosa due to fitting errors with the soft tissue image reconstruction [260]. This could also potentially be rectified by further FE analysis.

Another example is presented in a recent paper by Dot et al., whereby CBCT, intra-oral scans and subject-specific FEA were used to track the 3D orthodontic tooth movement in a patient undergoing canine retraction over a seven-month period [261]. An iterative closest point (ICP) algorithm was used in Geomagic Studio software to align and register the scans and segmented canines at different stages (i.e., initial, and intermediate). Open-source software ITK-SNAP, 3D Slicer and Mimics were then used to create 3D models and calculate rigid body displacements of the canines. The 3D models enabled preliminary FE models to be developed and validated [261].

There is a breadth of literature that focuses on the design of implants, particularly for craniomaxillofacial surgery where gunshot wounds or tumours have required mandibular or cranial reconstruction. Parthasarathy reviews a number of articles related to 3D modelling and custom/personalised implants in craniofacial surgery, specifically in relation to application of CAD and computer-aided manufacturing (CAM) technologies with different materials [241]. The conversion of CAD models to STL format for manufacturing is common. The use of CAD/CAM in dentistry has allowed various morphologies from different devices with high accuracy, thereby increasing treatment opportunities in some clinical situations. This technology combined with a L-PBF machine can provide porous titanium structures with complex geometries that control the internal architecture [262]. In addition to L-PBF, SLS and EBM have also been used to facilitate the direct production of titanium, chrome cobalt and polyetheretherketone (PEEK) implants with engineered properties that match properties of the tissues at the region of implantation [250, 263, 264].

**Fig. 25:**
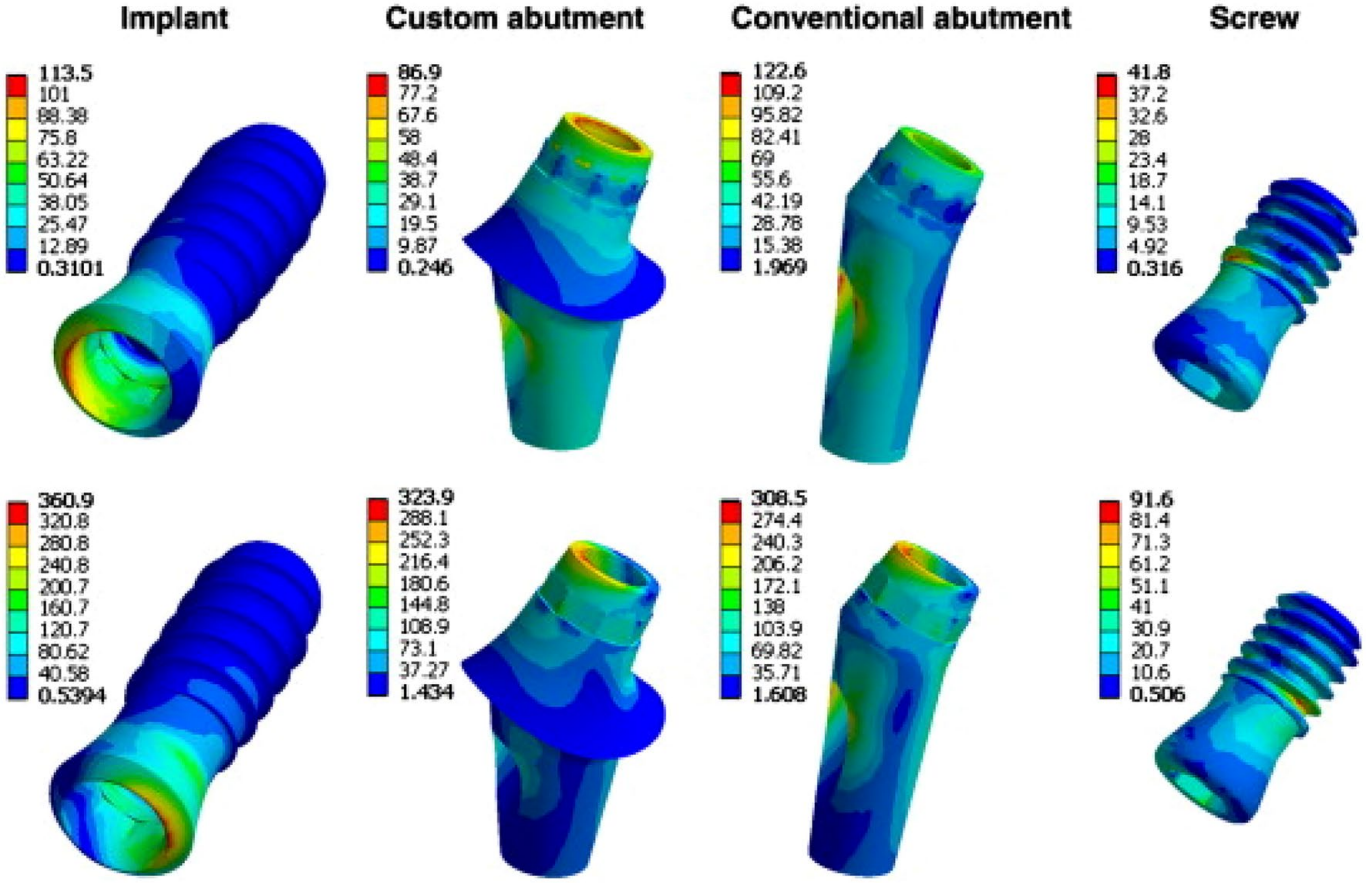
Von-Mises stress distribution of implant, custom abutment, conventional abutment and screw under loading along the abutment long axis (row 1) and along the implant long axis (row 2) [265]

Wu et al., used a combined methodologic approach to assess the biomechanical performance of a conventional and custom angled dental abutment without the need for wax and cast [265]. Numerical models were acquired through optical scanning of the dental cast, with the optical gaging products video measuring system used to obtain detailed shape parameters. A virtual prosthesis was then preliminarily positioned and probed for interference using the “Collision Detection” function based on the “Least Square method” to fix the prosthesis in the required position. Geometry of the patient’s bone was taken via medical CT images and an FE model generated in ANSYS Workbench 11.0. The results of the von-mises stress distribution simulation can be seen in Fig. 25 where no distinct difference in the stress distribution was found using the custom or the conventional angled abutment [265].

XCT-based FEA has evolved into a standard tool for the biomechanical evaluation and optimisation of porous bone tissue scaffolds. Systems such as the Skyscan 1272, Bruker-MicroCT are commonly used to obtain high resolution 3D scan data of the scaffold. The reconstructed images and morphometric and structural analysis can then be performed using Bruker proprietary software NRecon® and CT-Analyser CTAn® [266]. The primary advantage of XCT for bone tissue scaffolds is that it is a non-destructive imaging technique which is capable of providing a comprehensive set of data. However, the accuracy of the analysis, is highly dependent upon several parameters such as specimen preparation, parameter settings during the acquisition, and reconstruction of the images [267].

**Fig. 26:**
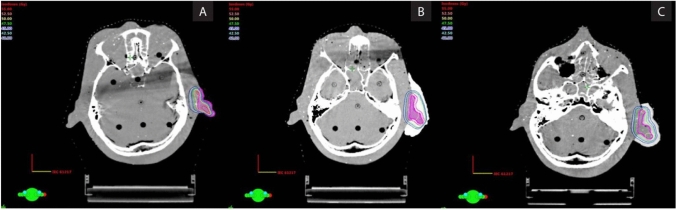
Isodose lines calculated by means of IBSim CT simulations of a phantom head **a** with no bolus, **b** with a commercial bolus and **c** a bespoke AM bolus [285]

The majority of literature surveyed in this paper utilised a combination of imaging techniques e.g., XCT and scanning electron microscopy (SEM) in order to examine the characteristics of the scaffold. 3D CAD designs and theoretical equations/simulations were also common for designing the porosity of the scaffold but there have been reports that certain CAD simulations over-predict scaffold performance due to limitations in simulating micro-topologies [268]. FEM proved useful in investigating and optimising the mechanical behaviour of the scaffolds [269, 270]. The use of FEM was also able to measure the sensitivity of scaffold properties (e.g., to the filament diameter, the variations of porosity and surface area). The review paper by Podshivalov et al. describes the state of the art in multiscale computational methods used in analysing bone tissue for personalised medicine is summarised by [271]. Challenges on optimization of 3D-printed customised bone scaffolds is presented in a recent review paper by Bahraminasab [251].

#### Personalised Medicine

Personalised medicine (often referred to as “precision medicine”) is an emerging field which will significantly benefit from the implementation of IBSim. In the US, the Food and Drug Administration reported 38% to 75% of patients for whom medication was ineffective for a number of conditions from depression to cancer [272]. Therefore, the ability to tailor a drug dosage specific to an individual where ingredients can be adjusted based on the patient’s age, gender, weight, genetic factors and previous responses to different dosage levels, rather than using the conventional dosage forms may diminish all potential adverse effects [273–275].

For example, IBSim techniques are invaluable for radiotherapy treatment planning as they can be used to; predict tumour response to radiation, reduce uncertainty in the prescribed dose distribution and spare organs at risk [276–280]. However, accurately calculating the perturbation effects of the interfaces between materials of vastly differing anatomic number (e.g., lung, bone and/or air) is complex, and as such, has often been solved using the Monte Carlo method [281]. A recent study by Roncali et al. looked at personalised dosimetry for liver cancer Y-90 radioembolization for a single patient [282]. CBCT was used to segment the hepatic arterial tree to predict microsphere transport using multiscale CFD modelling and Monte Carlo simulation. Bespoke manufacturing is also used within personalised radiotherapy treatment, mainly shielding blocks to protect regions of the body not intended to receive a dose [283]; and boluses to alter dosing received from the beam [284]. There are examples where the performance of custom boluses have been modelled with IBSim [285] (Fig. 26). Firstly, the head geometry of a phantom was captured via CT scanning, the bolus was designed to fit the topography of the ear and manufactured with AM. Once placed on the phantom, the head was re-scanned, and the data used in a simulation of a CT scan to evaluate the dosimetric properties of the custom bolus. It was found that the custom boluses better fitted the irregular surfaces, and this enhanced the dose that would have been received by the patient.

IBSim has not only played a pivotal role in the development of personalised medicine but also in the storage stability of pharmaceutical products. One example of this is a study by Zhang et al., which characterized a lyophilized drug product (a freeze-drying process that removes water from a drug product via sublimation) [220]. The study involved the use of high-resolution X-Ray microscopy to collect 3D volume data from lyophilized drug samples and quantitatively characterise the microstructures of the lyophilized drug [286].

**Fig. 27:**
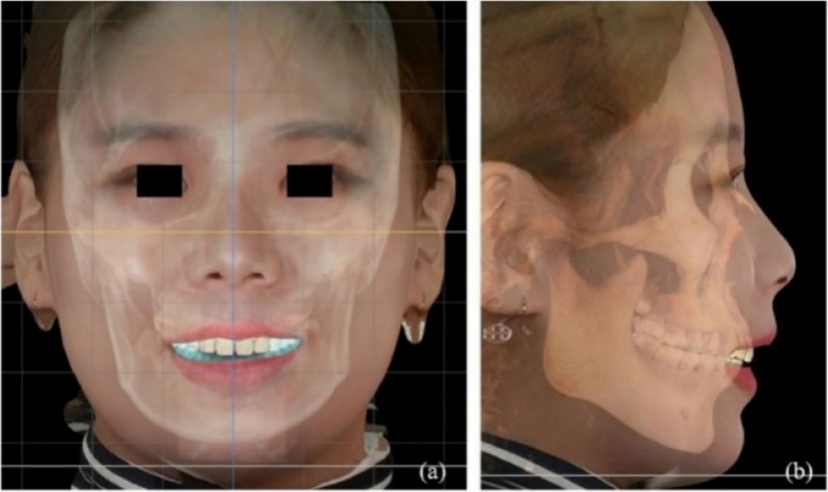
A digital twin reconstructed by the fusion of facial scan and CBCT images. **a** Coronal view of the face. **b** Sagittal view of the face [288]

Examples of digital twin applications and the ethical issues that arise when digital twins are applied to model humans for personalised medicine are presented in a recent review paper by Kamel Boulos and Zhang [287]. Of note, is the study by Cho et al., who assessed the facial profiles of Korean adult females using facial scans and CBCT imaging (Fig. 27). Digital twins were reconstructed to evaluate and compare the sagittal relationship between the maxillary central incisors and the forehead before and after orthodontic treatment [288]. This technology is somewhat in its infancy with regard to personalised medicine, but it is interesting to see this implemented with full IBSim. The concept of the digital twin for mass customisation enables new options for product manufacturers as seen in the aerospace industry [289]. Over the next decade, we are likely to see more research involving digital twins to personalise medicine, but this will require solving a wide range of technical, medical, ethical, and theoretical challenges.

#### Prosthetics and Orthotics

Researchers have been interested in computer-aided prosthetic socket design since the early 1960s, however, FEA was not introduced to prosthetic socket and orthosis design until the late 1980s [290]. Prefabricated prosthetic and orthotic products are readily available and less expensive than custom products; however, customised, or personalised products that take individual characteristics into consideration are considerably more comfortable and functional for the wearer.

Examples of mass customisation of orthoses to date have largely consisted of foot and/or ankle–foot orthoses (AFOs) [291]. All studies from 1990 to 2015/16 have been summarised in the review papers by Jin et al. [292] and Chen et al. [293]. Each of which have discussed the progress of AM of custom prosthetics and orthotics and the benefits over traditional plaster moulding techniques. More recently, Mali and Vasistha presented an efficient solution for the manufacture of an AFO using reverse engineering software to obtain a refined model via data repair [294]. A Steinbichler Comet3D™ structured blue light scanner was used to obtain the geometry of the diseased foot and the generation and post processing of the cloud data points were conducted using the proprietary software Cometplus™ and Autodesk® Meshmixer. FEA of the AFO was performed in Autodesk® Fusion360™ for three different materials. Optimization of the orthosis resulted in increase in safety factor, higher strength and lesser displacement when compared to a non-optimized AFO [294]. Agudelo-Ardila et al., proposed a similar solution for an upper limb orthosis whereby a subject’s hand and forearm were scanned using a structured light 3D scanning system [295].

**Fig. 28:**
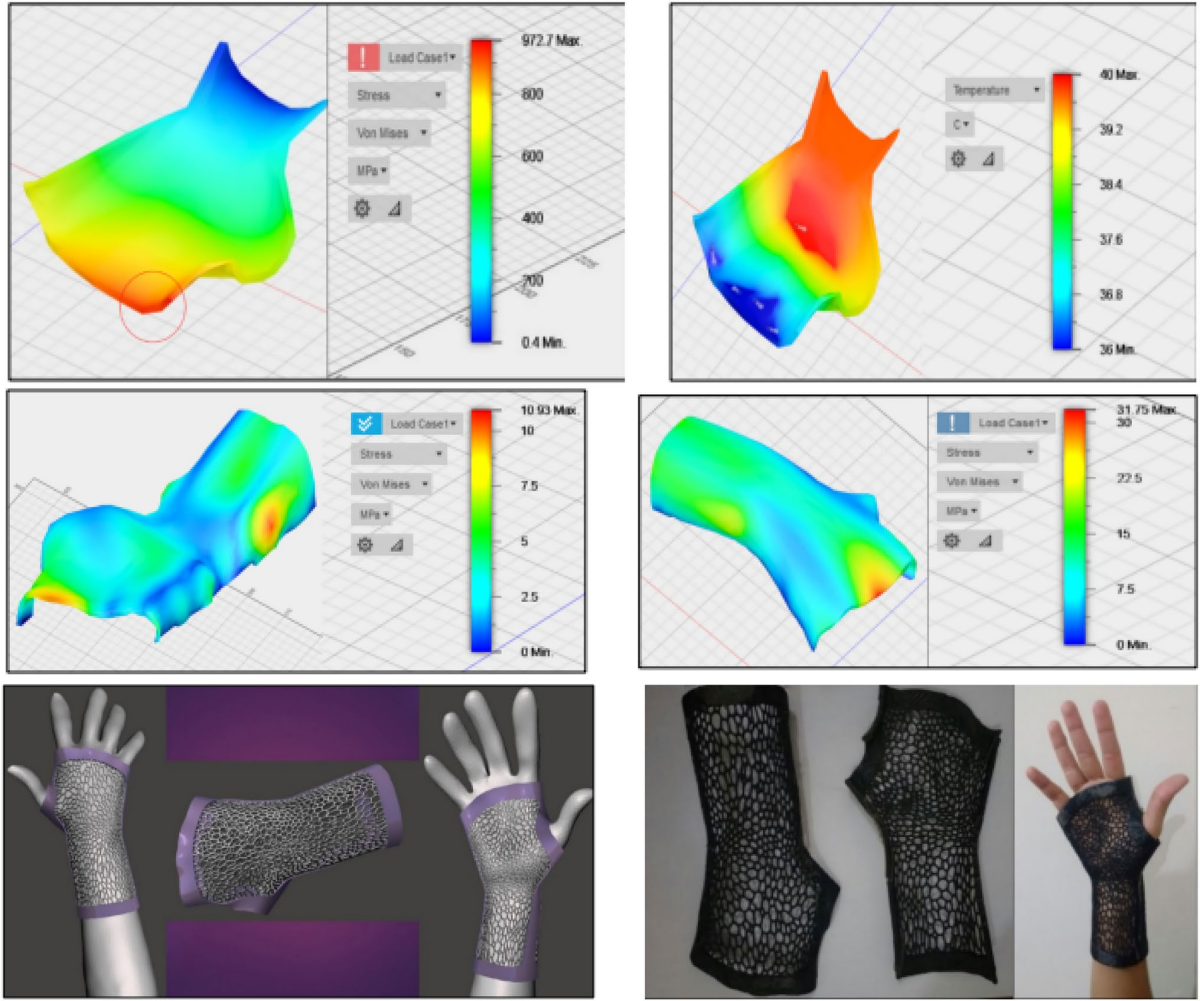
FEA analysis showing the stress and temperature analysis of the lower and upper part of the orthosis (top and middle row respectively), and the application of Voronoi patterns to reduce the material in the resulting 3D printed custom orthosis (bottom row) [295]

The STL model was processed in Canfit and Meshmixer software. FEA was utilised to perform structural simulations to determine when the material will deform or collapse (Fig. 28). Biomimetics were implemented from Voronoi structure (as an alternative for modelling cellular structures) and met the objective of material reduction, consequently leading to a lighter orthosis (Fig. 28). Both these studies demonstrated the effectiveness of IBSim not only as a useful tool for evaluating structural feasibility but also in making design decisions, reducing problems associated with plaster moulds and thus achieving a custom-made orthotic that is optimised for the patient [294, 295].

**Fig. 29:**
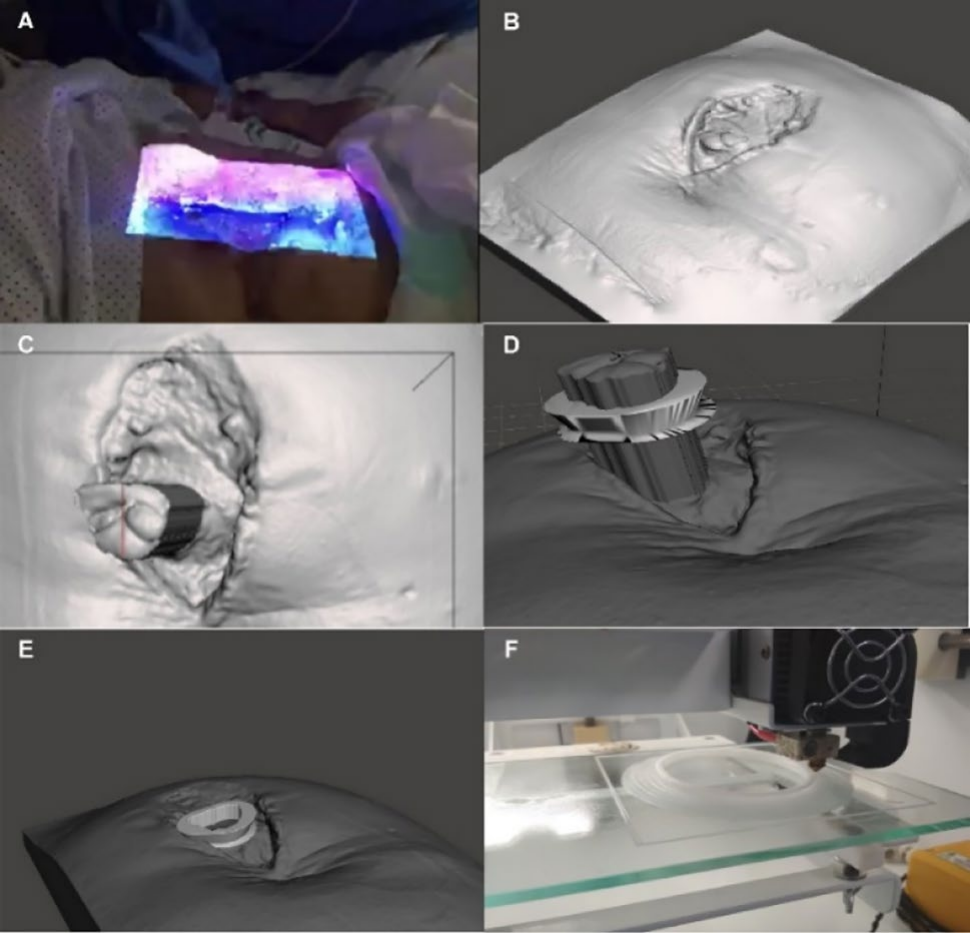
**A** Process of taking pictures with the bioscanner. **B** Images obtained with the bioscanner. **C** Measurement of the exposed intestinal surface dimensions for device design. **D** Verification of the suitability of the prosthesis by extrusion of the fistulous surface. **E** Placement of the device on the image of the bioscanned wound to determine the correct adaptation to the patient. **F** 3D printing of the bioprosthesis [244]

In addition to AFOs, there has been the development of custom prosthesis for the management of entero-atmospheric fistulas whereby an Einscan pro + , 3D shining scanner coupled with CAD software was used to capture the geometry of the fistula and create a polycaprolactone personalised ring-shaped device [243]. The device was then placed on the image of the wound to verify the customisation and placement (Fig. 29C–E). Virtual simulations allowed the tolerance margin to be calculated which was important in ensuring the prosthesis did not press on the fistulous tissue.

**Fig. 30:**
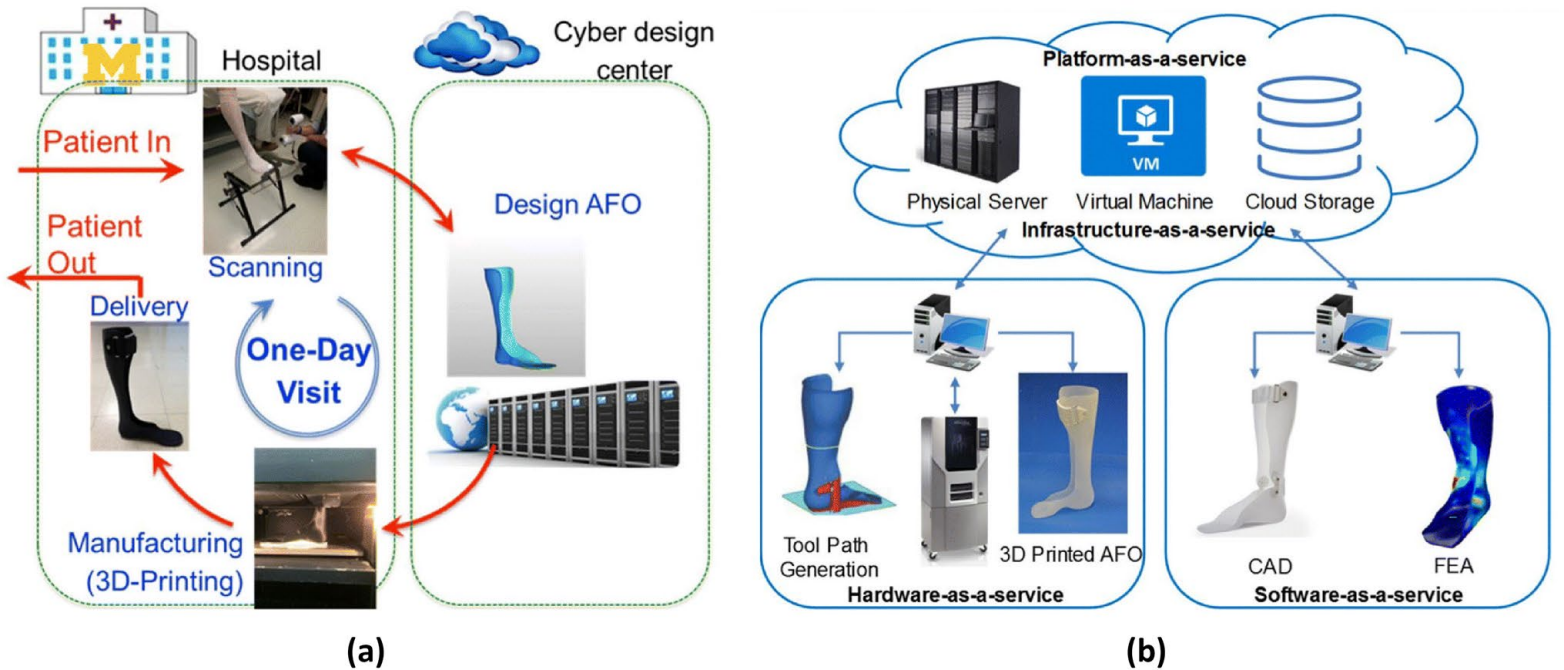
** a** Overview of the Cyber Design and Additive Manufacturing (CDAM) system for custom Ankle Foot Orthoses (AFO). **b** Illustration of the interaction between the hardware and software systems with the cloud storage system [297]

Ideally, any product that is designed for the treatment of an individual patient with specific illness, disease or injury needs to be fully personalised. The emergence of AM technologies allows the fabrication of custom-made orthoses in a cost-effective way [296]. Interestingly, in a 2020 review on the use of AM to produce lower limb orthoses, only three of the seven AM technologies available have been explored (vat photo-polymerisation, material extrusion and powder bed fusion). Material extrusion was found to be the most affordable AM technology but limited to the use of polymers [296]. SLA was considered an unsuitable method for manufacturing AFOs due to limited bending and fatigue strength. Instead, FDM was selected for customising AFOs. One of the interesting points mentioned in the review paper was the ability of Cyber Design and AM (CDAM) to assist the design phase of orthoses [296]. Fig. 30 shows an overview of the CDAM system developed by Shih et al., which aims to improve the fit and comfort of custom orthoses and prostheses, and enable users to solve complex design and analysis problems (e.g., FEA, optimization, visualisation) [297].

**Fig. 31:**
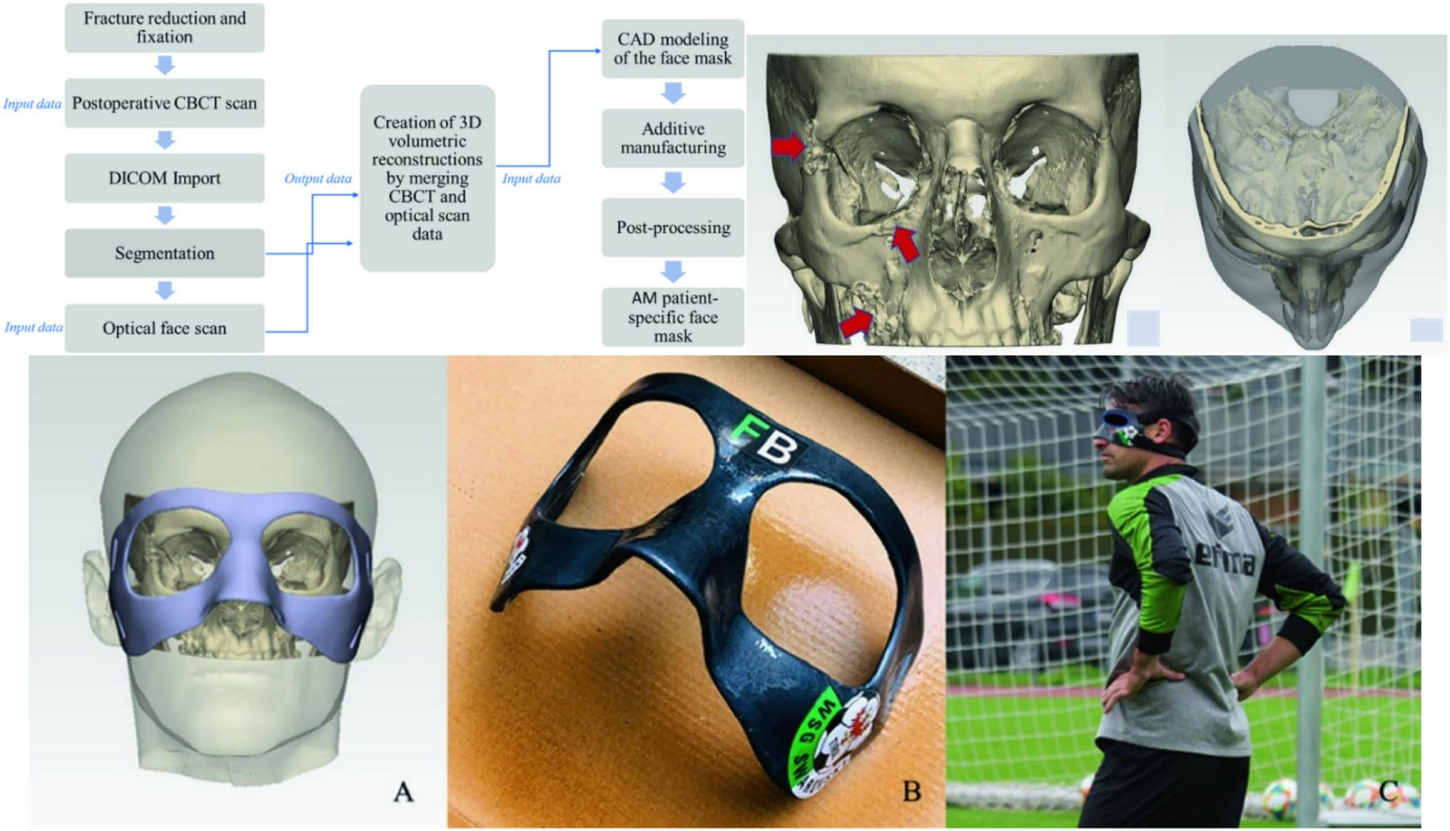
Top: An overview of the schematic representation of the digital workflow with Postoperative CBCT 3D volumetric reconstructions. Bottom: **A** 3D computer-aided design and planning **B** FDM printed carbon-reinforced PLA face mask **C** A professional soccer player with a customized face mask during his sport’s practice session [298]

Sharma et al., recently proposed a methodology for designing highly customised 3D printed facial protection orthosis for rehabilitative management in patients with sports-related maxillofacial injuries [298]. A postoperative CBCT scan was imported into Materialise Interactive Medical Image Control System (MIMICS) medical software in order to segment the region of interest and generate a 3D volumetric reconstruction (Fig. 31). A 3D optical scan was also taken to corroborate the soft tissue components and create digitised surface geometry of the face with a triangular mesh. An ICP algorithm, surface registration protocol (n-point and global registration) was accomplished between the CBCT scan and optical face model [298]. Conventional methods for fabricating a mask were eliminated using IBSim, thus eliminating a very time-consuming process. The imaging and AM workflow demonstrated in this study could be applied to numerous applications, but computational simulations are not presented. The maxillofacial orthotic was based on an adult male whose face shape is unlikely to change (as the bones are fully fused). For a child or adolescent who has not reached skeletal maturity, simulations could help predict the optimum design of the orthotic and also identify when the orthotic is no longer effective. Digital workflows similar to the abovementioned study have been found for cervical collars [299].

#### Sport and Lifestyle

With the growing concern surrounding head injuries in sport, there has been increased research into personal protective equipment [245, 300]. 3D surface scans, medical imaging and/or 3D anthropometric data, can be used to acquire the head geometry and proposed protective headwear. The detail of this geometry depends on the type of methodology implored and the material properties assigned to the head and proposed helmet design. Corrales et al., addressed these limitations by developing a numerical model of a modern football helmet by integrating two headforms and assessing a range of impact conditions [301].

**Fig. 32:**
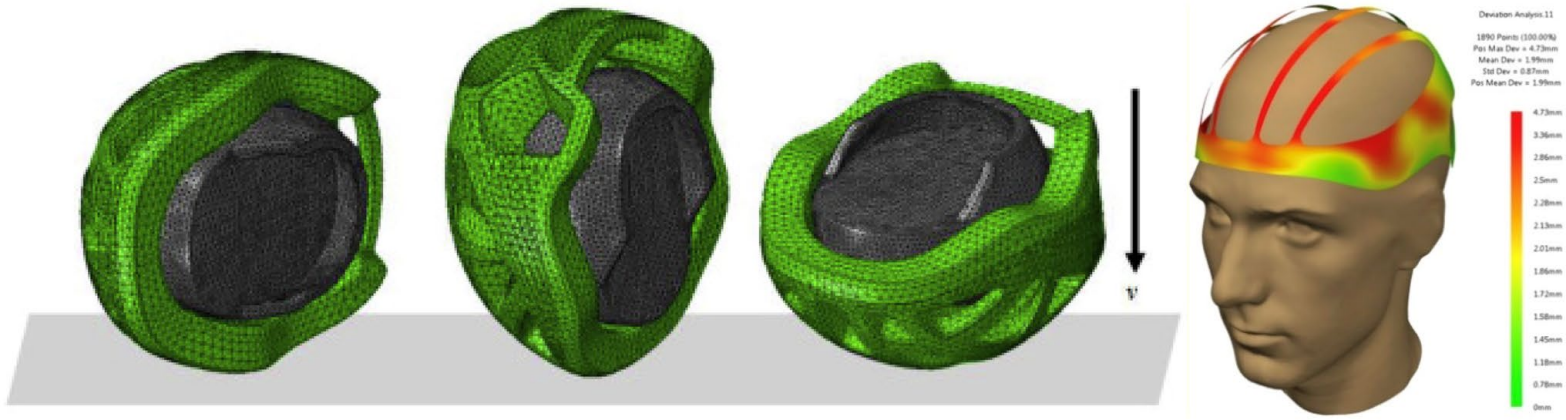
Impact locations of the customized helmet: side, front and top (left) and deviation analysis of a participant’s customised helmet (right) [302]

Virtual impact test simulations can assess which design is most effective at protecting the head (in terms of structural and kinematic response to impact) and can be evaluated by experimental impact testing. Fig. 32 shows an example of a custom-fit bicycle helmet model proposed by Ellena et al. whereby 3D anthropometry, reverse engineering techniques and computational analysis methods were used to assess accuracy of fit [302]. This study demonstrated that the fit accuracy of the custom-helmet models was significantly increased compared to three commercially available helmets, and their method complied with the relevant drop impact test standards. However, the authors acknowledge that the mechanical properties of the available materials used in their custom design differ significantly from well-known foam material, and that a combination of AM with moulding techniques would be a likely outcome in future studies.

Safety standards and certification have most likely contributed to the lack of mass customisation systems of helmets to date. Industry customised helmets, by brands such as Bell Sports® (Rantoul, Illinois, USA) currently meet the US Standard, but the information in how the Standard was achieved is not disclosed. In addition to helmet designs, there has been a growing trend among shoe manufacturers (e.g., Nike, Adidas) to introduce customised shoes to improve fit, comfort and performance [303]. Custom-fit mouthguard designs, have also been studied extensively to evaluate the tooth stresses and strains, shock absorption, and displacement during impact [304, 305]

#### Automotive

The automotive industry is a highly competitive market where there is a constant and ever-increasing demand for personalised products. The CES 2020 Survey by CITE Research Dassault Systèmes found that 83% of 3000 consumers in the US, China and France expect products or services to adapt in a matter of moments or hours [306]. Only 21% will wait four or more days for a personalised product or service to be delivered, but they are willing to pay more (an average 25.3%) for personalisation [306].

Consumers’ increasing demand for personalisation capabilities, coupled with their refusal to incur any extra wait time for delivery, sets up a major design challenge for engineers. Some luxury carmakers have embraced this challenge and launched customisation schemes to deliver tailored designs to meet customer requirements. MINI Yours in 2018 enabled customisation of small parts such as steering wheels, decals, and colour combinations [307].

**Fig. 33:**
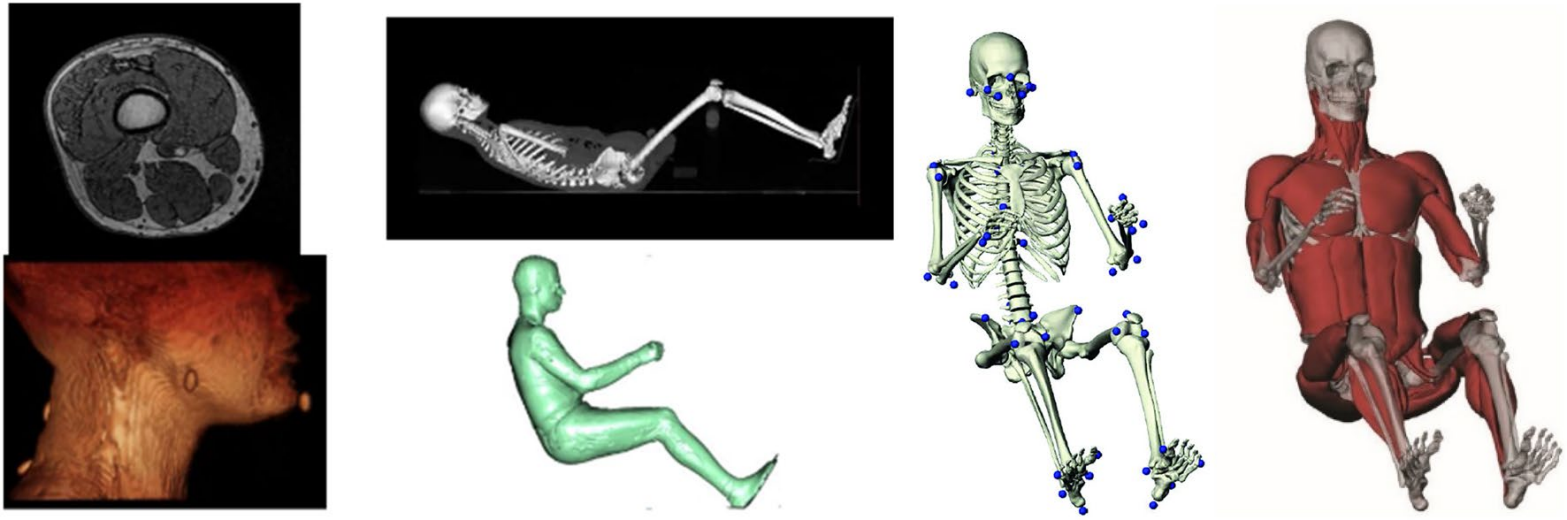
Overview of data collected for the M50 seated FEA male. Left—conventional MRI thigh cross section and lateral view of neck; middle—quasi-seated CT scan and external body laser scan in seated posture; right—full human body model (bone and muscle) [308]

FEA of vehicle crash analysis and crash test dummy simulation uses software such as LS Dyna to run explicit analysis. To predict and assess the response of occupants in a vehicle crash, specific models can be generated to include a number of individual characteristics (e.g., gender, age, height, weight, etc.). Automatic seat belt draping, pre-tensioning and body interaction with the seat will influence the sophistication of these simulations. The M50 seated finite element male is intended for use in simulations of vehicle crash and was developed in LS-DYNA Rev. 4.2.1 [308]. Multi-modality imaging comprising MRI, CT, and a 3D digitiser (FARO Technologies Inc., Lake Mary, FL) was used to capture subject-specific anatomy (Fig. 33). Non-linear dynamic FEA simulations have also been used to predict the magnitude of impact forces, G loading, deformation, stresses as a function of race car velocity and the angle of impact of a novel airbag technology [309]. Custom clutch designs to determine the suitability of a specific material to be used in real production have also been analysed [310].

### Image-Based Simulations in Biomimicry

When the imaging techniques already discussed are used to image biomaterials, the detailed multiscale images produced provide invaluable information, which may be used to better understand nature and consequently used to solve engineering problems. Biomaterials are the result of hundreds of millions of years of evolution in nature, a natural iterative optimisation method. They are known to be multifunctional, for example, having the functions of impact or fracture resistance [311, 312], armour and protection [313, 314], strength and durability [315], light weight for flight [316]. Meyers et al. [317] stated that there are two levels for the development and implementation of concepts extracted from nature (i) design and concepts which are inspired by nature but applied with different materials and conventional processing techniques such as self-cleaning surfaces inspired from lotus leaf for hydrophobicity [318], (ii) bioinspired structures which are mimicked in molecular level by means of self-assembly and molecular engineering such as high-performance ceramic–metal composites designed from ice-templating process [319]. Plessis and Broeckhoven reviewed the ability of AM in biomimicry and the contribution of biomimetic structures to new engineered products and applications [320]. In this section we present papers which have used IBSim as part of their biomimicry research collated into three main approaches.

Previous to this review, a few other invaluable works have concentrated mainly on a specific part of the IBSim technique. For example, Cooper et al. reviewed IBSim FEA models of tibiofemoral joint with respect to specific titles such as generic knee models, mechanical models [331]. Ha and Lu looked at recent research on bioinspired structures and materials in terms of their energy absorption capabilities, this collated examples from bamboo inspired structures to a bone-mimetic crash box [321]. Vasarhelyi et al. investigated the use of μCT and 3D analysis in characterisation of advanced materials in application-wise (bioinspired materials, structural materials, energy and environment) with a relatively sparse amount of literature [322]. This section, on the other hand, attempts to collate the broader literature of biomimicry applications of IBSim under the titles of three main IBSim approaches used in biomimicry of materials in order to design new materials or upgrade and/or repair existing damaged materials.

#### Imitation of Biomaterial Architectures in CAD-Based Product Design Using 3D Image Data

**Fig. 34:**
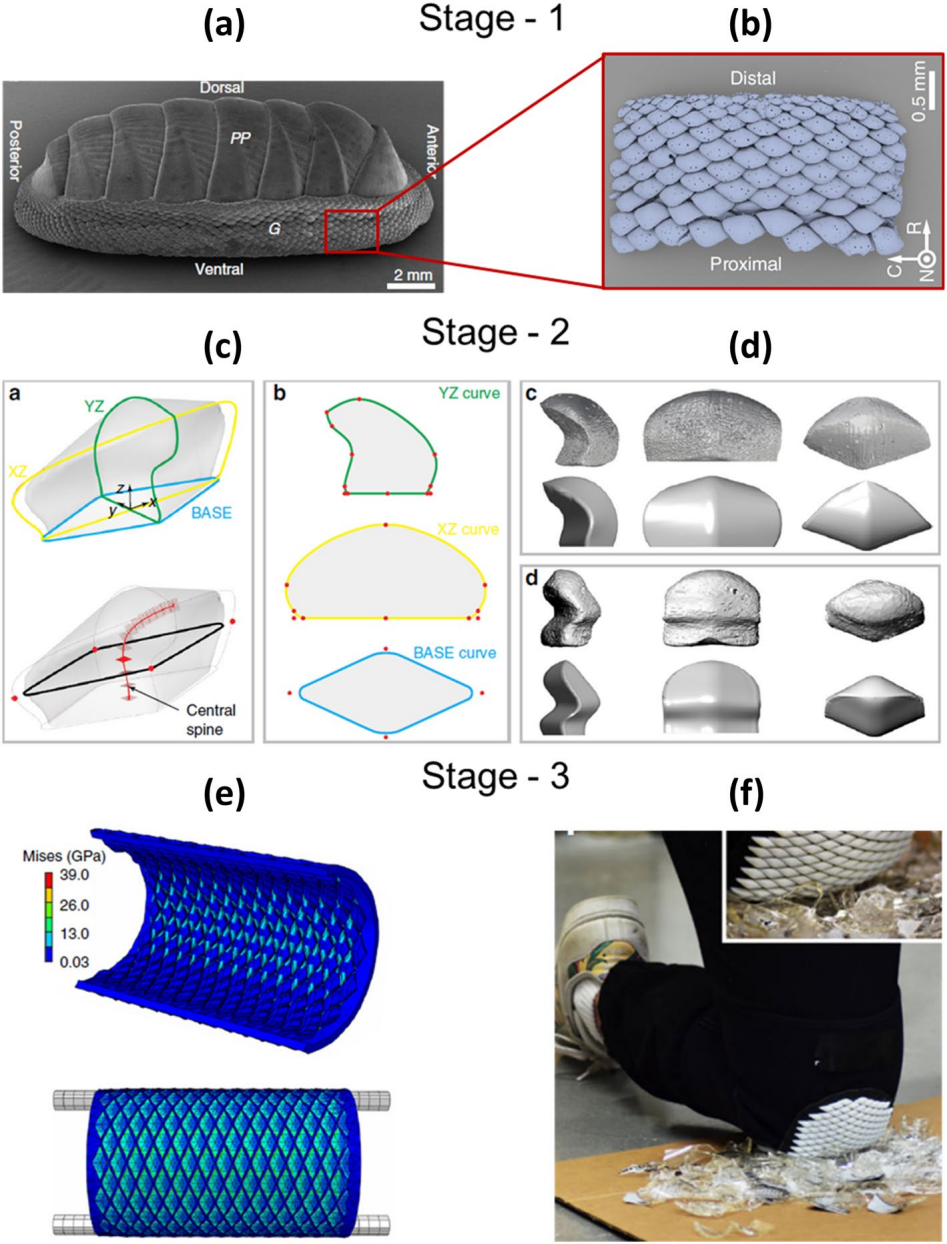
** a** SEM image of chiton’s armour and **b** segmented X-ray volume; **c** sketches of parametric CAD model; **d** comparison between the CT data-based CAD design and the segmented 3D volume of the actual chiton armour components; **e** virtually buckled FEA model of man-made armour; **f** a prototype of chiton-inspired armour (reproduced from [313])

The first approach relies on the imitation of biomaterial architectures in product design, where imaging data is analysed carefully, and image-based numerical models are established based on this data for elucidating the mechanics of material architecture under operating conditions. In this case researchers establish their numerical models inspired from natural materials using direct measurements of microstructural features, for instance, from CT images rather than converting 3D segmented image data directly into image-based meshes. This IBSim approach has been applied in investigations of wood [323], marine animals [313], balsa trunk and branches [324, 325], hedgehog quill [326] and spine [327]. New structures can be designed based on the architecture of these materials. For instance, a study took into account cancellous bone structure of human tibia as a bionic object when structurally designing a novel crash box with improved crashworthiness and energy absorption performance [228]. Similarly, thin-walled structures with superior crashworthiness features were designed using the biological structure of bamboo with energy absorption ability and the deformation behaviour of the new designs was simulated under longitudinal and lateral compression [328]. In the first approach, the ultimate design does not have to be in the same length scale as the inspired material. A comprehensive review has been conducted into bioinspired structures and materials [321]. These structures are often at a different length scale to that of the original biomaterial, therefore, the reviewers decided to organise the literature in terms of their energy absorption performance. To exemplify such applications, the wood structure of a Manchurian walnut tree was scanned by μCT to be converted into FEA models of the microstructure and, then, the anisotropic micro-structure was imitated on the macroscale by manufacturing with an AM process [323]. In another example, armours of Chitons, which are a family of marine animals, inspired artificial armours with increased flexibility and protective structures compared with conventional man-made armours which are highly rigid structures with flexibility and manoeuvrability trade-off [313]. The methodology of the research consists of three critical stages (i) imaging (ii) computational modelling and (iii) manufacturing of designs. Dimensions and geometry of basic components of chiton’s armour were quantified from SEM and X-ray images (Fig. 34a, b). In this type of modelling approach, computational structures for FEA simulations are generated from parametric CAD models, shown in Fig. 34c, originated from the imaging data and not direct discretisation of 3D segmented volume data. Fig. 34d illustrates the comparison between the CT data-based CAD design and the segmented 3D volume of the actual chiton armour components. An advantage of having a parametric computational model is the capability to test, virtually, many design scenarios in various loads and boundary conditions, such as compression (i.e., buckling) in Fig. 34e. This led to identifying an optimum design, performing all the necessary tasks (bending, buckling, stretching) properly without any functional loss. A prototype for a design of chiton-inspired armour (Fig. 34f) was fabricated with multi-material 3D printing AM technology and qualitatively tested.

Furthermore, balsa trunk and branches hosting vertical hexagonal columns (cellular material) can be implemented for engineering applications because of their ability to hold high stiffness under tension and shear [325], and strength under compression [326]. The cylindrical structure of hedgehog quills were the inspiration for stiffeners due to their bending, ovalisation and buckling resistances [326].

Other previous research has worked in the same length scale where the information used to build the computational structures was extracted from the CT images or an alternative imaging method. For example, X-ray images of hedgehog spine samples were acquired and rendered in 3D to obtain the detailed microstructural measurements to be used to generate discrete micro-accurate FEA models [327]. Differing to continuous models, where homogenisation processes are applied over the volume of interest, discrete models aim to capture physics of mechanical problems by imitating true architecture of materials to as high a resolution as experimental measurements have allowed. In the case of the hedgehog, longitudinal stringers and transverse central support plates were identified from X-ray images and this information was introduced into FEA models of spines [327]. Additionally, the imaging datasets of various biomaterials obtained from CT or μCT are available in online repositories such as Digimorph, Morphosource, Gigascience for the use of researchers [329] that can use the potential of biomimicry to develop their bioinspired products.

#### Virtual Qualification of Bioinspired Design Using IBSim on Manufactured Parts

**Fig. 35:**
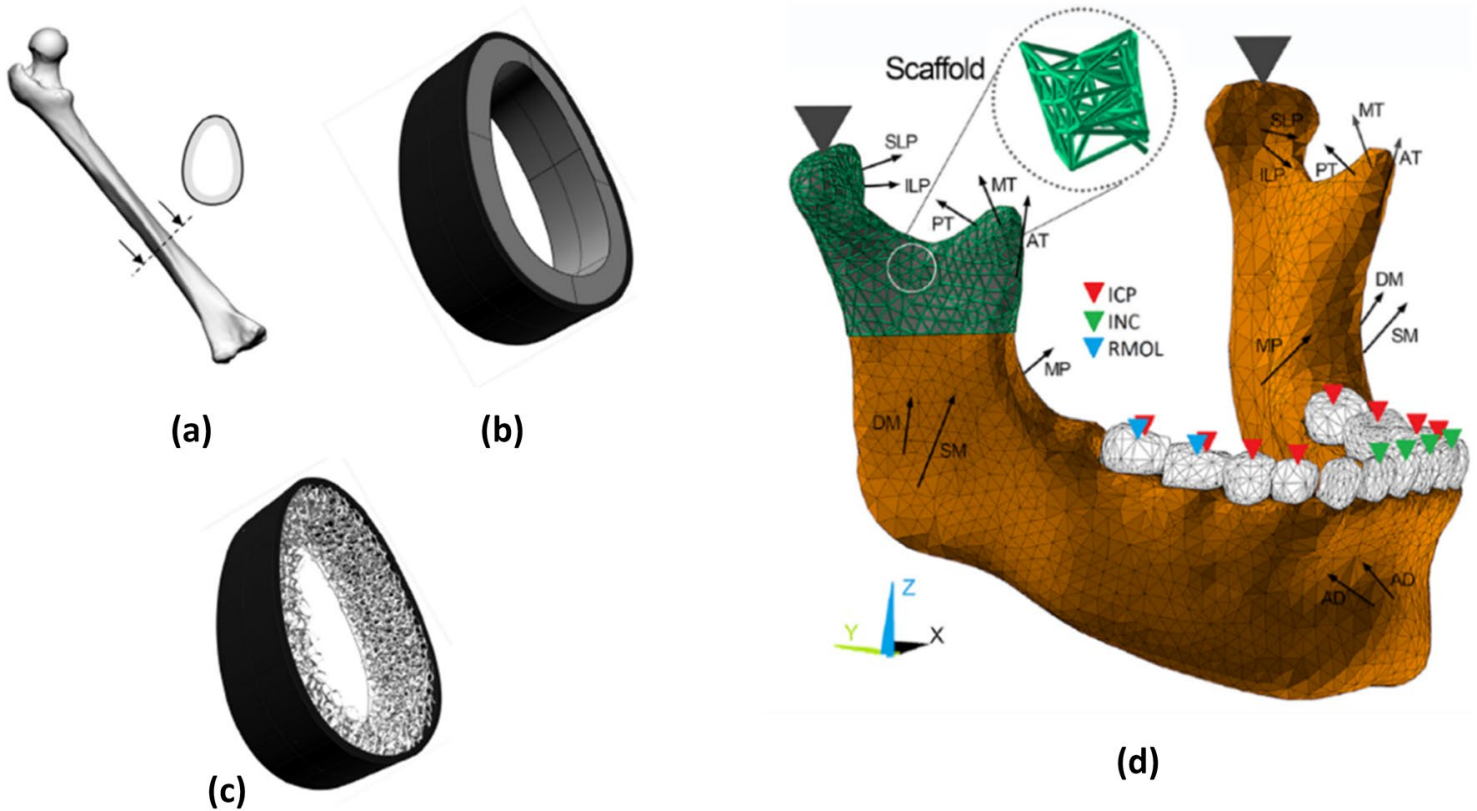
Design steps to generate a 3D porous scaffold: **a** selecting the implantable volume zone (or volume of interest), **b** 3D homogeneous model geometry, **c** inserting porous trabecular bone like scaffold [335]; **d** FEA model of a mandible, scaffold and tissue-engineered bone graft implants [222]

The second approach of IBSim in biomimicry makes use of the imagining data with high resolution simulations to virtually qualify the constructed biomaterial architectures [330–332]. The alternative architectures, which are analogues to the real biomaterials in terms of statistical data, can be manufactured as a replacement to the original material. Because IBSim can be performed non-destructively, it provides an opportunity to replicate virtual mechanical behaviour of biomaterials under numerous loading conditions on the same sample, an advantage when compared to destructive in-situ physical investigations such as time-resolved μCT [333, 334]. IBSim is used for developing body implants and improving their mechanical properties. One aim in designing such implants is to reduce weight by utilising their porous architecture while preserving functionality [222]. Fig. 35 shows two different cases of artificial porous scaffolds designed for a certain area in trabecular bone (CAD-based geometry) [335] and a mandible (hybrid CAD/image-based geometry) [336]. Porous micro or macro-structure is a major parameter influencing the mechanical properties, such as ultimate strength of implants. A research paper reported that customised porous titanium plates were manufactured to cover two human skulls having undergone trauma or disease [223]. Rigid cell foams are one type of porous scaffolds, which are frequently used in bioengineering applications such as metal bone replacement in orthopaedic applications [337], polymeric bones in real vertebra [338], glass–ceramics foam in orbital implants [330], zirconia scaffolds in bone tissue engineering [46]. They are designed to provide highly similar microstructural environments for clinical experiments. IBSim, therefore, can help to predict the mechanical response of artificial tissues or structures in order to prevent potential risks that can occur during and post medical operations.

In some biomedical applications, load-bearing tissues, such as trabecular bone, are replaced by artificial tissues or tissue constructs. μCT-based FEA models were generated and the mechanical behaviour was simulated to optimise the microstructural design of scaffolds by Jaecques et al. [339]. Geometrical parameters of open-porous titanium scaffolds with cubic, diagonal and pyramidal designs were numerically optimised to reach the elastic properties of human cortical bone, where minimum pore size was taken into account [220]. A set of scaffolds with high strength, stretch-dominated topologies (tetrahedron and octet trusses) for bone replacements were fabricated with L-PBF and tested to understand the influence of cell topology, pore-size, volumetric porosity on mechanical strength and bone in-growth [337]. The morphological deviations related to L-PBF technology was analysed via μCT. Triply periodic surface microstructures of Ti6Al4V were manufactured by a laser melting process and their mechanical properties were investigated with CAD-based and μCT-based FEA models [340]. These structures are lattices with high porosity, promising sufficient load bearing capacity for bone implants. Similarly, the mechanical properties of zirconia foams for bone tissue engineering was investigated with μCT-based FEA models, where noise in μCT images was removed and smooth boundaries were applied before construction of 3D zirconia foams [46]. The smoothing process retained the fabrication-induced pores, though artefact-based voids were eliminated. Importantly, it was found that the strength of image filtering has a non-negligible effect on the geometry, e.g., porous structures, and hence material properties like stiffness (Young’s moduli).

Porous scaffolds with different geometrical parameters can be produced by various manufacturing methods such as AM. Their simulation geometries, obtained from direct processing of X-ray images, can be used to search for the optimum architecture for cell growth and better mechanical performance. Scaffolds can be designed with various external geometries and various tortuous internal architectures in an idealised CAD environment; however, fabrication processes result in discrepancies, such as surface roughness and micropores, between the manufactured scaffolds and their CAD design [341].

Virtual topological optimisation of scaffolds might use IBSim and AM technologies, where voxel-based CAD geometries are constructed from 3D image data which are processed into STL surface geometries [218]. At this point, a topological optimisation algorithm, which considers a variety of loads and material parameters such as elasticity and plasticity in the design of scaffolds, can be applied to discretised CAD-based FEA simulations. The most efficient design is then selected to be fabricated with one of the AM technologies. In order to realise CAD geometries of ultimate designs into real-world parts, the CAD models are mathematically sliced into set of thin layers.

**Fig. 36:**
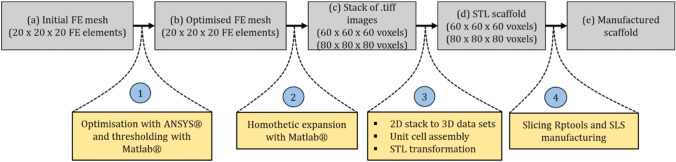
Steps from the design to manufacturing: **a** initial and **b** optimised FEA meshes, **c** stack of TIFF images, **d** STL images of the scaffold, and **e** manufactured scaffolds (redrawn from [219])

In order to provide adequate mechanical properties to porous scaffolds for biomimetic applications, they can be designed and simulated mechanically with CAD, then optimised by using AM for experimental characterisation to quantify performance parameters such as stiffness and permeability [219]. In the same paper, IBSim was used for virtual qualification of the manufactured part and compared with the expected values based on the initial CAD-based design. The focus was on Poly-e-caprolactone-4% hydroxyapatite porous scaffolds with various solid volume fractions which were numerically generated into voxel-based FEA models of scaffolds. Consequently, the FEA models were split into dataset of ‘TIFF’ images with higher resolution that are segmented to create STL scaffolds. STL files were then prepared for rapid prototyping of the scaffolds with L-PBF. However, the nature of rapid prototyping results in differences between the ‘as designed’ and ‘as manufactured’ scaffold geometries [337]. In light of their comparisons, architectures of scaffolds were optimised. Steps from the design to manufacturing of an optimised porous scaffold are explained in Fig. 36, with further details available in the original research article [219].

#### Direct Implementation of IBSim on Source Biomaterials

Borah et al. investigated: the roles of μCT and image analysis for a quantitative analysis of trabecular bone architecture; FEA for mechanical behaviour of bone at micro and macro-levels; physical replicas from rapid prototyping for enhanced visualisation [342]. This allows the researcher to understand the effect of bone microstructure on osteoporotic fractures [343]. μCT-based IBSim can be directly used for quantifying stress and strain analysis in actual bone tissues and scaffolds [46]. Performance of scaffolds relies on design and characterisation of their microstructure and, therefore, μCT is one of the key instruments in the microstructural characterisation [344].

**Fig. 37:**
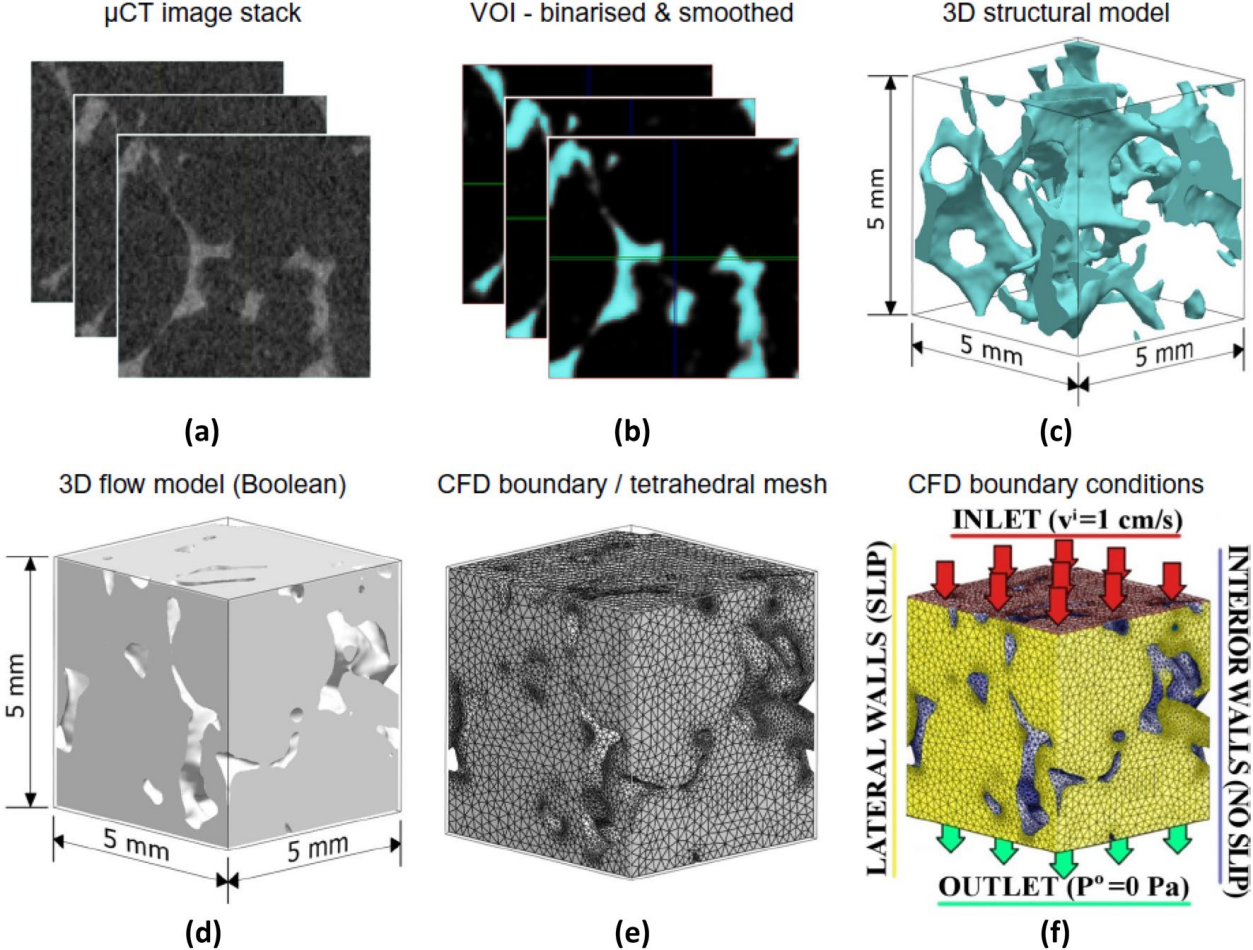
Flow behaviour over porous scaffold geometry directly obtained from CT images can be achieved with a list of steps: **a** stack of μCT images, **b** binarisation and smoothing of the selected volume of interest, **c** 3D reconstruction and segmentation of the structure, **d** Boolean operation over the structural geometry to produce the flow domain (i.e., CFD domain), **e** meshing process of IBSim CFD model, **f** assigning various boundary conditions (inlet velocity, outlet pressure and wall boundary conditions) [332]

To demonstrate the use of IBSim with foam materials, a commercial synthetic foam (open-cell) was considered as a replacement to human cadaveric bone Synthetic to simulate various in-vitro cases for bone infiltration (see Fig. 37) [332]. The procedure to be followed is listed as i) μCT scanning of the sample materials and saving the images in DICOM file format, ii) importing the images in Simpleware Software (Synopsys Inc.) and applying a list of postprocessing operations—in particular, noise reduction, smoothing, automated segmentation, iii) construction of foam volume, iv) Boolean operation to obtain complex flow volume, v) meshing the flow volume with tetrahedral elements, vi) applying prescribed boundary conditions, inlet flow velocity, outlet pressure and slip/nonslip boundaries. In another similar application of the direct use of IBSim, the peel from the Pomelo fruit, which has a foam-like hierarchical microstructure, was investigated by analysing image data from in-situ compression tests [334]. This research was carried out to inspire development of novel materials due to their high energy absorption efficiency. For inspiration of advanced biomimetic hydraulic systems, the flow mechanisms of a hydraulic joint in a spider leg was studied with CFD simulations through a commercial software ANSYS Fluent® where the flow models were extracted from direct processing of 3D μCT images and supported with appropriate boundary conditions of high-pressure areas such as inlet, joint and closed leg ends [345].

A review paper from Jones and Wilcox examined various strategies used to develop IBSim FEA models of human spines (in particular, vertebra, intervertebral disc and short spinal segments) [346]. Each model had three major steps: i) verification, ii) sensitivity analysis, iii) validation. These models were based on direct processing of images. In order to validate the IBSim results against the experimental tests requires accurate localised material properties rather than the global effective (or homogenised) properties which are usually obtained.

Multiple data sets for the same scaffolds at different length scales can be achieved with the IBSim approach. For instance, the exterior geometry of the scaffold can be generated with macro-details, and the interior geometry can be represented with microscale architecture [341]. For instance, polymeric beads were placed into the solid rods of ceramic scaffolds to characterise the effects of these defects on their fracture behaviour under uniaxial compression [347]. The scaffold macrostructure was scanned at low-resolution, whereas the rods including artificial defects were the region of interest and analysed with CT at a higher resolution. With this method, FEA models of segmented CT images with sufficiently fine meshes can give an account for deformation and fracture behaviour at two scales (micro and macroscales).

Permeability of AM scaffolds designed for healing bone tissues with defects was investigated through flow IBSim models [348]. Air or liquid permeability in accordance with flow behaviour relies on major geometrical microstructure parameters, for example, total porosity, pore shape and sizes, their interconnectivity and abundance [349, 350]. Permeability of porous scaffolds depends on nano and macrostructures and plays a key role in the biological performance of the material [350]. Accurate statistical analyses of these microstructure parameters can be carried out with IBSim. The permeability coefficients of porous scaffolds can be computed with virtual tests using CFD simulations of IBSim models in place of their physical counterparts. Results can be compared with experimental measurements for verification or simple models can be verified with analytical or empirical solutions from literature. Such investigations on CAD designs and actual scaffold geometries manufactured with AM were carried out to for skeletal tissue engineering [348]. The pressure and velocity fields of the scaffolds were computed using a commercial finite-volume based CFD code (Fluent 6.3®) for both the CAD and image-based models. The computed permeability of IBSim models were found to have a higher degree of accuracy than the CAD-based models when compared with the experimental measurements.

## Summary, Discussion and Conclusions

This review has set out to report on applications of IBSim within HVM. In doing so, discussion of the literature has been grouped into application spaces within HVM (and subdivided further within those subsections). That is, characterisation of materials and manufacturing techniques, quantifying the impact of ‘real’ geometries compared with idealised ones, customisation of products, and biomimicry. As a ‘first of a kind’ review in this field there was a significant volume of literature to consider. As such, the papers presented in this work is not an exhaustive list but presents important milestones in the adoption of IBSim within HVM.

For the research using IBSim for material characterisation, these works investigated materials such as composite materials (both fibrous and aggregate), AM materials, and foams. The feature in common between these material types were that they all exhibited non-negligible variations from one instance to another, either by design or as a by-product of manufacturing processes. They were mostly well-suited for XCT imaging, due to a beneficial attenuation contrast to facilitate identifying geometric features. Where this does not hold true is for fibre composites, where the fibres and matrix use either the same or similar material, or AM materials with low pore volume fractions. There is ongoing research within the imaging field to use methods such as phase contrast CT to improve such data [351]. The literature demonstrated that the majority of effort is in characterising mechanical behaviour or the permeability of materials. The other main areas of interest were thermal and electrical conductance. A substantial proportion of the work focussed on using RVEs or unit cells from a region of interest within the imaged material to reduce computational expense.

Understandably there was a significant overlap with areas of interest for characterisation of materials. The main distinction was that efforts using IBSim in this field focussed on particular features (e.g., a class of defect) to better understand the cause of their formation. The majority of the features of interest (e.g., pores) are inherent by-products of the manufacturing methods, whilst others were caused by unexpected issues during manufacturing. IBSim is being used to better understand what level of these features can be tolerated, and in some cases used to improve performance (such as surface roughness to increase heat transfer) or to improve manufacturing efficiency. Examples were observed where artificially induced defects were included to investigate the impact of defects on behaviour in greater detail. Of the research observed, many made recommendations based on IBSim investigations as to how processes may be improved, however, none were found that closed this loop by implementing the improvements suggested within their own studies.

When considering the applications of IBSim to investigate the impact of deviations from the ideal on performance and on a sector-by-sector basis, literature broadly fell into three sectors: medical, energy, and aerospace. It was observed that, until recently, IBSim has generally been used to investigate material coupons or regions of interest within larger components rather than performing IBSim analysis of whole components. There is a wealth of literature using IBSim in the medical sector to investigate aspects of the human body (e.g., mechanical behaviour of bone or flow through the cardiovascular network). The scope of this review was restricted to examples from the medical sector, which also included HVM, e.g., the design or use of an implant. From the volume of available literature to date, it can be observed that this is currently the main sector where IBSim is used routinely, and a significant portion of this is for patient specific applications (see below). For the energy sector, the main area where IBSim has been used is in the characterisation of materials in batteries and the development of novel materials. There are some examples where IBSim is used for NDT/NDE on the component scale, however, this approach has not yet reached maturity. For the aerospace sector a similar distribution of research was observed, i.e., that IBSim is predominantly used for materials or process development, with some examples in component scale NDT/NDE.

As noted, where IBSim is used in HVM for biomedical purposes, a significant proportion of this is patient specific applications. These applications include healthcare, personalised medicine, prosthetics, and orthotics. Available literature shows how IBSim has been used to design implants, plan procedures, and monitor deployment of personalised products over time. Compared to applications of IBSim for HVM in other sectors presented in this review, these examples are comparatively mature. There is, however, still significant potential for further uses, and further work is required to achieve acceptance of highly personalised approaches with regulatory bodies. Examples were also observed where IBSim was used to personalise or customise sport, lifestyle, and automotive products outside biomedical applications. For example, to improve the efficiency of protective sport equipment for an individual or to improve the accuracy of digital vehicle crash testing using virtual humans from image data.

Stemming from the fact that IBSim has its origins in biomedical engineering, it was observed that there was a significant volume of research using IBSim for HVM in relation to biomimicry and bio-inspired engineering design. These works were not only inspired by aspects of the human body, but also the natural world. Hundreds of millions of years of evolution have generated structures that are highly efficient at their given functions. The observed literature demonstrates that IBSim is enabling researchers to investigate how these organically generated structures perform in a detail not previously possible. The work surveyed using IBSim with biomaterials broadly fell into three categories:

- Use of 3D image data from biomaterials to inspire engineering designs, which in turn are tested with conventional simulation analysis methods.
- Virtually testing bioinspired parts ‘as manufactured’ to gain insight at the microscale.
- Direct implementation of IBSim on source biomaterials for a better understanding of their behaviour.

Despite the broad range of application spaces where IBSim has been observed to be used within HVM, there are a number of commonalities which are noteworthy. Firstly, the imaging and simulation methods observed in the research were predominantly XCT and FEA or CFD. This can be attributed to the fact that this review focuses IBSim applications in HVM, i.e., a sector where the use of CAD-based FEA/CFD is already commonplace for component design, and there is significant year-on-year growth of XCT for metrological characterisation. That is, where IBSim was observed to be used, the imaging and simulation techniques were already being employed and IBSim was a method to extract additional value from data that was already routinely generated. By combining these commonly used methodologies, it is observed that IBSim has been providing researchers with improved levels of accuracy with predictive simulations that were previously unobtainable.

**Fig. 38:**
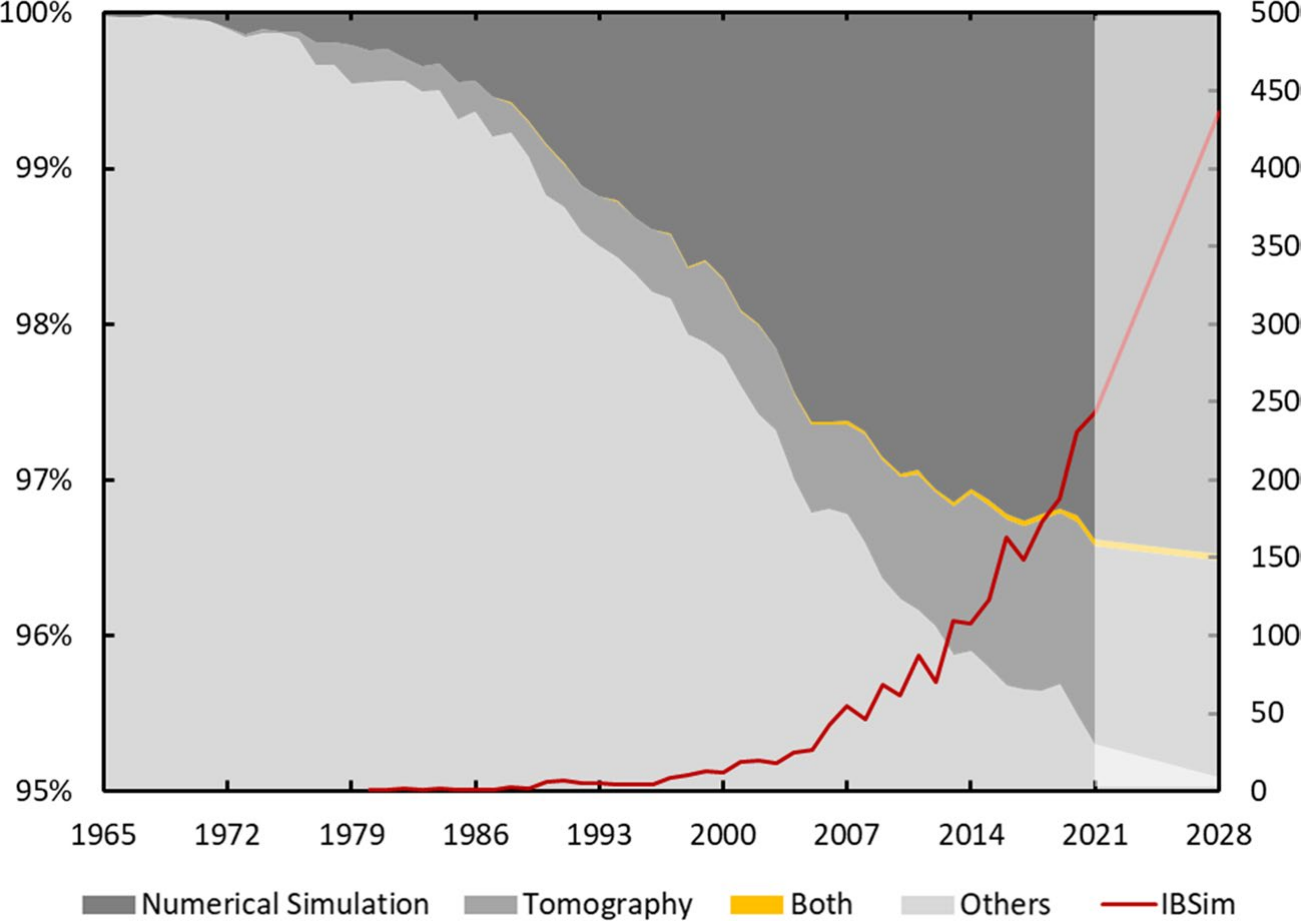
Change in publications by year: (area) as a percentage of the total publications considered; (line) absolute number. The area data was found using the search terms (“numerical simulation” OR “computational engineering”); (“tomography”); ‘Both’ denotes, the crossover of these two search terms. The line data was found using the search term ‘(tomography AND “computational engineering”) OR (tomography AND “numerical simulation”) OR (“image-based simulation” OR “image-based modelling”)’. Data collected from Scopus whilst restricting the search to the ‘Engineering’ and ‘Materials Science’ subject areas and the ‘Article’ document type. Data for 2021-2028 are extrapolated using a 3^rd^ order polynomial

This observation is broadly supported by the recent growth in publications on these topics, see Fig. 38. This data was collected from the Scopus database of literature, with the searches being restricted to the ‘Engineering’ and ‘Materials Science’ subject areas and the ‘Article’ document type. To account for the general year-on-year growth in published research articles, in Fig. 38 (area) the changes are displayed as a percentage of the total number of papers published in the subject areas in question. The ‘IBSim’ curve in Fig. 38 (line) is given as the absolute number of articles published. Data for 2021–2028 are extrapolated using a 3^rd^ order polynomial. The search terms used were:

- Numerical Simulation = “numerical simulation” OR “computational engineering”.
- Tomography = “tomography”.
- Both = (“numerical simulation” OR “computational engineering”) AND “tomography”.
- Others = The remaining articles published in the ‘Engineering’ and ‘Materials Science’ subject areas.
- IBSim = ‘Both’ search terms OR “image-based simulation” OR “image-based modelling”.

By combining these commonly used methodologies, it is observed that IBSim has been providing researchers with improved levels of accuracy with predictive simulations that were previously unobtainable.

There were also some caveats, limitations and challenges that were common to many studies. The availability of simulation geometries at newly achievable resolutions in particular brought a set of challenges for consideration. A major one of these is which material properties should be used and how to collect these. This is because simulations, such as FEA, conventionally treat materials as homogeneous and thus use experimental data that is typically collected on macroscale samples, whereas those material properties do not necessarily hold true on the microscale. Additionally, simulations performed on the same size of component, but at a significantly higher resolution (i.e., including micro-features within macro-models), increases the computational demands both in terms of solving the equations and visualising the results. For a typical FEA analyst, CAD-based simulations are mostly carried out using desktop PCs which have higher power hardware that those used for conventional office work. However, these are only moderately powered relative to high-end workstations available on the market and significantly less powerful than supercomputing facilities. On these types of machines, the lack of available RAM and computing power can make it unfeasibly slow or, at worst, impossible to perform IBSim research [352]. Another recurring theme was that simulation results were highly sensitive to image quality and the associated post-processing of images. A significant factor in this is variability between imaging systems (even of the same type) and the subjectivity of operators.

Based on the observed literature, the reported benefits in applying IBSim within HVM for virtual testing can broadly be categorised as follows:

- Can use to accurately replicate laboratory experiments for a direct comparison of results. This is beneficial to facilitate validation of numerical models or to gain additional insight to experimental results. That is, experimental results tend to give the overall global response (or sparse local data), whereas it is possible to investigate local response through the full volume with IBSim.
- Can perform virtual tests not possible experimentally. This can be of benefit to perform a simplified experiment which more rapidly measures the feature of interest (e.g., applying a thermal gradient across a volume to measure effective thermal conductivity) or to apply conditions that would be too difficult to apply in a laboratory (e.g., extreme loads). Similarly, laboratory tests are often set up to approximate in-service conditions, whereas these can be applied directly to the real geometry with IBSim without the constraints of a lab.
- Can perform a series of virtual destructive tests on the exact same sample to remove variability of geometry (and the consequential variability on results). Similarly, if sufficient computing power is available, many virtual tests can be run in parallel to accelerate the generation of data without the requirement to buy duplicate testing hardware.

The majority of research published in peer review journals using IBSim applies the technique to coupon scale samples or RVEs. Alternatively, the research uses a hybrid CAD+IBSim approach where IBSim is used to investigate a region of interest, or a component is designed by CAD to conform to an image-based geometry of the object with which it is to interact. Comparatively, there is very little literature on component scale modelling done purely with IBSim. Although it is difficult to directly demonstrate causality, it is the authors’ opinion this can be attributed to a few different factors. For example, a highly detailed IBSim model can be many orders of magnitude more computationally expensive than a CAD-based equivalent, leading to requiring national-level high-performance computing facilities to which not all researchers readily have available access. Smaller scale ROI or RVE models can be used to mitigate this issue. Imaging full scale components, then, has two associated challenges. Firstly, as the size of the component increases, the level of achievable resolution often reduces. This could mean that the micro-scale features of interest which have a non-negligible impact on the component performance might not be resolvable in a volumetric image of the whole component. Secondly, for techniques like XCT, larger components lead to higher signal attenuation. If the component is sufficiently large it could mean that volumetric imaging is no longer possible with the same equipment. Despite this, it is known to the authors that there is an appreciable amount of R&D activity occurring within the industrial sector which utilises IBSim for component scale modelling. Unfortunately, this work is considered valuable intellectual property and therefore not published in peer review journals.

Another point noted concerning the current state of IBSim literature is that none reported implementing the improvements suggested by their own studies. Due to the detrimental influences of different defect types within HVM, there is a strong need for defects to be minimised or mitigated by quality control (e.g., ensuring clean material feedstock to limit inclusions, ensuring a stable manufacturing process, etc.) or by process optimisation (adjusting process parameters e.g., power or scan speed, etc.). The extent to which this process optimisation is required depends on the strength of the influence, i.e., what is the effect of the defect exactly on the mechanical performance, and what is the expected mechanical performance of the part? This is where IBSim can be useful in combination with experimental work, to reveal for a specific material, geometry, and combination of defects, what the influence is on the mechanical properties and evaluate whether this is acceptable or not. This is further possible not only for improving manufacturing processes, but also for evaluating post-processing and wear or degradation after use. Post processing of metal parts often include heat treatment, machining, and sometimes more advanced processes such as hot isostatic pressing, which improves the microstructure and closes porosity, resulting in improved mechanical properties [353, 354]. IBSim may be used to evaluate the size of pores or other defects after processing or due to degradation of the material and evaluate the effect of the defect on the expected performance of the part. For IBSim to demonstrate its true value it is important that future work is published reporting measured benefits gained rather than only potential ones.

Finally, it is worth noting that a significant proportion of the research in the literature using IBSim does so within the highly regulated medical and aerospace sectors (see following sub-section for more details). These sectors are, understandably, known as being risk averse, however, they are also known for being pioneers of new technology. For IBSim, having a growing body of work from within these sectors should lead to increased confidence as the technique matures, which will support wider adoption in other regulated sectors such as energy.

### Future Trends

In this section, the authors present developments in associated fields of research and discuss how these could have a direct impact on the future use of IBSim. These are divided into developments which might bring about evolutionary and revolutionary changes presented in their own sub-sub-sections.

#### Evolutionary Changes

The easily predictable evolutionary advancements are those associated with improvements in hardware (both imaging and computational) and software, which are continually being gradually improved and are included in publicly available manufacturer development roadmaps. For example, manufacturers of imaging apparatus regularly release new versions that produce data with a higher resolution or improved image quality (e.g., reduced noise). This is obviously beneficial for producing more detailed volumetric images, or images at the same resolution, but producing results with a greater level of confidence. A by-product of these improvements is the ability to perform faster imaging for a higher throughput, something of great value to researchers using time-resolved imaging. For XCT in particular, a development of value for HVM is the availability of X-ray sources capable of higher energies. This is allowing researchers to image larger components or those made from materials that are high attenuators of X-rays. Similarly, computing hardware is constantly improving, allowing processing at faster rates and for larger datasets, both for imaging and simulation algorithms. Furthermore, there is ongoing research into improving algorithms for more efficiently utilising computational hardware (e.g., better parallelism including use of GPUs). Furthermore, algorithms are being developed to bring additional benefits, e.g., reduced artefacts during image reconstruction or higher simulation accuracies with adaptive meshing. There is also research into a method to convert 2D image projections directly into FEA meshes [355], which would remove the need for many of the interim workflow stages. The limitations caused by computational expense was mentioned in a significant proportion of papers as a factor leading to the choice to investigate ROIs. As already noted, this review has identified that IBSim is currently only being used in a limited way on the component-scale. The authors of this review believe there will be a significant shift towards more activity on the component scale through wider availability of higher power XCT devices and further improvements in computing power.

Other potential evolutional developments of IBSim are related to the way in which the techniques that are part of the workflow are being implemented within the HVM sector. For example, FEA is now a common tool in HVM, and, therefore, a set of ‘best practices’ have developed within the community that uses them, which are often formalised as international standards, e.g., in aerospace [356]. Since the use of advanced imaging techniques such as XCT in HVM is a relatively recent addition, the standards surrounding the methodologies are still in their infancy. As these mature this will facilitate repeatability in results, both in the individual method and for IBSim that combines them. It is important for the acceptance of IBSim within HVM that there is industry-level confidence in each of the workflow components.

It is also important to consider the apparent conflict in requirements between regulation and personalisation. For example, in personalised medicine, when the Federal Food, Drug, and Cosmetic Act (FDA) was passed in 1938, the term “Personalised Medicine” had not yet been coined. The standards for FDA approval for regulating medical devices (including laboratory tests) were established for traditional products in 1976, but the complexities associated with obtaining approvals for personalised medical products have since proved challenging. This echoes the challenges also seen in other highly regulated sectors, e.g., aerospace, whereby ‘designs codes’ and standards exist with the aim of ensuring a predictable performance through prescribed approaches (i.e., constraints) in design and manufacturing. This is in stark contrast to the approach of IBSim, which is to yield the same level of performance prediction whilst allowing design freedom.

At the core of precision medicine lies diagnostic tests and devices, but the regulatory classification of such products varies globally [357, 358]. Each regulatory agency has a clearly defined definition of what constitutes a custom-made device (CMD) or personalised medical device (PMD). For example, in the UK, any mass-produced device adapted to specific patient requirements post-production does not fall under the UK Medical Device Regulation definition for CMDs (e.g., optical glasses, patient-fitted wheelchairs, hearing aids and orthotic braces). Furthermore, it excludes mass-produced devices manufactured via industrial processes, even if produced according to written prescriptions [359]. The European Medical Device Coordination Group (MDCG) have also ensured that the manufacturers are solely accountable for patient-matched devices in terms of design, safety, performance and regulatory compliance [360].

The primary issues of these regulatory agencies involved concerns as to whether patients should be able to purchase and use unapproved or unregulated tests; and whether manufacturers can be trusted to market tests that conform to standards for safety and efficacy if the process of regulating these tests is too slow and expensive. Navigating these convoluted regulatory pathways can be challenging, and whilst the guidance for CMDs has improved these past few years, the regulation regarding a completely personalised part is currently impeded by incoherence between policymakers and regulators [361]. New regulatory reforms, however, specific to CMD’s (e.g., Software as a Medical Device (SaMD), PMDs and Medical Device Production Systems (MDPS)) are beginning to form, which highlights that these changes are occurring, albeit slowly [357]. A similar response is being observed in other sectors.

The final way in which IBSim is evolving is the application space in which it is being used, that is, the products being manufactured that are driving a demand for advanced characterisation methods with increased accuracy. A continual desire to produce more efficient products is bringing about the use of increasingly advanced materials with complex behaviours using novel manufacturing methods. In addition to this there is an increasing demand for personalisation of products. The combination of these factors presents an increasing stream of new opportunities where there exists a significant variation from part to part, or for those which are unique.

This review has shown that IBSim is ideally suited to this, and it has been found to play a critical part in obtaining patient- or subject- specific 3D geometry, where the ability to run multiple simulations that closely mimic the in-vivo response of the human body, or of a real-life scenario, are invaluable. The continued advancement and availability of imaging techniques, coupled with open access software and AM, will further enhance research and, subsequently, products in these areas. The personalisation fulfilment is, therefore, slowly leading to machine learning-driven intelligent configuration, and Industry 4.0-driven on-demand production, sometimes using manufacturing-as-a-service or 3D printing.

#### Revolutionary Changes

The prediction of revolutionary advancements in any technology is inherently more challenging; however, machine learning (ML) is one such disruptive technique that has been noticeably transforming most scientific fields over the past decade. There is much recent research with relevance to IBSim. In imaging, for example, optimising imaging setup [362] and the reconstruction of volumetric images [363] to improve scanning speed and image quality by orders of magnitude. Machine learning is proving to be well-suited to automate tasks. For example, automation of image segmentation not only speeds up this section of the workflow by orders of magnitude, but, for the first time, generates reproducible results [364, 365]. For simulations, IBSim offers the potential of actual ‘digital twins’, whereby each manufactured part has a digital equivalent capable of providing real time feedback for an in-service product. This is challenging for conventional CAD-based simulations and even more so for IBSim using FEA models. However, ML is being used to produce FEA surrogate models capable of making predictions in a fraction of the time of a full simulation [366]. A recent paper by Ezhov et al. proposed an AI system based on deep learning methods for dental diagnosis with CBCT. The AI system was found to significantly improve the diagnostic capabilities of dentists and has the potential to augment the dentists’ routine clinical practice [367]. The full realisation of these applications of ML will transform the potential of IBSim from being a technique requiring significant resource to use, preserved for the most well-equipped laboratories, to one that could be commonplace in a smart factory of the future.

A technological advancement that offers a significant opportunity for IBSim is augmented reality (AR). In the modern, highly competitive manufacturing environment, the application of AR consists of an innovative and effective solution to simulate, assist, and improve the manufacturing and maintenance processes. Today, a growing number of applications based on AR solutions are being developed for industrial purpose. A systematic review by Baroroh et al. reported on recent AR applications in smart manufacturing from a human–machine interaction perspective [368]. In another review, Bottani et al. reviewed the literature from 2006 to 2017 to identify the main areas and sectors where AR is currently deployed, describe the technological solutions adopted, as well as the main benefits achievable with this kind of technology [369]. In particular examples, 3D scanning is being used in conjunction with AR, for example, to simulate virtual ‘try-on’ technology, where fit and size issues of mass customised men’s jackets have been explored using 3D body scanning and 3D virtual simulation technology [370]. AR-based design customization of footwear for children is also widely documented [371]. Presently, the combining of this with detailed IBSim is likely to be too computationally challenging, however, using this technology alongside ML surrogate IBSim models could allow real-time feedback to the user (which could be human, or an AI-driven robot) that predicts the outcome of a rapidly developing situation, e.g., during layup of fibre laminates in composite manufacturing.

Another field that may bring transformative change is the introduction of novel imaging techniques able to generate rich image data which includes additional information about the material within the sample, analogous to imaging with backscattered electrons, energy dispersive spectroscopy, and secondary electrons in SEM. Not only do these methods promise to yield information sufficient to generate volumetric maps of material types, but provide additional information such as the material’s phase, their stress state, and porosity of sizes lower than the image resolution [372–376], all of which can be used to greatly enhance the predictive capability of IBSim.

The use of IBSim within HVM emerged in the early 2000s but exhibited a low rate of growth during that first decade. During the 2010s, there was wider use within academia across a broad range of research fields, coinciding with wider availability of volumetric imaging hardware. The authors of this review confidently believe that IBSim will enjoy widespread growth within the industrial sector during the 2020s and become an invaluable NDT/NDE method as part of the prevalence of Industry 4.0.

## Abbreviations

AM: Additive manufacturing
AR: Augmented reality
CAD: Computer aided design
CAM: Computer aided manufacturing
CBCT: Cone-beam computed tomography
CDAM: Cyber design and additive manufacturing
CDM: Continuum damage models
CFD: Computational fluid dynamics
CFRP: Carbon fibre reinforced plastics
CMD: Custom-made device
CPU: Central processing unit
CT: Computed tomography
DIC: Digital image correlation
EBM: Electron beam melting
FEA: Finite element analysis
FIB: Focussed ion beam
FVM: Finite volume method
GDL: Gas diffusion layers
GPU: Graphical processing unit
GTN: Gurson–Tvergaard–Needleman
HVM: High-value manufacturing
IBSim: Image-based simulation
LBM: Lattice Boltzmann methods
L-PBF: Laser powder bed fusion
MDCG: Medical device coordination group
MDPS: Medical device production systems
ML: Machine learning
MMPS: Modified-moving particle semi-implicit
MPL: Microporous layer
MRI: Magnetic resonance imaging
NDE: Non-destructive evaluation
NDT: Non-destructive testing
OSSM: Optical serial sectioning microscopy
PEM: Polymer electrolyte membrane
PMD: Personalised medical device
R&D: Research and development
RAM: Random access memory
RANS: Reynolds-averaged Navier–Stokes
RVE: Representative volume element
SaMD: Software as a medical device
SEM: Scanning electron microscopy
SLS: Selective laser sintering
TEM: Transmission electron microscopy
TRL: Technology readiness level
XCT: X-ray computed tomography
XFEM: Extended finite-element method
μCT: Micro computed tomography
STL: Standard tessellation language

## Footnotes

1 The term FEA is often used interchangeably with Finite Element Method (FEM) although they differ slightly in meaning. For consistency FEA will be used throughout.
